# Design, synthesis, and bioevaluation of novel unsaturated cyanoacetamide derivatives: In vitro and in silico exploration

**DOI:** 10.1016/j.mex.2024.102691

**Published:** 2024-04-09

**Authors:** Kabir M. Uddin, Mehnaz Hossain Meem, Mokseda Akter, Shofiur Rahman, Mahmoud A. Al-Gawati, Nahed Alarifi, Hamad Albrithen, Abdullah Alodhayb, Raymond A. Poirier, Md. Mosharef H. Bhuiyan

**Affiliations:** a Department of Biochemistry and Microbiology, North South University, Bashundhara, Dhaka 1217, Bangladesh; b Bioorganic and Medicinal Chemistry Laboratory, Department of Chemistry, University of Chittagong, Chattogram 4331, Bangladesh; c Biological and Environmental Sensing Research Unit, King Abdullah Institute for Nanotechnology, King Saud University, Riyadh 11451, Saudi Arabia; d Research Chair for Tribology, Surface, and Interface Sciences, Department of Physics and Astronomy, College of Science, King Saud University, Riyadh 11451, Saudi Arabia; e Department of Chemistry, Memorial University, St. John's, Newfoundland A1B 3 × 7, Canada

**Keywords:** α, β-unsaturated 2-cyanoacetamide;Knoevenagel condensation, Microwave irradiation, Molecular docking, MD simulation, FTIR and NMR analysis, Density Functional Theory (DFT), *in silico* simulations, and in vitro experiments for the investigation of α,β-unsaturated cyanoacetamides

## Abstract

In this study, we synthesized novel α,β-unsaturated 2-cyanoacetamide derivatives (**1–5**) using microwave-assisted Knoevenagel condensation. Characterization of these compounds was carried out using FTIR and ^1^H NMR spectroscopy. We then evaluated their in vitro antibacterial activity against both gram-positive and gram-negative pathogenic bacteria. Additionally, we employed *in silico* methods, including ADMET prediction and density functional theory (DFT) calculations of molecular orbital properties, to investigate these cyanoacetamide derivatives (**1–5**). Molecular docking was used to assess the binding interactions of these derivatives (**1–5**) with seven target proteins (5MM8, 4NZZ, 7FEQ, 5NIJ, ITM2, 6SE1, and 5GVZ) and compared them to the reference standard tyrphostin AG99. Notably, derivative **5** exhibited the most favorable binding affinity, with a binding energy of -7.7 kcal mol^−1^ when interacting with the *staphylococcus aureus* (PDB:5MM8), while also meeting all drug-likeness criteria. Additionally, molecular dynamics simulations were carried out to evaluate the stability of the interaction between the protein and ligand, utilizing parameters such as Root-Mean-Square Deviation (RMSD), Root-Mean-Square Fluctuation (RMSF), Radius of Gyration (Rg), and Principal Component Analysis (PCA). A 50 nanosecond molecular dynamics (MD) simulation was performed to investigate stability further, incorporating RMSD and RMSF analyses on compound 5 within the active binding site of the modeled protein across different temperatures (300, 305, 310, and 320 K). Among these temperatures, compound **5** exhibited an RMSD value ranging from approximately 0.2 to 0.3 nm at 310 K (body temperature) with the 5MM8 target, which differed from the other temperature conditions. The *in silico* results suggest that compound **5** maintained significant conformational stability throughout the 50 ns simulation period. It is consistent with its low docking energy and in vitro findings concerning α,β-unsaturated cyanoacetamides.

Key insights from this study include:

• The creation of innovative α,β-unsaturated 2-cyanoacetamide derivatives (**1**–**5**) employing cost-effective, licensed, versatile, and efficient software for both in silico and in vitro assessment of antibacterial activity.
• Utilization of FTIR and NMR techniques for characterizing compounds **1**–**5**.

## Graphical abstract

**Figure:**
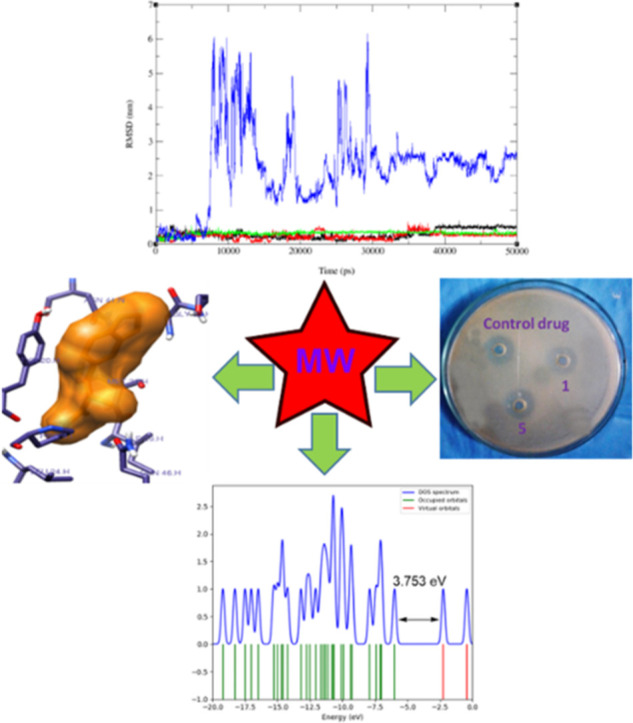

Specifications table

**Table:** 

Subject area:	Chemistry
More specific subject area:	Biomolecular simulations, structural bioinformatics, systems biology, and inhibition mechanisms for disease-causing microorganisms, with a focus on α,β-unsaturated cyanoacetamides.
Name of your method:	FTIR and NMR analysis, Density Functional Theory (DFT), *in silico* simulations, and in vitro experiments for the investigation of α,β-unsaturated cyanoacetamides.
Name and reference of original method:	Not applicable
Resource availability:	Methods: Fourier transform infrared spectrophotometer (FTIR), NMR spectroscopy, Density Functional Theory (DFT), in silico tools, and in vitro experimentation.

**Method details**

## Background

The widespread utility of α,β-unsaturated 2-cyanoacetamide derivatives in medicinal chemistry is well-established, as they serve as essential building blocks for synthesizing bioactive compounds, thereby facilitating the development of innovative pharmaceutical agents [1], [2], [3]. One noteworthy advancement in organic synthesis is the application of microwave-induced methods, exemplified by the microwave-induced synthesis of various quinoline derivatives, which exemplifies the potential of green methodologies in various chemical disciplines [4,6]. Green chemistry strives to minimize the use of hazardous chemicals and energy-intensive processes while optimizing resource efficiency [4], [5], [6], [7]. Microwave-assisted organic synthesis [4], [5], [6], [7], [8], [9], [10], [11], [12] proves advantageous by enhancing yield, reducing reaction times, and minimizing the generation of hazardous by-products. This technique is particularly valuable for conducting solvent-free reactions, addressing concerns related to toxicity and flammability often associated with traditional solvents. In line with sustainable practices, the environmentally conscious approach of employing green chemistry principles has garnered significant attention in the synthesis of unsaturated 2-cyanoacetamide compounds. This eco-friendly approach has been effectively applied to the synthesis of 2-cyanoacetamide derivatives, resulting in environmentally sustainable processes. These derivatives find applications in a diverse array of fields, encompassing anticancer, antifungal, antibacterial, and anti-corrosion applications, as well as in biology, industry, agriculture, medicine, and chemical synthesis [13], [14], [15], [16], [17], [18].

The synthesis of α,β-unsaturated 2-cyanoacetamide derivatives via Knoevenagel condensation is a classical bond formation reaction is widely employed technique in organic synthetic chemistry and several reaction conditions have been demonstrated [19], [20], [21], [22], [23]. This chemical process involves the interaction of aldehydes with active methylene compounds bearing a (C=C) group, ultimately forming these valuable derivatives. The presence of the active methylene group is pivotal for the successful execution of this reaction, as it plays a pivotal role in facilitating the condensation process. Knoevenagel condensation reactions have been facilitated using a diverse array of catalysts, encompassing ZnCl_2_ [24], clays [25], silica gel [26], natural catalysts [27], ammonium acetate (NH4OAc)-basic alumina [28], microwave irradiation, and thermal heating conditions [29]. Furthermore, NiCu@MWCNT has demonstrated its efficacy as a catalyst [30], operating under mild reaction conditions. Utilizing microwave irradiation in these reactions confers several benefits, including shortened reaction times, increased product yields, enhanced selectivity, and a solvent-free approach that aligns with eco-friendly principles. These advantages collectively contribute to developing a cost-effective and easily accessible method for organic synthesis. As an illustration, Bhuiyan and colleagues have recently introduced a practical approach to produce chalcones with excellent yields in a solvent-free environment, employing microwave irradiation [31]. In a recent study, Uddin and co-authors employed microwave-assisted Knoevenagel condensation to synthesize two innovative series of derivatives **(1** − **6**), derived from benzylidenemalononitrile and ethyl 2-cyano-3-phenylacrylate [12].

Limited computational investigations have been carried out on a range of compounds, including derivatives of 3-cyano-2-pyridone [32], 1,3,5-triazine-2,4-diamine [33], as well as 1H-indole-2,3‑dione (isatin), (2E)−1,3-diphenylprop-2-en-1-one (chalcone) [33], and 10H-acridin-9-one (acridone) [34]. Hernández et al. [32] reported an efficient and straightforward method for synthesizing 3-cyano-2-pyridone derivatives (6a-f) through the oxidation of 3,4-dihydropyridin-2-one. They also investigated the steric variations that result in distinct docking behaviors for nifedipine, observing that all compounds exhibited similar free energy of binding during docking studies. In a recent study, Uddin et al. [12] synthesized two sets of compounds, specifically benzylidenemalonitrile and ethyl 2-cyano-3-phenylacrylate derivatives **(1**−**6**), employing the Knoevenagel condensation method. Additionally, they carried out comprehensive *in silico* analyses on these synthesized compounds, encompassing molecular docking and molecular dynamics (MD) simulations. To underscore the potential of these compounds as potent drugs for specific activities, we conducted molecular docking and MD experiments involving a variety of proteins. Consequently, using unsaturated cyanoacetamide derivatives as bioactive agents against human pathogenic microorganisms offers substantial prospects for application in biological research. Nevertheless, it remains imperative to conduct further research to fully exploit their potential and advance them into safe and effective treatments for human use.

**Scheme 1:**
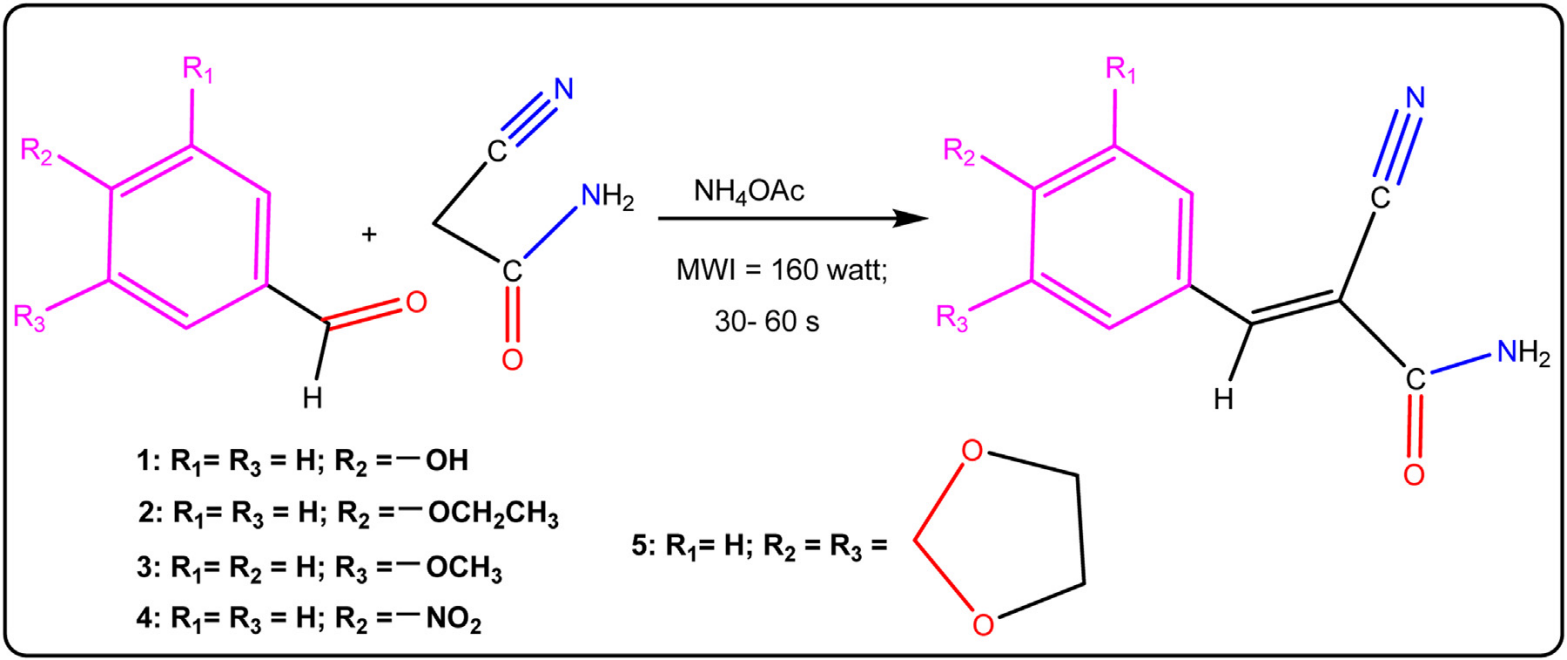
Synthesis of α,β-unsaturated 2-cyanoacetamide derivatives (**1** − **5**).

This study involves the synthesis of a range of novel biological compounds, specifically α,β-unsaturated cyanoacetamide derivatives (**1**−**5**), achieved through the Knoevenagel condensation reaction. The chemical structures of these compounds were elucidated through rigorous spectroscopic analysis, and their representation can be found in Scheme 1 and Tables S1 –– S5 in the supporting information (SI) illustrate the synthesized compounds (Table 1). Significantly, no computational investigations have yet been conducted to foresee the physicochemical, spectral, and biological attributes of the freshly synthesized derivatives (**1**–**5**). The primary aim of this study is to perform *in silico* analyses on the synthesized compounds, encompassing molecular docking and MD simulations. Additionally, the study includes the assessment of drug-likeness and pharmacokinetic properties of the chosen compounds through ADMET prediction [34] and SwissADME [35] profiles. The primary aim of this study is to advance our understanding of the binding affinities of the newly synthesized compounds (**1**–**5**), a critical factor with significant potential implications for their biological applications. Additionally, we have conducted computations to evaluate the thermophysical properties of these compounds, assessing their conformational stability. Furthermore, DFT calculations were carried out to determine critical parameters such as molecular structures, electronic density, NBO charges, frontier molecular orbitals, chemical hardness (η), and chemical potential (µ). These parameters are commonly utilized to elucidate reactivity and stability, thus providing valuable insights to complement the experimental investigation.

**Table 1:** Structures of α,β-unsaturated 2-cyanoacetamide derivatives (**1** − **5**).

**1**	2-(4-hydroxybenzylidene)-cyanoacetamide	
**2**	2-(4-ethoxybenzylidene)-cyanoacetamide	
**3**	2-(3-methoxybenzylidene)-cyanoacetamide	
**4**	2-(4-nitrobenzylidene)-cyanoacetamide	
**5**	2-(3,4-methylenedioxybenzylidene)-cyanoacetamide	
**6**	Tyrphostin AG99	

### Chemicals and reagents

All chemicals and reagents utilized in this study were of research grade, sourced from Merck in Germany, and were employed without further purification. Prior to their use, solvents underwent purification through the standard distillation method. Throughout the experimental procedures, deionized water that had been filtered through a Millipore filter was employed. Melting points (mp) were ascertained using an electrothermal melting point apparatus, with the recorded values being unadjusted. The advancement of the reaction was tracked through thin-layer chromatography (TLC) conducted on Kieselgel GF254 plates, employing a mixture of n-hexane and ethyl acetate in a 3:1 ratio. Subsequently, the plates were dried, and the spots were detected using UV lamps. Fourier-transform infrared (FT-IR) spectra were acquired utilizing a KBr matrix and an FT-IR spectrophotometer (Model-8900, Shimadzu, Japan) within the 4000–200 cm⁻¹ wavelength range. The 1H—NMR (nuclear magnetic resonance) spectra were recorded with a Bruker DPX-400 (400 MHz NMR) spectrometer from Switzerland, employing CDCl_3_, CD_3_OD, and DMSO‑d_6_ as solvents and Tetramethylsilane (TMS) as an internal standard. Chemical shifts were reported in δ units (ppm), and J values were indicated in Hz. The reactions were carried out using a commercially available LG microwave oven (MB-3947C, China) with a maximum power output of 800 W, operating at 2450 MHz.

### Synthesis of unsaturated cyanoacetamide derivatives (**1 – 5**)

2-(4-hydroxybenzylidene)-cyanoacetamide (**1**) was synthesized from 4-hydroxybenzaldehyde, 2-cyanoacetamide, and ammonium acetate using microwave conditions. 4-Hydroxybenzaldehyde (0.4885 g, 4 mmol) and 2-cyanoacetamide (0.33632 g, 4 mmol) were combined in a porcelain dish. Afterward, 10 mg of ammonium acetate was added and thoroughly mixed with the other components. The porcelain dish was then placed in the microwave oven and exposed to 160 W of microwave irradiation for 40 s. The reaction progress was tracked via TLC (n-hexane: ethyl acetate, 6:1). The resulting product was obtained as a reddish-black-brown solid. To refine the crude solid, it was recrystallized from a mixture of ethyl acetate and n-hexane, yielding compound **1** as a black-brown crystalline solid. The formation of the product was confirmed through FT-IR and 1H—NMR spectroscopy. Likewise, the characterization of the five unsaturated cyanoacetamide derivatives (**2**–**5**) detailed in the Supplementary Information is presented in Scheme 1 (refer to Table 1 and Figures S2 to S15 in the Supplementary Information).

### The antimicrobial activity

The in vitro assessment of antibacterial activity for the newly synthesized compounds (**1**–**5**) involved testing against four Gram-positive bacteria (*Bacillus subtilis* BTCC17, *Bacillus cereus* BTCC19, *Bacillus megaterium* BTCC18, *Staphylococcus aureus* ATCC6538) and two Gram-negative bacteria (*Salmonella paratyphi* ATCC11511 and *Salmonella typhi* AE14612). This evaluation adhered to the previously reported media preparation procedure [36]. Standard drug ampicillin was used in this study for evaluation antibacterial activity. The bacterial strains were exposed to a 200 µg/mL sample concentration in DMF, and ampicillin at the same concentration served as a reference. The inhibition zones were measured to record bacterial growth.

Furthermore, the in vitro assessment of the antibacterial properties of unsaturated cyanoacetamide derivatives (**1**–**5**) using the disk diffusion method was based on the presence or absence of inhibition zones' diameter (in mm). Initially, a 2% solution of the cyanoacetamide derivatives in DMF was prepared. Bacterial cultures were grown using Mueller-Hinton agar and broth media. Sterilized paper discs with a 4 mm diameter were placed in 70 mm petri plates, which had been autoclaved and dried at 150 °C in an electric oven. These paper discs were then loaded with the test compound(s) at a 100 µg/disc rate. A positive control was prepared using ampicillin at the same concentration as the compounds for all bacterial strains. The Petri plates containing the paper discs were incubated at 37 ± 1 °C for 48 h to facilitate the growth of the test organisms, followed by cooling for 4 h at a lower temperature (4 °C). The test chemicals diffused from the discs into the surrounding medium during this time. The extent of growth inhibition was calculated at 24-hour intervals over two days. Each experiment was conducted three times, and the mean of the triplicates was reported.

## Methodology

### Computational analysis

The computational calculations were performed using the Gaussian16 software [37]. The molecular geometries were optimized using the B3LYP/6–31G(d,p) level of theory. To analyse the electronic properties of the compounds, Frontier Molecular Orbital (FMO) calculations were conducted, which involved determining the energy distribution from the highest occupied molecular orbital (HOMO) to the lowest unoccupied molecular orbital (LUMO). Molecular electrostatic potential (MEP) maps were also generated. These calculations were carried out using the Gauss View 6 software [38]. In this study, various chemical descriptors were examined for each compound. These descriptors, including Energy gap (EGap), Ionization potential (IP), Electron affinity (EA), Chemical potential (µ), Electronegativity (χ), Hardness (η), Softness (σ), and Electrophilicity (ω), were calculated using the following equations [39], [40], [41]:

E_Gap_ (eV) = (E_LUMO_ − E_HOMO_); IP (eV) = −E_HOMO_; EA (eV) = −E_LUMO_;

χ (eV) = (IP + EA)/2; µ = −χ; η = (IP − EA)/2; σ = 1/η; ω = µ^2^/2η

The values of µ and η were used to derive the electrophilicity indexes. Additionally, to obtain potential energies and atomic charges, single point energy calculations and natural bond orbital (NBO) analysis were performed [42].

### Evaluation drug-likeness and admet properties

In this study, we utilized various free online tools such as AdmetSAR (http://lmmd.ecust.edu.cn/admetsar2/) and SwissADME (www.swissadme.ch) to predict the ADMET (Absorption, Distribution, Metabolism, Excretion, and Toxicity) properties, drug-likeness, and medicinal friendliness of α,β-unsaturated cyanoacetamide derivatives (**1**−**5**) [34], [35]. The structures (**1**–**5**) were drawn using ChemBioDraw Ultra 14.0 and converted to a canonical simplified molecular input line entry system (SMILES) to collect MDL Molfile format.

### Preparation of ligands

The three-dimensional (3D) structure data files (SDF) of the derivatives and reference drugs were obtained in SDF format from the PubChem database. GaussView 6 software was used to draw all the compounds, and their structures were optimized at B3LYP/6–31G(d,p) using the Gaussian 16 package. To prepare the structures of all the selected ligands for docking, energy minimization (EM) was performed. Then, the freely available program OpenBabel plugin of PyRx 0.8 software (available at https://pyrx.sourceforge.io/) was utilized to convert the structures to the PDBQT format [43]. This allowed for the effective preparation of the ligands for docking.

### Preparation of target protein

To identify the molecular docking software was utilized to search for a suitable protein that could potentially bind to the ligands, molecular docking software was utilized, and the required proteins were obtained from the RSCB Protein Data Bank tool (https://www.rcsb.org/) [44]. In this study, a total of eight proteins were selected, including *S. Paratyphi* (PDB: 6SE1), *Vibrio Cholera* (PDB: 5GVZ), *B. cereus* (5N1J), *B. Megaterium* (PDB: 4NZZ), *B. Subtilis* (PDB: 7FEQ), *S. Aureus* (PDB: 5MM8), *Plasmodium Falciparam* (PDB: 5XVU) and *S. typhimurium* (1TM2). The optimization of the protein structures was performed using Chimera version 1.16 [45]. This allowed for the effective utilization of the selected proteins in molecular docking studies. For the molecular docking and MD simulation, additional steps were taken to optimize each protein. These steps involved removing water molecules and ligands, as well as conducting energy minimization on the macromolecule. The protonation state of histidine was used, and the standard residue was set to the default AMBER ff14SB for both ligands and proteins. The Gasteiger method was used to calculate the charge for other residues of each protein.

### Protein-ligand docking

AutoDock Vina [46] software was employed for protein-ligand molecular docking, utilizing the ligand structures and target proteins. To ensure comprehensive coverage, a grid box was positioned to encompass the entire protein at its centre during the ligand docking process. Throughout the docking experiment, both the proteins and ligands remained in a stable state. The validation of these protein-ligand interactions was carried out through a re-docking procedure. Additionally, for the visualization of receptor-ligand binding modes, tools such as Chimera v1.16 [45], PyMol [47], and BIOVIA Discovery Studio visualizer were utilized.

### 3D protein structure refinement and validation

The refinement of the 3D model was carried out using the Galaxy Refine server [48], and the quality of the prepared protein structure was evaluated using various online tools. Validation of the target protein was conducted using the Procheck [50] and ProSA-web [49] servers. ProSA-web generated a Z-score to assess the overall quality of the drug. At the same time, the Procheck server was used to analyze the Ramachandran plot, providing insights into the overall quality.

### Molecular dynamics simulation

Molecular dynamics (MD) simulations were executed using the GROMACS version 2021.6 package [51] in conjunction with the AMBER99SB force field [52], which provides a comprehensive representation of atomic interactions. In contrast to less computationally intensive docking methods, MD simulation is a robust computational technique. It boasts exceptional precision and affords valuable insights into the behaviour of intricate systems that may be beyond the reach of experimental approaches [53]. In this research, we employed molecular dynamics (MD) simulations to investigate the docked complexes formed by PDB: 5MM8 and compound 5 within the active binding site of the modelled protein at various temperatures, including 300, 305, 310, and 320 K. The topology parameters for the proteins within the system were generated through the Galaxy European Server [54], a widely recognized platform for molecular modelling and simulation. The system was then solvated with SPC water molecules, housed within a triclinic box, and balanced with sodium and chloride ions to attain standard salt concentrations while neutralizing the system [55].

To ensure the stability of the system, we initiated an equilibration process involving a position-restrained dynamics simulation (NVT) at 300 K, lasting for 3000 ps, and implemented through the leapfrog algorithm [12,56]. Following this equilibration phase, the entire system underwent a production run for an additional 3000 ps under constant temperature and pressure conditions. The utilization of MD simulations in this study facilitated the analysis of the behaviour exhibited by the protein-ligand complexes formed during docking. This insight enhances our comprehension of the underlying physical principles governing the structural functionality of biological macromolecules. We employed VMD [57], PyMol [47], and GROMACS programs [51] to visualize and analyse the MD trajectories generated. All MD simulations were conducted for a duration of 50 ns, with temperature settings at 300 K, 305 K, 310 K, and 320 K. GROMACS utilities were used to perform statistical analyses, including root-mean-square-deviation (RMSD), radius-of-gyration (Rg), and root-mean-square fluctuation (RMSF), accomplished through 'gmx rmsd,' 'gmx gyrate,' and 'gmx rmsf,' respectively. Hydrogen bond analysis was conducted using the 'gmx hbond' utility in GROMACS, while temperature and potential energy were determined through the 'gmx energy' tool. Furthermore, principal component analysis (PCA) was employed to assess the stability of protein complexes, proving to be highly advantageous [58], [59], [60]. The PCA for the MD trajectories obtained was carried out using the Bio3D package facilitated by the Galaxy European server [61], [62], [63].

### Results and discussion

The study aimed to synthesize five α,β-unsaturated cyanoacetamide compounds while adhering to green chemistry principles. This was achieved using aromatic aldehydes and active methylene compounds, such as 2-cyanoacetamide, with NH_4_OAc as a catalyst in a modified Knoevenagel reaction. The reactions were conducted with microwave irradiation, aligning with green chemistry principles, and utilizing ammonium acetate as the catalyst. The present study focused on the investigation of α,β-unsaturated 2-cyanoacetamide derivatives (**1**−**5**), as illustrated in Scheme 1. Detailed information regarding these compounds can be found in Table 1 and Figures S1 to S15 in the Supplementary Information (SI).

### Characterization of unsaturated 2-cyanoacetamide derivatives (**1** to **5**)

The structural confirmation of 2-(4-hydroxybenzylidene)-cyanoacetamide (**1**) was accomplished by analysing its ^1^H NMR spectrum, as detailed in Figures S1−S15 in the supplementary information. In this spectrum, a singlet signal at δ 8.09 ppm was assigned to the =CH (benzylidene proton). In contrast, two doublet signals at δ 7.90 and δ 6.90, each with a *J* value of 8.8 Hz, were attributed to the aromatic H-2, H-6, and H-3, H-5, respectively. Additionally, a two-proton singlet signal at δ 4.84 ppm was assigned to the -NH_2_ proton and a one-proton singlet signal at δ 3.30 ppm corresponded to the -OH proton. Furthermore, the FT-IR spectrum of compound **1** (as illustrated in Figure S1 in the supplementary information) revealed characteristic absorption bands, including stretching bands at 3400.15 cm^−1^ and 3313.75 cm^−1^, signifying the presence of the -NH2 group. An absorption band at 3367.71 cm^−1^ indicated the existence of the -OH group. Moreover, there was an absorption stretching band at 3197.98 cm^−1^, which was attributed to the =C—H bond, and an absorption band at 2229.71 cm^−1^ corresponding to the C≡N bond. Additionally, an absorption band at 1685.79 cm^−1^ denoted C=O stretching. The spectrum also exhibited characteristic peaks for C=C olefinic (1600.92 cm^−1^) and aromatic C=C (1577.91 cm^−1^) functionalities.

*2-(4-hydroxybenzylidene)-cyanoacetamide (****1****):* The synthesis of 2-(4-hydroxybenzylidene)-cyanoacetamide (**1**) yielded a black-brown crystalline solid, providing a yield of 98.6%. Its molecular formula was determined to be C_10_H_8_N_2_O_2_, and it exhibited a melting point range between 114 and 116 °C. Elemental analysis yielded the following results: Anal. Calcd. for C_10_H_8_N_2_O_2_. Anal. Calcd for C_10_H_8_N_2_O_2_: C, 63.83; H, 4.29; N, 14.89. Found: C, 58.25; H, 4.89; N, 13.59. FT-IR (KBr) ν_max_(cm^−1^): 3400.15, 3313.71 (N—H), 3367.71 (b, OH), 3197.98(C—H), 2229.71 (s, C≡N), 1685.79 (C = O, amide), 1600.00 (s, C=C), 1573.91 (s, C=C, Ph). ^1^H NMR (400 MHz, CDOD) *δ*ppm: 8.09 (s, 1H, CH), 7.90 (d, 2H, *J* = 8.8 Hz, H-2, H-6, Ph), 6.90 (d, 2H, *J* = 8.8 Hz, H-3, H-5, Ph), 4.84 (s, 2H, -NH_2_), 3.30 (s, OH).

*2-(4-ethoxybenzylidene)-cyanoacetamide (****2****):* The product (**2**), known as 2-(4-ethoxybenzylidene)-cyanoacetamide, was acquired in the form of a white crystalline solid, yielding 94.0%. Its molecular formula is C_12_H_12_N_2_O_2_, and it exhibits a melting point within the range of 156–158 °C. Elemental analysis yielded the following results: Elemental analysis yielded the following results: Anal. Calcd for C_12_H_12_N_2_O_2_: C, 66.65; H, 5.59; N, 12.96. Found: C, 61.00; H, 6.83; N, 11.86. FT-IR (KBr) ν_max_(cm^−1^): 3441.01, 3394.72 (N—H), 3159.40 (C—H), 2210.42 (s, C≡N), 1705.07 (s, C=O, amide), 1590.00 (s, C=C), 1508.33 (s, C=C, Ph). ^1^H NMR (400 MHz, CDCl_3_)*δ* ppm: 8.13 (s, 1H, C—H), 7.94 (d, 2H, H-2, H-6, *J* = 8.8 Hz, Ph), 7.01 (d, 2H, H-3, H-5, *J* = 8.8 Hz, pH), 4.77 (s, 2H, -NH_2_), 4.12 (q, 2H, OCH_2_), 1.42 (t, 3H, *J* = 7.2 Hz, CH_3_).

*2-(3-methoxybenzylidene)-cyanoacetamide (3):* Product (**3**), 2-(3-methoxybenzylidene)-cyanoacetamide, was obtained as an off-white crystalline solid with a yield of 76.1%. Its molecular formula is C_11_H_10_N_2_O_2_, and it has a melting point range of 49–51 °C. Elemental analysis yielded the following results: Anal. Calcd for C_11_H_10_N_2_O_2_:C, 65.34; H, 4.98; N, 13.85. Found: C, 59.99; H, 5.49; N, 12.70. FT-IR (KBr) ν_max_(cm^−1^): 3394.72, 3313.71 (N—H), 3190.26 (C—H), 2222.00 (s, C≡N), 1716.65 (s, C=O, amide), 1631.78, 1593.20 (s, C=C). ^1^H NMR(400 MHz, CDCl_3_) *δ* ppm: 8.16 (s, 1H, CH), 7.57 (s, 1H, H-2), 7.48 (d, 1H, H-6, *J* = 8.0 Hz, Ph), 7.42 (t, 1H, H-5, *J* = 8.0), 7.12 (d, 1H, H-4, *J* = 8.0 Hz, Ph), 4.84 (s, 2H, -NH2), 3.84 (s, 3H, OCH3).

*2-(4-nitrobenzylidene)-cyanoacetamide (****4****):* 2-(4-nitrobenzylidene)-cyanoacetamide (**4**) was obtained as a deep brown crystalline solid in 99.1% yield by recrystallization from a mixture of ethyl acetate and n-hexane. Its molecular formula is C_10_H_7_N_3_O_3_, and its melting point is 208–210 °C. Anal. Calcd for C_10_H_7_N_3_O_3_: C, 66.65; H, 5.59; N, 12.96. Found: 51.07; H, 3.86; N, 17.87. FT-IR (KBr) ν_max_(cm^−1^): 3441.01, 3344.51 (N—H), 3197.98 (C—H), 2225.00 (s, C≡N), 1697.36 (C=O, amide), 1600.90 (s, C=C, Ph). ^1^H NMR (400 MHz, CDCl_3_) *δ* ppm: 8.36 (d, 1H, *J* = 8.8 Hz, H-2, Ph), 8.29 (s, 1H, CH), 8.21 (d, 1H, H-3, *J* = 8.8 Hz, Ph), 8.15 (d, 1H, H-5, *J* = 8.84 Hz, Ph), 7.65 (d, 1H, H-6, *J* = 8.8 Hz, Ph), 4.85 (s, 2H, -NH_2_).

*2-(3,4-methylenedioxybenzylidene)-cyanoacetamide (****5****):* Product (**5**), 2-(3,4-methylene dioxybenzylidene)-cyanoacetamide, was obtained as a greenish yellow crystalline solid in 97.3% yield, M.F. C_11_H_8_N_2_O_3_, with a melting point of 178–180 °C. Anal. Calcd for C_11_H_8_N_2_O_3_: C, 61.11; H, 3.73; N, 12.96. Found: C, 56.41; H, 4.30; N, 11.96. FT-IR (KBr) ν_max_ (cm^−1^): 3475.73, 3375.77 (N—H), 3132.40 (C—H), 2214.28 (s, C≡N), 1716.65 (C=O, amide), 1577.77 (s, C=C). ^1^H NMR (400 MHz, CDCl_3_) δ ppm: 8.08 (s, 1H, CH), 7.65 (s, 1H, H-2, Ph), 7.43 (d, 1H, H-6, *J* = 8.0 Hz, Ph), 6.97 (d, 1H, H-5, *J* = 8.0 Hz, Ph), 6.09 (s, 2H, OCH_2_O), 4.84 (s, 2H, -NH_2_).

### In vitro antibacterial activity

**Fig. 1:**
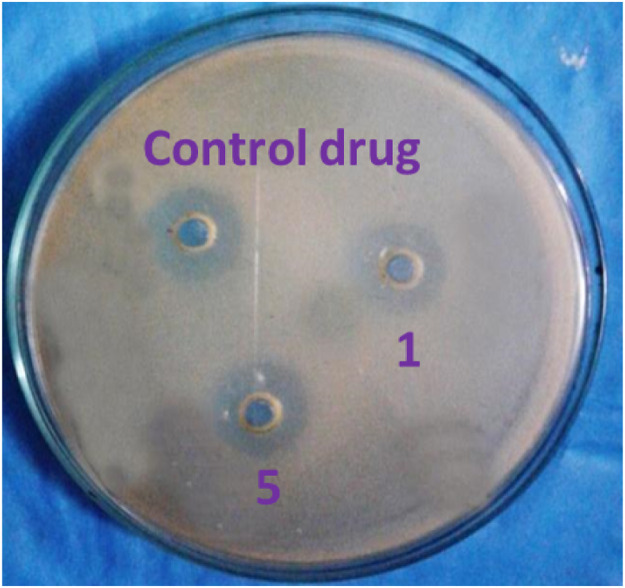
Zone of inhibition observed against *S. aureus* by two test compounds (**1** and **5)** and control drug, ampicillin.

The newly synthesized unsaturated 2-cyanoacetamide derivatives (**1**–**5**) were assessed for their in vitro antibacterial properties against six pathogenic human bacteria using both the disk diffusion and agar dilution assay methods. The results can be found in Table 2 and Fig. 1. The disk diffusion data revealed that unsaturated 2-cyanoacetamide derivatives (**1**–**5**) demonstrated a range of antibacterial effectiveness against the targeted bacterial strains, with mean zones of inhibition varying from moderate (11.1 ± 0.77 mm) to high (19.8 ± 0.83 mm) at a concentration of 200 µg/mL.

**Table 2:** Antimicrobial activity of the synthesized compounds (**1−5**) against selected bacterial strains (MIC in 200 µg/mL)^a^.

Drug	bacterial strains (zone of inhibition in mm)					
	*B. subtilis*	*B. cereus*	*B. megaterium*	*S. aureus*	*S. paratyphi*	*S. typhi*
1	11.1 ± 0.77	NI	NI	17.3 ± 0.44	6.1 ± 0.56	12.5 ± 0.56
2	NI	NI	NI	NI	6.1 ± 0.50	6.2 ± 0.50
3	18.2 ± 0.50	11.5 ± 0.50	13.1 ± 0.50	NI	18.5 ± 0.50	13.5 ± 0.50
4	NI	14.9 ± 0.50	NI	NI	8.7 ± 0.50	9.2 ± 0.50
5	13.9 ± 0.42	NI	NI	19.8 ± 0.83	NI	NI
Ampicillin	19.1 ± 0.50	18.0 ± 0.50	16.0 ± 0.50	21.0 ± 0.45	20.0 ± 0.75	18.0 ± 0.85

a Data are presented as Mean ± standard deviation; “NI” indicates no inhibition.

All the derivatives exhibited notable activity against *S. aureus* for compound **5**, displaying mean inhibitions ranging from 11.3 ± 0.44 mm to 19.8 ± 0.83 mm at 200 µg/mL. This aligns well with the positive control, ampicillin, which displayed a 21.0 ± 0.45 mm inhibition zone at the same concentration. Furthermore, the data in Table 2 highlights that compound **5** demonstrated superior antibacterial activity against *B. cereus, B. megaterium, S. aureus*, and *S. typhi* compared to the other compounds (**1**–**4**). It is worth noting that in all instances, compound **5** exhibited greater effectiveness against *S. aureus* when compared to compounds (**1**–**4**), consistent with the performance of the standard drug. The inhibition zone figures represent the mean values from three separate and independent experiments.

### Quantum chemical analysis

**Fig. 2:**
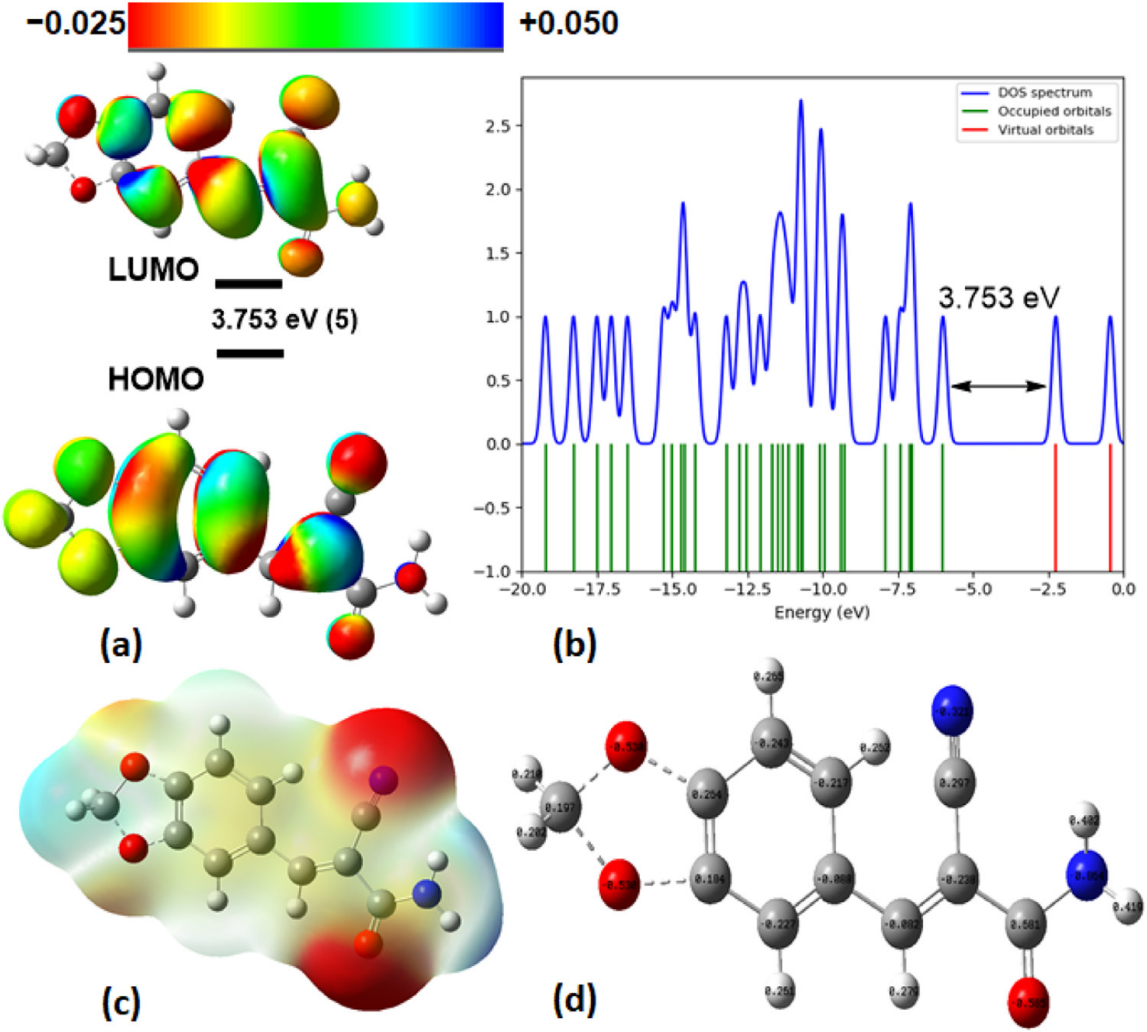
(a) Molecular orbitals of isodensity surfaces (0.02 electrons Bohr−3 surface) (red = electron-rich, blue = electron-deficient) of HOMO and LUMO; (b) DOS plot and HOMO-LUMO energy gap; (c) Maps of electrostatic potential (0.02 electrons Bohr−3 surface) (red = electron-rich, blue = electron-deficient); (d) NBO charges structures for the compound, **5**.

The analysis of cyanoamide derivatives (**1**–**5**) through Frontier Molecular Orbital (FMO) analysis has provided valuable insights into their chemical reactivity and stability. Specifically, the HOMO-LUMO gap (Egap) is crucial in determining various essential properties, including chemical reactivity, hardness, softness, chemical potential, and electrophilic index [64]. A wider Egap indicates higher stability and lower reactivity, while a narrower gap signifies softness, corresponding to increased reactivity and decreased stability. Apart from Egap, the energy levels of HOMO and LUMO are also significant in understanding these compounds' electron donor and acceptor characteristics. For example, compounds with a narrow energy gap are described as "soft" (σ), indicating high chemical reactivity and low stability, while those with a wider gap are referred to as "hard" (η). In this study, we computed the ionization potential (IP), electron affinity (EA), electronegativity (χ), chemical potential (µ), global hardness (η), softness (σ), electrophilicity (ω), and dipole moment using the B3LYP/6–31G(d,p) method. The results of these calculations are presented in Table 3 and Fig. 2a-b (see Figures S16 – S19 in the SI).

**Table 3:** MO of **t**he HOMO and LUMO energies, IP, EA, electronegativity (χ), chemical potential (µ), global hardness (η), softness (σ), electrophilicity (ω), and dipole moment (Debye) of all compounds (**1**–**5**) at 298.15 K^a^.

Ligand	E_LUMO_ (eV)	E_HOMO_ (eV)	E_gap_ (eV)	IP (eV)	EA (eV)	χ (eV)	µ (eV)	η (eV)	σ (eV)	ω (eV)	dipole (D)
1	−6.252	−2.188	4.064	6.252	2.188	4.220	−4.220	2.032	0.492	2.428	2.999
2	−6.114	−2.117	3.997	6.114	2.117	4.115	−4.116	1.998	0.500	2.469	3.608
3	−6.341	−2.305	4.036	6.341	2.305	4.323	−4.323	2.018	0.495	2.445	3.158
4	−7.378	−3.335	4.043	7.378	3.335	5.356	−5.356	2.021	0.494	2.441	3.771
5	−5.997	−2.244	3.753	5.997	2.244	4.110	−4.121	1.886	0.530	2.616	2.927
AG99	−6.077	−2.181	3.896	6.007	2.181	4.094	−4.129	1.913	0.522	2.579	3.906

a Calculated by: HOMO energy (E_LUMO_),LUMO energy (E_LUMO_), Energy gap (E_gap_)= E_LUMO_ − E_HOMO_, Ionization potential (IP) = −E_HOMO_, Electron affinity (EA) = −E_LUMO_, Electronegativity (χ) = (IP + EA)/2, Chemical potential (µ) = −χ, Hardness (η) = (IP − EA)/2, Softness (σ) = 1/η, Electrophilicity (ω) = µ2/2η.

Table 3 reveals that among all the compounds, compound **5** exhibited the largest HOMO-LUMO gap value (3.753 eV), highest hardness (1.886 eV), and lowest softness (0.530 eV). This can be attributed to its 3,4-methylenedioxybenzylidene ring, setting it apart from the other compounds. When comparing the HOMO-LUMO gaps of the reference compound AG99 and compound **5**, they display similar characteristics, but AG99 has a slightly higher gap than compound **5**, suggesting better stability and reactivity. Furthermore, the reaction involving compound **5** has led to a reduced HOMO-LUMO gap and a lower dipole moment (2.927 D), indicative of improved biological activities. The energy gap values decrease as follows: **5** (3.753 eV) > AG99 (3.896 eV) > **2** (3.997 eV) > **3** (4.036 eV) > **4** (4.043 eV) > **1** (4.064 eV). The higher global softness of compound **5** aligns well with the results obtained for antibacterial activities (Table 3). Similarly, compound AG99 exhibits stronger reactivity regarding the electronic chemical potential (µ) than compound **5**. This parameter can be used to gauge the reactivity of molecules, with reactivity increasing as chemical potentials decrease. Additionally, the electrophilicity index (ω) parameter indicates that compound **5** possesses a higher electrophilicity index (2.616 eV) than all the synthesized compounds, implying its ability to accept electrons [65]. Molecular docking with a suitable protein may offer further insights into these potential activities.

Fig. 2c displays Molecular Electrostatic Potential (MEP) maps that reveal likely reactivity regions in the molecule. Electrophilic sites (blue) accept electrons, nucleophilic sites (red) donate electrons, and partial nucleophilic regions are shown in yellow. The color-coded electrostatic potential signifies negative potential (red, orange, yellow) over electronegative atoms like oxygen and cyan for electrophilic reactivity, and positive potential (blue) over hydrogen atoms for nucleophilic reactivity. Green regions represent zero potential. A Natural Bond Orbital (NBO) [66] analysis was performed to understand molecule stability through atomic bonds and charges. It investigated both intermolecular and intramolecular interactions, highlighting significant changes in charges on key atoms in Figs. 2d and S16 to S19 in the SI. Intramolecular charge transfers were also explored.

### Molecular docking

**Fig. 3:**
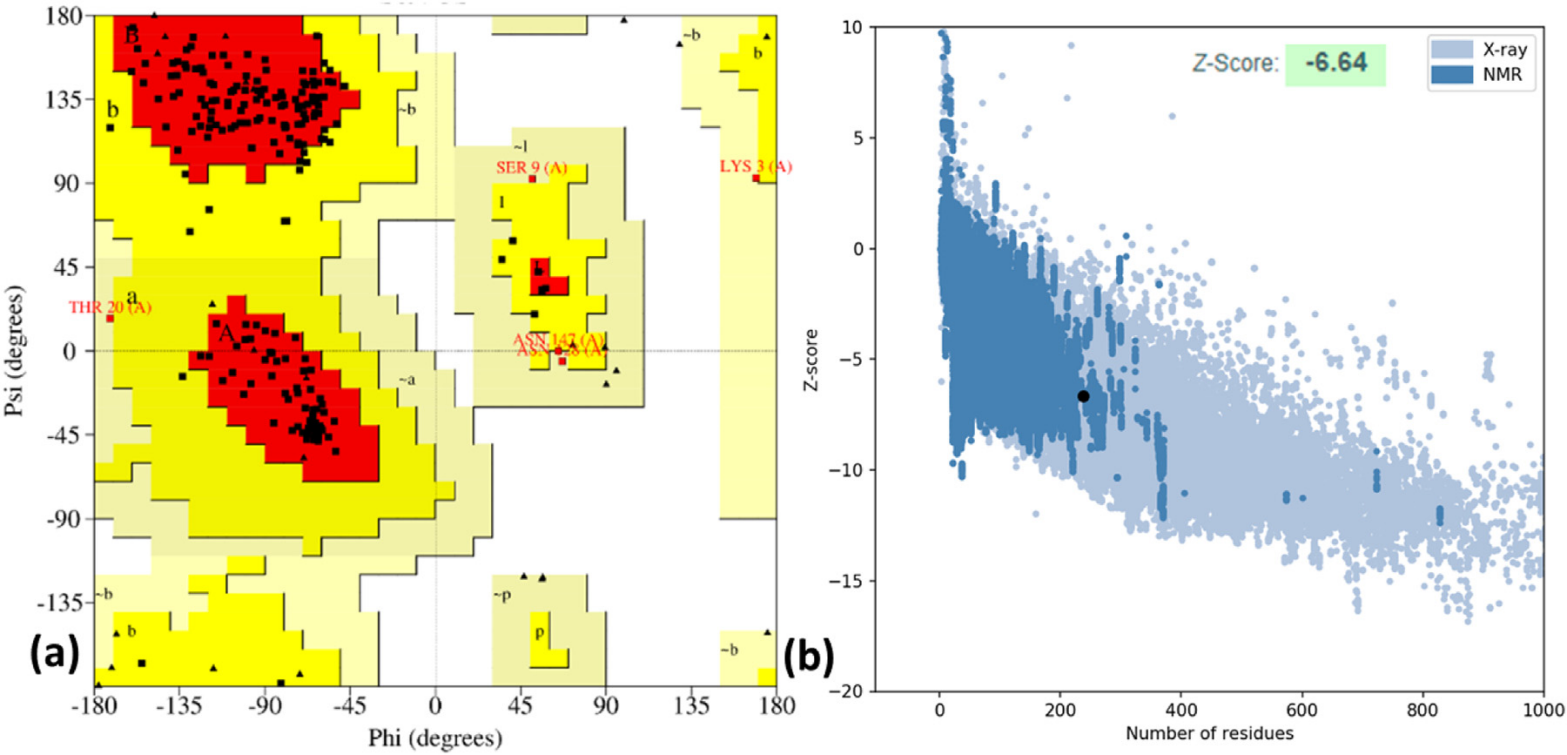
Validation and quality evaluation of protein (PDB: 5MM8) by using (a) Ramachandran Plot analysis by Procheck, and (b) Z-score predicted by the ProSA-web server.

The Ramachandran plot for PDB: 5MM8 was generated using the Procheck server to examine alterations in the protein structure following the preparation process. The resulting Ramachandran plot analysis revealed that 93.4% of residues occupy favored regions, as depicted in Fig. 3a. Z-scores, obtained from the ProSA-web server, were used to evaluate all chains in PDB: 5MM8 based on whether they were determined through X-ray crystallography (light blue) or NMR spectroscopy (dark blue) concerning their length. The overall model quality, represented by the z-score of PDB: 5MM8 (−6.64), is highlighted with a black dot in Fig. 3b. Consequently, we chose the target protein (PDB: 5MM8) for further analysis in this study, as illustrated in Fig. 3.

**Fig. 4:**
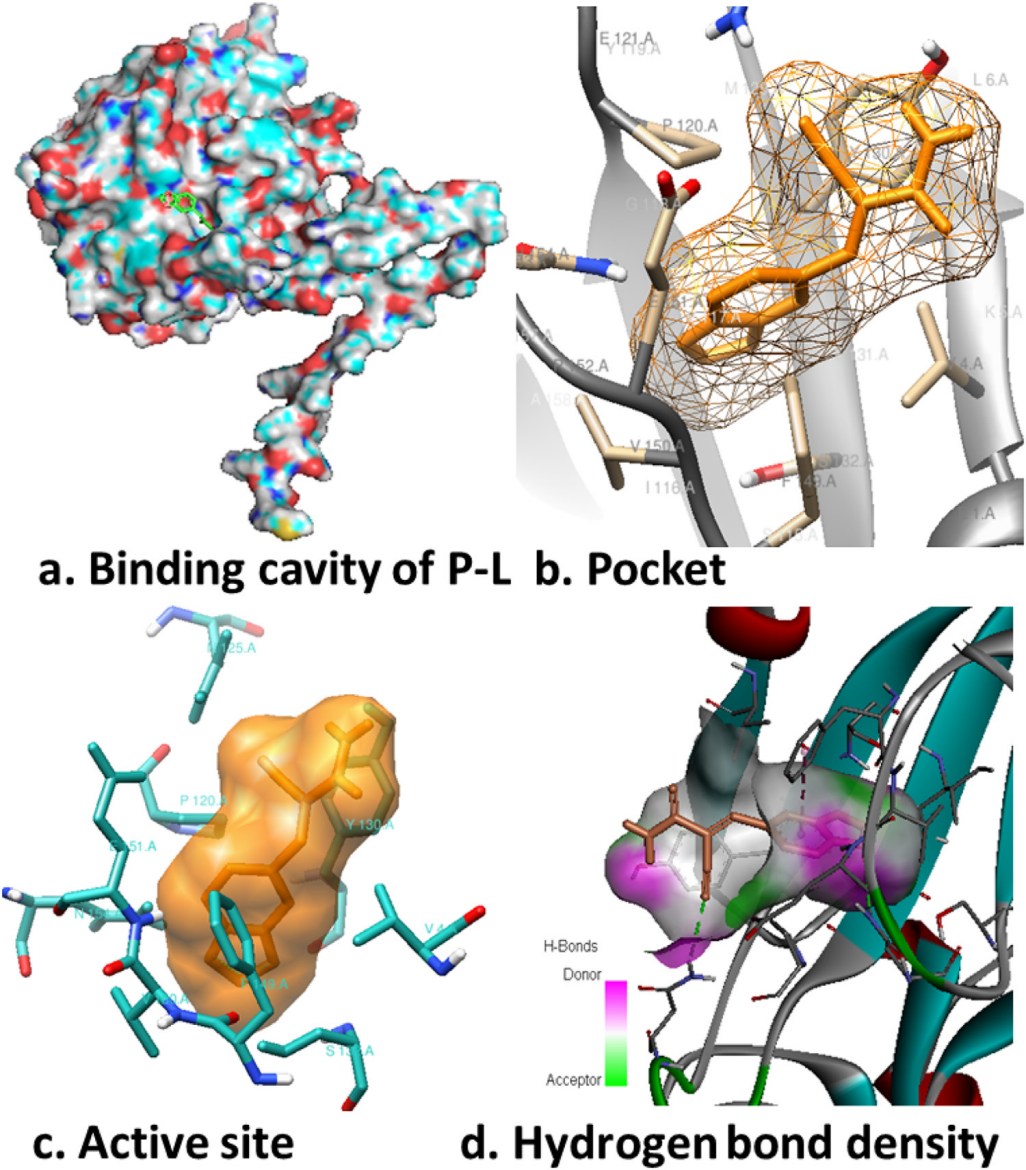
Molecular docking results: (a) binding cavity of protein-ligand pocket; (b) hydrogen bonding; (c) protein-ligand interaction in 2D diagram; (d) hydrogen bonding in solid state, for compound **5** in 5MM8.

Molecular docking allows for the analysis and prediction of plausible binding configurations and interactions between compounds and a protein's active site. This method provides valuable insights into how compounds behave and interact with a protein's active site residues and their subsequent impact on cellular processes. Our study conducted molecular docking experiments involving five cyanoacetamides (**1–5)** with seven antibacterial proteins (PDB: 5MM8, 5N1J, 4NZZ, 7FEQ, 6SE1, 5GVZ, and 1TM2). The findings in Table 3 show that *staphylococcus aureus* (PDB: 5MM8) displayed the highest binding affinity among the bacterial proteins when interacting with compound **5**, with a binding energy of −7.7 kcal mol^−1^, slightly surpassing the reference drug AG99 (−7.6 kcal mol^−1^). Compounds **1**–**5** exhibited higher binding affinities with *bacillus subtilis* (PDB: 7FEQ), when compared to the reference drug (−6.0 kcal mol^−1^). The molecular docking study revealed that compounds **5** exhibited strong binding affinities with all proteins, particularly with proteins 4NZZ and 5MM8 (refer to Table 4 and Figs. 4 and S20-S25 in the SI).

**Table 4:** Molecular docking simulation results for the synthesized compounds (**1−5**) against seven targets.

Ligand	Binding affinity (kcal mol^−1^)						
	*S. aureus* (5MM8)	*B. cereus* (5N1J)	*B. megaterium* (4NZZ)	*B. subtilis* (7FEQ)	*S. paratyphi* (6SE1)	*V. cholera* (5GVZ)	*S. typhi* (1TM2)
1	−6.8	−6.3	−7.0	−6.1	−5.9	−5.3	−5.6
2	−6.5	−6.3	−7.0	−6.3	−5.9	−5.3	−5.6
3	−6.4	−6.5	−7.2	−6.3	−6.1	−5.3	−5.3
4	−7.3	−6.9	−7.3	−6.5	−5.7	−5.7	−6.2
5	−7.7	−7.5	−7.6	−6.9	−6.4	−5.7	−6.1
AG99	−7.6	−6.6	−7.2	−6.0	−6.1	−5.6	−5.8

The interactions at the binding site between the docked protein, its ligand, and their corresponding derivatives (**1**–**5**) are detailed in Tables 4–5 and Supplementary Information (SI) Table S6. In the case of seven targets, it was observed that the synthesized organic compounds (**1**–**5**) exhibited lower binding affinity with compound **5** when compared to AG99, as depicted in Table 4. The analysis of chemical bonds formed between the ligand and the protein at the active site provided insights into how the ligand impacts disease-causing pathogens. The bond length and residue numbers for various types of bonds, such as ligand within the protein pocket, hydrogen bonding, ligand-protein interactions in 2D diagrams, and solid-phase hydrogen bonding with compound **5** across seven targets, are presented in Fig. 4 and are available in detail in Tables 4,5 and Figures S20 to S25 within the SI.

**Table 5:** Protein and ligand interacting amino acid residues of compound **5** against four targets.

Target (PDB ID)	interacting residues	distance	type of interaction	2D diagram of interaction
5MM8	ILE A:140 VAL A:65 LYS A:229 ALA A:233	5.18 2.33 2.59 2.20	π-Alkyl Conventional HB Carbon HB Carbon HB	
5NIJ	TRP A:485 ARG A:490 GLY A:491 TYR A:190	5.30 2.07 2.32 2.22	π-Alkyl Conventional HB Conventional HB Conventional HB	
4NZZ	LEU A:168 TRP A:147 TYR A:144	4.59 5.07 2.33	π-Alkyl π-Alkyl Conventional HB	
7FEQ	GLY H:32 GLN H:46 TYR H:20 MET H:30 ILE H:29	2.55 2.46 2.69 2.98 2.87	Conventional HB Conventional HB Conventional HB Conventional HB Conventional HB	

Protein-ligand interactions via amino acid residues: compound **5** with 5MM8, 5N1J, 4NZZ, and 7FEQ are given in Tables 4,5 and Figs. 4 (S20-S25 in the SI). Several interactions were observed in the protein-ligand interaction analysis of compound **5** with 5MM8, including two carbon hydrogen interactions with LYS A:229 and ALA A:233, one Pi-Alkyl interaction, and one interaction classified as a conventional hydrogen bond involving the VAL A:65 residue. Remarkably, it was evident that compound **5** displayed a strong binding affinity for 5N1J, with a total of four interactions. These encompassed three conventional hydrogen bonds involving the ARG A:490, GLY A:491, and TYR A:190 residues, along with a Pi-Alkyl interaction related to TRP A:485. When considering the interaction between compound **5** and protein 4NZZ, it is essential to note the presence of three distinct interactions. Two of these interactions are hydrophobic, specifically Pi-Alkyl interactions involving the LEU A:168 and TRP A:144 residues. Furthermore, one conventional hydrogen bond is observed at the TYR A:144 residue. In the context of the interaction between compound **5** and protein 7FEQ, it is significant to highlight three distinct interactions. Among these, four interactions take the form of conventional hydrogen bonds involving GLY H:32, GLN H:46, TYR H:20, and MET H:30 residues. Furthermore, one interaction is categorized as a carbon-hydrogen bond associated with the ILE H:29 residue.

Similarly, the analysis of the protein-ligand interaction between compound **5** and 6SE1 revealed several interactions as shown in Table S7 and Figure S23 in the SI. These interactions comprised two conventional hydrogen interactions with the LYS A:421 and SER A:527 residues, one Pi-Alkyl interaction with HIS A:422, and one interaction categorized as a Carbon Hydrogen Bond involving the GLU A:492 residue. Compound **5** exhibited a strong binding affinity for 5GVZ, engaging in five interactions (see Figure S24 in the SI). These encompassed three conventional hydrogen bonds with the GLN A:1, GLN A:57, and ARG A:60 residues, a Carbon Hydrogen Bond with GLU A:58, and a Pi-Alkyl interaction involving LYS A:4. Moreover, numerous noteworthy interactions were observed when examining protein-ligand interactions involving compound **5** and 1TM2 (Figure S25 in the S1). These included two carbon hydrogen interactions with SER A:161 and SER A:160, one Pi-Alkyl interaction with PRO A:68, and a distinctive Pi-Oi T-shaped interaction with TYR A:194. Furthermore, three interactions were classified as conventional hydrogen bonds involving the residues THR A:163, VAL A:93, and ASN A:195. The stability of the protein (5MM8) and ligand (5) complex was assessed through an additional molecular dynamics (MD) simulation study.

### Drug-Likeness and admet predictions

To determine the potential suitability of cyanoamide derivatives (**1**–**5**) for oral administration, it is essential to evaluate their physicochemical properties according to the Lipinski and Veber guidelines. A compound is classified as a drug candidate only if it meets the criteria for drug-likeness and passes *in silico* ADMET (Absorption, Distribution, Metabolism, Excretion, and Toxicity) predictions. Lipinski's Rule of Five [67] and Veber's rule [68] is a widely employed guideline for assessing the drug-like characteristics of compounds. This rule comprises a set of physicochemical parameters, including molecular weight (MW) <500 Da, number of H-bond donors <5, number of H-bond acceptors <10, lipophilicity (mLogP) value should be <4.15 for optimum balance, and molar refractivity (MR). Along with Lipinski's rule of five, other physicochemical properties,Veber rules add that nrotb must be less than 10 and TPSA must be less than 140 Å^2^ were determined using SwissADME, and cyanoamide derivatives (**1**–**5**) were passed all these properties. Medicinal chemistry (PAINS #alerts and Brenk #alerts) of structures was also predicted using SwissADME. The results of drug-likeness prediction of compound **5** with the highest negative binding affinity of more than −7.7 kcal/mol a represented in Table 4, and out of **5** synthesized cyanoamide derivatives (**1**–**5**). Drug-likeness and *in silico* ADMET profiling of compound **5**, which showed the highest negative binding affinity via molecular docking, showed satisfactory results with good drug-like properties and it also followed the ADMET requirements (see Table 6). Therefore, based on their physicochemical properties, these compounds show potential for further development as novel medications.

**Table 6:** Lipinski's rule of five and veber's rule for drug-likeness prediction of all compounds (**1**–**5**).

Ligand	MW	mLogP	nHBD	nHBA	MR	Lipinski's violations	Veber's violations	TPSA	nRot	PAINS #alerts	Brenk #alerts
Lipinsli*	≤500	≤5	≤5	≤10	–	–	–	–	–	–	–
Veber^⁎⁎^	–	–	–	–	–	–	–	≤140	≤10	–	–
1	188.18	0.25	2	3	50.83	0	0	87.11	2	0	2
2	216.24	0.87	1	3	60.10	0	0	76.11	4	0	2
3	202.21	0.54	1	3	85.30	0	0	76.11	3	0	2
4	217.18	−0.27	1	4	57.63	0	0	112.7	3	0	3
5	216.19	−0.01	1	4	54.87	0	0	85.34	2	0	2
AG99	204.18	−0.31	3	4	52.85	0	0	107.3	2	1	3

Evaluating the pharmacokinetic properties, including absorption, distribution, metabolism, excretion, and toxicity (ADMET), is essential for cost-effective drug development. We analysed the ADMET attributes of five active compounds using three freely available online tools: PreAdmet, admetSAR, and SwissADME. We assessed various characteristics like water solubility, oral bioavailability, blood-brain barrier permeability, and toxicity. Our primary focus was on seven ADMET parameters: human intestinal absorption (HIA), blood-brain barrier (BBB) penetration, plasma protein binding (PPB), CYP3A4 and CYP2C19 metabolism, lead-likeness, and synthetic accessibility (SA) scores. HIA, a pivotal aspect in drug development, categorizes absorption as high (70–100%), medium (20–70%), or low (0–20%). Compounds **1**–**5** displayed high HIA, suggesting their potential as oral drug candidates Plasma protein binding (PPB) can influence the drug's persistence in the body, where binding percentages exceeding 90% lead to a slower drug release, while percentages below 90% are deemed lower binding [69]. Compound **5** exhibited a PPB of 101, indicating strong protein binding, while AG99 had a PPB of 85.62, indicating weaker binding. *In silico* calculations revealed the involvement of CYP3A4 and CYP2C19, cytochrome P450 enzymes, in drug metabolism, inhibiting them and potentially increasing plasma levels and compound toxicity, as shown in Table 7. We introduced a novel method to assess the synthetic accessibility (SA) [70] score for drug-like molecules **1**–**5**. All five compounds had SA scores below 10, signifying their relative ease of synthesis. This SA score is valuable for virtual screening and predicting hERG activity (pIC50) for the identified molecules, as demonstrated in Table 7.

**Table 7:** *In silico* prediction of selected ADMET parameters for the cyanoamide derivatives (**1**−**5**).

aLigand	bHIA	bBBB	bPPB	bCYP3A4 inhibition	bCYP2C19 inhibition	bhERG_ pIC50	cSAcore
1	+ (0.996)	+ (0.725)	0.794	+(0.561)	- (0.943)	-(0.815)	2.03
2	+ (1.000)	+ (0.925)	0.939	- (0.799)	- (0.894)	- (0.392)	2.17
3	+ (1.000)	+ (0.850)	0.740	- (0.514)	- (0.946)	- (0.547)	2.18
4	+ (0.970)	+ (0.825)	0.977	+ (0.570)	- (0.750)	- (0.875)	2.18
5	+ (1.000)	+ (0.850)	1.013	+ (0.545)	- (0.578)	- (0.561)	2.50
AG99	+ (0.9914)	+ (0.550)	1.000	+(0.7822)	- (0.902)	-(0.815)	1.84

a HIA: Human Intestinal Absorption (%); BBB: Blood-Brain Barrier penetration; PPB: plasma protein binding; CYP3A4: Cytochrome P450 3A4; CYP2C19: Cytochrome P4502C19; hERG: human ether-a-go-go-related gene, hERG inhibition potential (pIC_50_),the potential risk for inhibitors ranges 5.5 − 6.

b The values are using admetSAR.

c Synthetic accessibility score values are using swissADME.

### Molecular dynamics simulation

The protein-ligand complex displayed a notably advantageous negative affinity and satisfied all the necessary criteria for potential drug candidacy. Based on the docking results, an MD simulation study was subsequently carried out using this protein-ligand complex in conjunction with 5MM8 for compound **5**. The MD simulation results were assessed to understand the structural stability, conformational variations, and residue fluctuations in the protein-ligand complex over a 50 ns simulation MD trajectory. A range of metrics, such as RMSD, RMSF, Rg values, as well as potential energies, temperature, hydrogen bonding, and principal component analysis (PCA), were employed to analyse the MD simulation results. Furthermore, the selected protein-ligand complex was also simulated in four different temperature conditions to determine its configurational changes that occurred in 300, 305, 310, and 320 K. Moreover, the chosen protein-ligand complex underwent simulations at four distinct temperature conditions (300 K, 305 K, 310 K, and 320 K) to investigate any configurational changes that may have occurred.

**Fig. 5:**
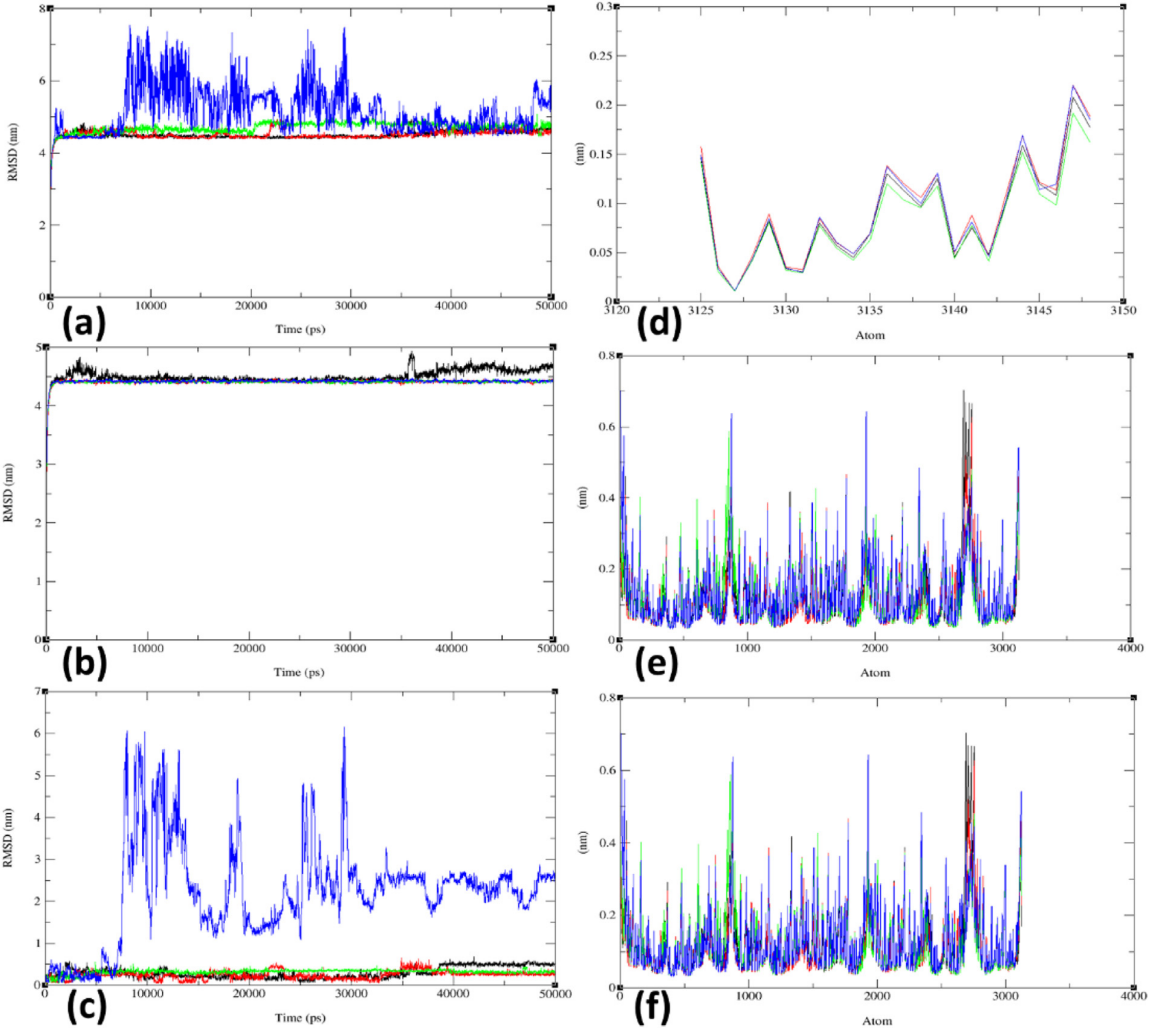
(a) RMSD progression for (a) combined ligand **5** under four different temperature conditions (300 K: black line, 305 K: red line, 310 K: green line, 320 K: blue line); (b) combined target protein (PDB: 5MM8); (c) combined docked complex involving the protein (PDB: 5MM8) and ligand **5**; (d) RMSF assessment for the combined ligand **5** under four different temperature conditions; (e) combined target protein (PDB:5MM8); and (f) combined target protein, PDB: 5MM8, and ligand **5** during the 50 ns MD simulation.

Throughout the simulation, the Root Mean Square Deviation (RMSD) values for (a) the combined ligand, 5, (b) the combined target protein, 5MM8, and (c) the combined docked complex between the protein and ligand were recorded under four distinct temperature conditions: 300 K, 305 K, 310 K, and 320 K during the 50 ns MD simulation. The RMSD plots, which were analysed for ligand **5** at various temperatures, are shown in Figs. 5a-c and Supplementary Information (SI) Figures S26-S28. In terms of RMSD analysis, ligand 5 consistently exhibited the most stable conformations, displaying minimal deviations in RMSD values, ranging from approximately 4.3 to 4.5 nm at 300 K, approximately 4.4 to 4.6 nm at 305 K, and around 4.6 to 4.8 nm at body temperature (310 K). Notably, the RMSD plot at 310 K showed increased scattering compared to the other three temperature conditions, as illustrated in Fig. 5a and Supplementary Information (SI) Figure S26. Conversely, simulations at 320 K resulted in the highest RMSD scattering, roughly 4.7 to 7.5 nm. In contrast, RMSD plots for the target protein (5MM8) at various temperatures displayed minimal RMSD deviations. These can be observed in Figs. 5b and Supplementary Information (SI) Figure S27. In the context of RMSD analysis during the simulation of the ligand-protein complex, the RMSD values at 300 K varied from approximately 0.2 to 0.5 nm, while at 305 K, they ranged from about 0.2 to 0.4 nm. At body temperature (310 K), the RMSD plot exhibited a stable scattering pattern, spanning from approximately 0.2 to 0.3 nm, which differed from the other three temperature conditions (see Figs. 5c and S28 in the SI). These results suggest that at 310 K, the binding between the ligand and protein is robust and enduring, enabling the complex to maintain its structural integrity even in the face of natural thermal and dynamic motions. In summary, the MD simulations offer proof of the complex's stability and the likelihood of a strong and persistent ligand-protein binding.

The structural flexibility of proteins is often assessed using Root Mean Square Fluctuation (RMSF), which measures the movement of individual amino acid residues during an MD simulation. The observed RMSF fluctuations in amino acid residues were compared with RMSD to understand deviations and variations in the complex structure. Residues with the most significant RMSF fluctuations may be responsible for deviations in RMSD. RMSF values were recorded for (a) the combined ligand, 5, (b) the combined target protein, 5MM8, and (c) the combined docked complex between the protein and ligand under four distinct temperature conditions: 300 K, 305 K, 310 K, and 320 K during the 50 ns MD simulation, as shown in Figs. 5d-f and Supplementary Information (SI) Figures S29-S31. In this investigation, the RMSF per residue for the protein-ligand complex was graphed against the atom number (depicted in Fig. 5c-f). The fluctuations observed in the systems ranged from approximately 0.02 to 0.2 nm for the ligand (**5**), 0.03 to 0.45 nm for the target protein (5MM8), and 0.03 to 0.45 nm for the protein-ligand complex, indicating that most of the amino acid residues in the complex displayed fluctuations. The region directly interacting with the ligand (**5**) exhibited low fluctuations, while other regions with amino acids without interactions showed more fluctuations. Notably, some of these amino acids were directly involved in ligand docking binding in all the complexes, as evident from the docking analysis (Fig. 4 and Table 4), and these regions exhibited low fluctuations. The minimal fluctuations in the RMSF plot, as opposed to the increased RMSD at body temperature (310 K), confirmed the stable nature of the amino acid residues of the target protein (PDB: 5MM8).

**Fig. 6:**
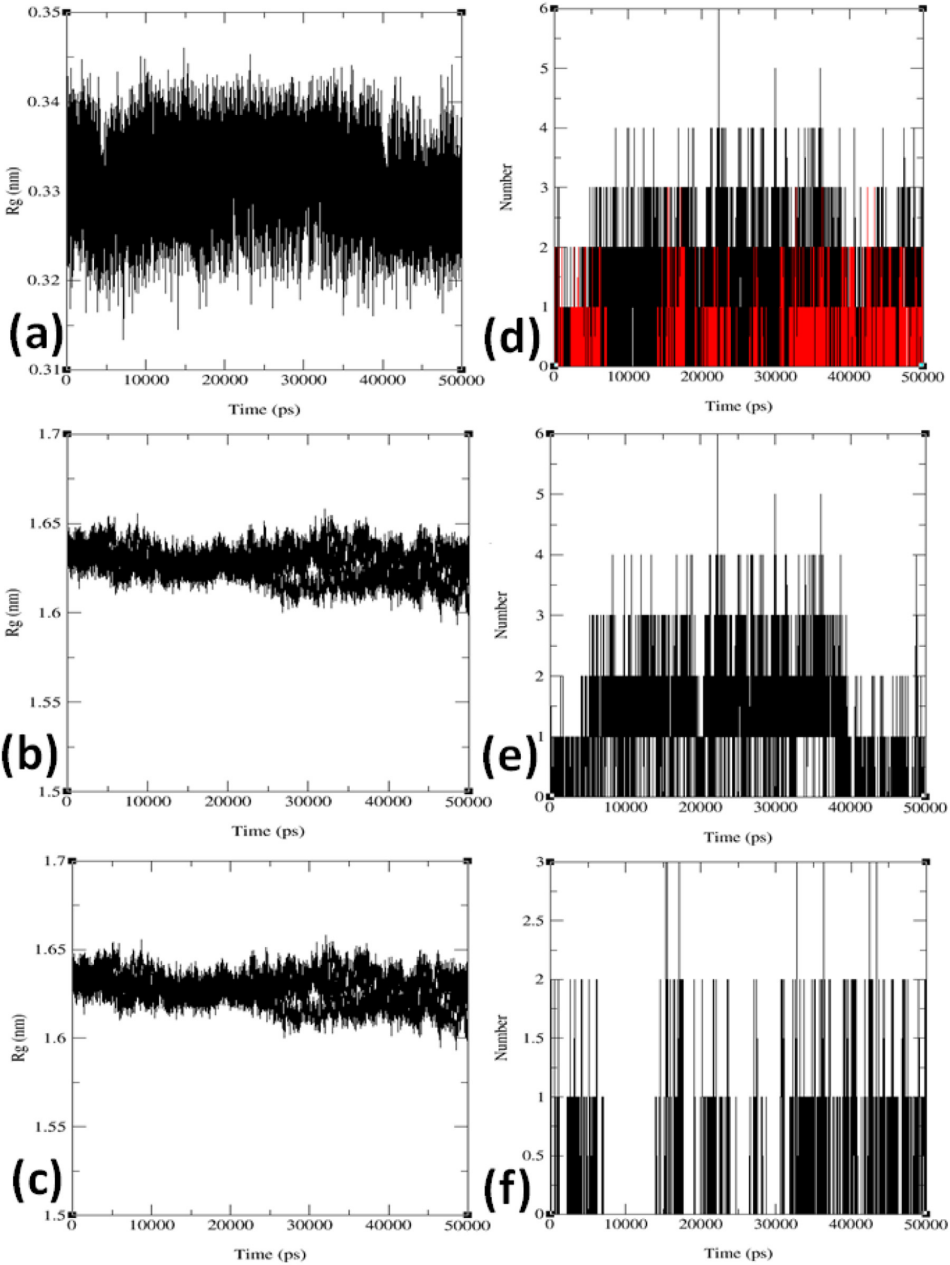
Radius of gyration (Rg in nm) versus time (ps) plots for (a) Rg progression of combined ligand **5** at four different temperature conditions (300 K: black line, 305 K: red line, 310 K: green line, 320 K: blue line); (b) Rg progression of combined target protein (PDB: 5MM8); (c) Rg progression of combined docked complex involving the protein (PDB: 5MM8) and ligand **5**; (d) Intermolecular hydrogen bonding (HBs) stabilization progression for combined protein (ID:5MM8) and ligand **5** under four different temperature conditions; (e) Intermolecular hydrogen bonding stabilization at 310 K; and (f) Intermolecular hydrogen bonding stabilization at 320 K during the 50 ns MD simulation.

The radius of gyration (Rg) served as a metric to quantify conformational changes within the protein structure during the MD simulation. Rg calculations determined the average distance of all dispersed elements from the molecule's center of mass. Furthermore, by correlating the Rg values with RMSD and RMSF, we gained insights into how complex compressibility impacted deviations in complex RMSD and fluctuations observed in RMSF. In this study, the time evolution of Rg for the protein-ligand complex was studied over 50 ns at varied temperatures, as shown in Figs. 6a-c and S32-S34 in the SI. The Rg analysis reveals a slight increase in the Rg values of ligand 5 at four different temperatures after the initial 10 ns, as depicted in Figs. 6a and S32. This increase in Rg may indicate changes in the complex system's overall compactness, which, in turn, can influence residual fluctuations and complex stability. When considering the physiological temperature of 310 K, the analysis of Rg exhibited a sustained compactness of the protein-ligand complex, with minimal fluctuations in Rg values, mirroring the stability observed in the target protein, as illustrated in Figs. 6b-c and S33-S34 in the supplementary information. Based on the analysis of Rg, RMSD, and RMSF for the protein complex at 310 K, it is evident that the compressibility of the complex did not lead to substantial residual fluctuations in RMSF. However, specific residues exhibited elevated RMSF values, which may contribute to the slight increase in RMSD. Overall, examining Rg across all temperature-induced simulations for the protein (IDB: 5MM8) and ligand **5** throughout 50 ns revealed minimal deviation in the plotted Rg values, indicating system stability.

The MD simulation results underscore the importance of the number of hydrogen bonding (HB) interactions between the protein and ligand, which plays a pivotal role in maintaining the stability of the protein-ligand system. These results are depicted in the temporal evaluation of intermolecular hydrogen bonds (HBs) over the 50 ns simulation, as shown in Fig. 6d-f and Supplementary Figure S35. The simulation conducted at body temperature exhibited the most pronounced HB profile, consistently ranging from zero to four hydrogen bonds throughout the entire 50 ns simulation period. Notably, between the 10 ns and 40 ns time frame (Fig. 6e) at 310 K, the ligand exhibited the highest occurrence of hydrogen bond formations with the residues of the target protein (PDB: 5MM8). In the initial evaluation, interactions within the docked complex did not clearly demonstrate the presence of hydrogen bonds (HBs) following molecular docking, as indicated in Fig. 4 and Table 4. However, the subsequent 50 ns MD simulation elucidated the emergence of HB interactions at 300, 305, 310, and 320 K.

**Fig. 7:**
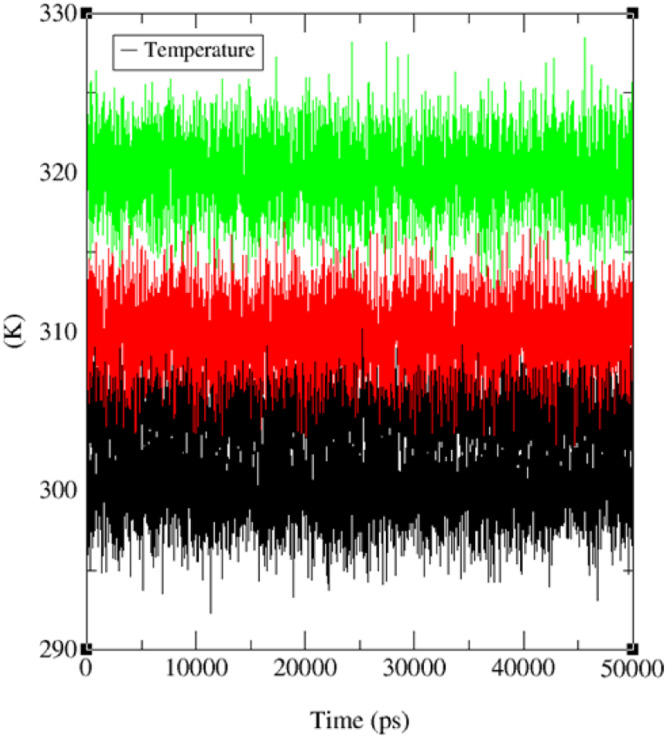
Temperature (K) versus Time (ps) plots for (a) the combined system at four different temperature conditions (300 K: black line, 305 K: blue line, 310 K: red line, 320 K: green line) throughout the 50 ns MD simulation.

**Fig. 8:**
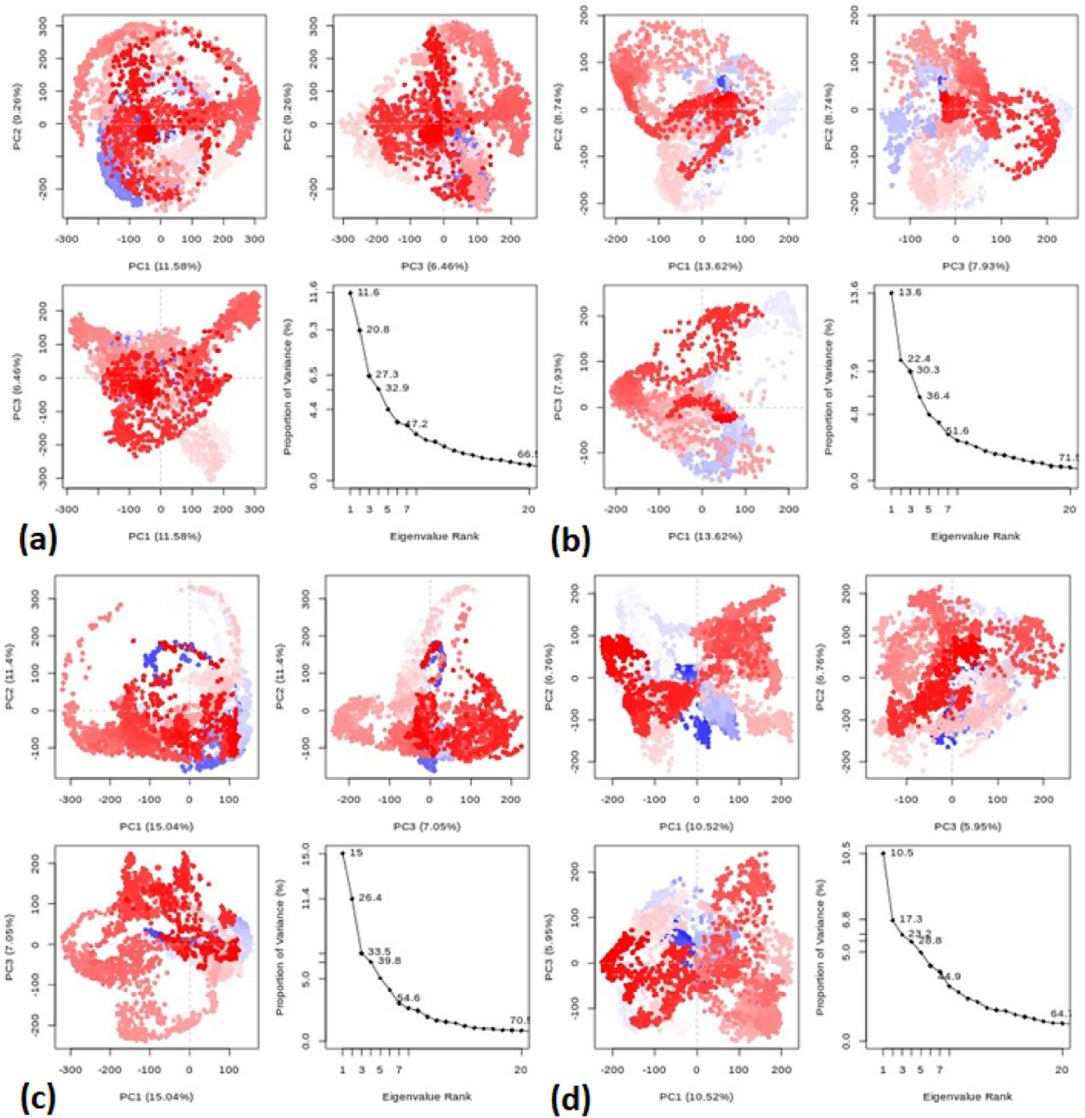
Principal Component Analysis (PCA) of MD Trajectories for the Target Protein (ID: 5MM8) and Ligand 5 Complex at (a) 300 K, (b) 305 K, (c) 310 K, and (d) 320 K (Intermediate states are marked by white dots, energetically unstable conformations are represented by blue dots with scattering, and stable conformation states are denoted by red dots).

The system maintained relatively stable conditions at different temperatures (300, 305, 310, and 320 K), as illustrated in Fig. 7, during the entire 50 ns simulation. Additionally, MD trajectories of the protein (IDB: 5MM8) and ligand **5** at various temperatures underwent conformational principal component analysis (PCA) based on Cα atoms. In this study, Principal Component Analysis (PCA) was employed to assess the variance, collective movements, and alterations in the conformational states of the protein within subsets of the principal components that emerged during the MD simulations as shown in Fig. 8. The PCA investigation, utilizing MD trajectories of the target protein (ID: 5MM8) and ligand **5** complex at temperatures of 300 K, 305 K, 310 K, and 320 K, was conducted using the Bio3D package [71], [72]. The resulting plots of eigenvalues versus eigenvectors are presented in Fig. 8.

The primary motion from the trajectory is isolated within a smaller subset and subsequently compared across the first three eigenvectors (PC1, PC2, and PC3). Colored dots serve to depict the variance captured by these eigenvectors. Specifically, the transition of dot colors, from blue to white to red, signifies the time points during which the complex was sampled. Table 8 presents the primary motions detected within the protein (ID: 5MM8) and ligand **5** complex at different temperatures. These motions were extracted from a reduced dataset and then analyzed across the initial three eigenvectors (PC1, PC2, and PC3). The protein-ligand complex simulated at 310 K showed the highest variability in PC1 (15.04%) with regard to the internal motions of the MD trajectory. While PC2 showed a lower percentage of variance (11.4%) compared to PC1 and the resulting PC3 calculations for protein-ligand complex simulated at different temperatures showed minimum changes depicted from 5.95 to 7.93%. In addition, the principal component analysis values for the data range from 0.30 to 0.68 for 300, 305, 310, and 320 K, respectively, indicating the simulation is converged and the limit range of 0 < *H* < 0.5 [73].

**Table 8:** Variability in principal components revealed via PCA for the target protein and ligand **5** complex at various temperatures.

principal components				
Temperature	PC1 (%)	PC2 (%)	PC3 (%)	cosine value
300	11.58%	9.26%	6.46%	0.68
305	13.62%	8.74%	7.93%	0.30
310	15.04%	11.4%	7.05%	0.35
320	10.52%	6.76%	5.95%	0.31

Based on the simulation outcomes at physiological body temperature (310 K), valuable insights have been gained, suggesting a robust interaction between compound **5** and 5MM8. These results indicate that compound **5** and the protein exhibit favourable interactions, as supported by *in silico* and in vitro investigations. It is, however, imperative to emphasize that additional in vivo studies are essential to confirm the effectiveness and safety of compound **5** as an inhibitor of 5MM8.

## Conclusions

In this study, we synthesized and characterized five new α,β-unsaturated 2-cyanoacetamide derivatives (**1**–**5**) using elemental analysis, FT-IR, and ^1^H NMR spectroscopy. A computational analysis was also conducted on these compounds to evaluate their potential medicinal applications against seven target proteins. Several computational techniques were employed in this study, encompassing DFT calculations, molecular docking assessments, binding energy computations, investigations into HOMO and LUMO properties, analyses of drug-likeness, evaluations of ADMET properties, and molecular dynamics (MD) simulations. *In silico*, compound 5 demonstrated greater binding affinity to *staphylococcus aureus* (PDB: 5MM8) compared to in vitro experiments, indicating its potential as an effective therapeutic agent. Compound 5 exhibited potent effects on 5MM8 cell lines, comparable to the reference drug AG99. Moreover, all of the compounds met multiple criteria for drug-likeness. All the compounds displayed relatively low to moderate acute oral toxicity, indicating their safety for oral administration. Nevertheless, further in vivo experiments are required to confirm the viability of these compounds as pharmaceutical agents.

## CRediT authorship contribution statement

**Kabir M. Uddin:** Formal analysis, Investigation, Methodology, Writing – original draft. **Mehnaz Hossain Meem:** Writing – review & editing. **Mokseda Akter:** Writing – review & editing. **Shofiur Rahman:** Funding acquisition, Writing – review & editing. **Mahmoud A. Al-Gawati:** Funding acquisition, Writing – review & editing. **Nahed Alarifi:** Writing – review & editing. **Hamad Albrithen:** Funding acquisition, Writing – review & editing. **Abdullah Alodhayb:** Funding acquisition, Writing – review & editing. **Raymond A. Poirier:** Conceptualization, Data curation, Funding acquisition, Resources, Supervision, Validation, Writing – original draft, Writing – review & editing. **Md. Mosharef H. Bhuiyan:** Conceptualization, Data curation, Funding acquisition, Resources, Supervision, Validation, Writing – original draft, Writing – review & editing.

## Declaration of competing interest

The author affirms that no identifiable financial conflicts of interest or personal relationships might be perceived as exerting undue influence on the research presented in this paper.

## Data Availability

Data will be made available on request.

## Appendix Supplementary materials

Image, application 1
