# Supplementary material for: Design, synthesis, and bioevaluation of novel unsaturated cyanoacetamide derivatives: In vitro and in silico exploration
Source: MethodsX. 2024 Apr 9;12:102691. doi: 10.1016/j.mex.2024.102691 (PMC11041845; doi:10.1016/j.mex.2024.102691)
Supplement: Supplementary file 1 [file mmc1.docx]

**Design, Synthesis, and Bioevaluation of Novel Unsaturated Cyanoacetamide Derivatives: In Vitro and**

**In Silico Exploration**

**Kabir M. Uddin,^1,^* Mehnaz Hossain Meem,^1^ Mokseda Akter,^2^ Shofiur Rahman,^3^ Mahmoud A. Al-Gawati,^3^ Nahed Alarifi,^4^ Hamad Albrithen,^3,4^ Abdullah Alodhayb,^3,4^* Raymond A. Poirier^5^ and Md. Mosharef H. Bhuiyan^2,^***

^1^Department of Biochemistry and Microbiology, North South University, Bashundhara,

Dhaka-1217, Bangladesh

^2^Bioorganic and Medicinal Chemistry Laboratory, Department of Chemistry,

University of Chittagong, Chattogram, 4331, Bangladesh

^3^Biological and Environmental Sensing Research Unit, King Abdullah Institute for Nanotechnology, King Saud University, Riyadh 11451, Saudi Arabia

^4^Research Chair for Tribology, Surface, and Interface Sciences, Department of Physics and Astronomy, College of Science, King Saud University, Riyadh 11451, Saudi Arabia

^5^Department of Chemistry, Memorial University, St. John’s, Newfoundland,

Canada A1B 3X7

Tel.: +8801796585904

Fax: +8802-55668202

E-mail: [mohammed.uddin11@northsouth.edu](mailto:mohammed.uddin11@northsouth.edu)

E-mail: [kabirmuddin@gmail.com](mailto:kabirmuddin@gmail.com)

E-mail: [mosharefchem@cu.ac.bd](mailto:mosharefchem@cu.ac.bd)

E-mail: [aalodhayb@ksu.edu.sa](mailto:aalodhayb@ksu.edu.sa)

**Synthesis of 2-(4-hydroxybenzylidene)-cyanoacetamide (1):**

The titled compound **1** was synthesized from 4-hydroxybenzaldehyde, 2-cyanoacetamide and ammonium acetate under microwave condition.

4-Hydroxybenzaldehyde (0.4885g, 4mmol) and 2-cyanoacetamide (0.33632g, 4 mmol) were placed in a porcelain dish. Then 10 mg of ammonium acetate was added and mixed with them. Then the porcelain dish was taken in the microwave oven. The mixture was irradiated under 160 watt microwave irradiation for 40 seconds only. The progress of the reaction was monitored by TLC (*n*-hexane : ethyl acetate, 6:1). The product was obtained as a reddish black brown solid. The crude solid was recrystallized from ethyl acetate and *n*-hexane to give **1** as black brown crystalline solid.

Yield: 0.2596 gm (98.65%), Melting Point: 114-116ºC

The structural analyses of the compound **1** by spectroscopic methods are given in below:

IR (KBr) γ_max_ (cm^-1^): 3400.15, 3313.71 (N-H), 3367.71 (b, OH), 3197.98(C-H), 2229.71 (s, C≡N), 1685.79 (C=O, amide), 1600.00 (s, C=C), 1573.91 (s, C=C, Ph).

^1^H-NMR (400 MHz, CD_3_OD): δ_H_ (ppm): 8.09 (s, 1H, CH), 7.90 (d, 2H, *J*=8.8 Hz, H-2, H-6, Ph), 6.90 (d, 2H, *J* = 8.8 Hz, H-3, H-5, Ph), 4.84 (s, 2H, -NH_2_), 3.30 (s, OH).

**Synthesis of 2-(4-ethoxybenzylidene)-cyanoacetamide (2):**

2-(4-Ethoxybenzylidene)-cyanoacetamide (**2**) was synthesized from 4-ethoxybenzaldehyde, 2-cyanoacetamide and ammonium acetate under microwave condition.

4-Ethoxybenzaldehyde (0.54g, 3.5975 mmol) and 2-cyanoacetamide (0.3023 g, 3.5957 mmol) were placed in a porcelain dish. Then 10 mg of ammonium acetate was added and mixed with them. Then the porcelain dish was taken in the microwave oven. The mixture was irradiated under 160 watt microwave irradiation for 30 seconds. The progress of the reaction was monitored by TLC (*n*-hexane : ethyl acetate, 6:1). The product was obtained as a yellow solid crystal. The white crude solid was recrystallized from ethyl acetate and *n*-hexane to give **2** as a white crystalline solid.

Yield: 0.3077 g (94%), Melting Point: 156-158°C

The structural analyses of the compound **2** by spectroscopic methods are given in below:

IR (KBr) γ_max_ (cm^-1^): 3441.01, 3394.72 (N-H), 3159.40 (C-H), 2210.42 (s, C≡N), 1705.07 (s, C=O, amide), 1590.00 (s, C=C), 1508.33 (s, C=C, Ph).

^1^H-NMR (400 MHz, CD_3_OD): δ_H_ (ppm): 8.13 (s, 1H, C-H), 7.94 (d, 2H, H-2, H-6, *J* = 8.8 Hz, Ph), 7.01 (d, 2H, H-3, H-5, *J* =8.8 Hz, Ph), 4.77 (s, 2H, -NH_2_), 4.12 (q, 2H, OCH_2_), 1.42 ( t, 3H, *J*=7.2 Hz, CH_3_).

**Synthesis of 2-(3-methoxybenzylidene)-cyanoacetamide (3):**

2-(3-Methoxybenzylidene)-cyanoacetamide **(3)** was synthesized from 3-methoxybenzaldehyde, 2-cyanoacetamide and ammonium acetate under microwave condition.

At first, 3-methoxybenzaldehyde (0.4182g, 3.0704 mmol) and 2-cyanoacetamide (0.2582g, 3.0704 mmol) were placed in a porcelain dish. Then 10 mg of ammonium acetate was added and mixed with them. Then the porcelain dish was taken in the microwave oven. The mixture was irradiated under 160 watt microwave irradiation for 30 seconds. The progress of the reaction was monitored by TLC (*n*-hexane : ethyl acetate, 4:1). The product was obtained as an off-white solid. The crude solid was recrystallized from ethyl acetate and *n*-hexane to give **3** as an off-white crystalline solid.

Yield: 0.3538 gm (76.03%), Melting Point: 112-114ºC

The structural analyses of the compound **3** by spectroscopic methods are given in below:

IR (KBr) γ_max_ (cm^-1^): 3394.72, 3313.71 (N-H), 3190.26 (C-H), 2222.00 (s, C≡N), 1716.65 (s, C=O, amide), 1631.78, 1593.20 (s, C=C).

^1^H-NMR (400 MHz, CD_3_OD): δ_H_ (ppm): 8.16 (s, 1H, CH), 7.57 (s, 1H, H-2), 7.48 (d, 1H, H-6, *J*=8.0 Hz, Ph), 7.42 (t, 1H, H-5, *J*=8.0), 7.12 (d, 1H, H-4, *J* = 8.0 Hz, Ph), 4.84 (s, 2H, -NH_2_), 3.84 (s, 3H, OCH_3_).

**Synthesis of 2-(4-nitrobenzylidene)-cyanoacetamide (4**):

2-(4-nitrobenzylidene)-cyanoacetamide **(4)** was synthesized from 4-nitrobenzaldehyde, 2-cyanoacetamide and ammonium acetate under microwave condition.

4-Nitrobenzaldehyde (0.45336g, 3 mmol) and 2-cyanoacetamide (0.25224g, 3 mmol) were placed in a porcelain dish. Then 10 mg of ammonium acetate was added and mixed with them. Then the porcelain dish was taken in the microwave oven. The mixture was irradiated under 320 watt microwave irradiation for 70 seconds. The progress of the reaction was monitored by TLC (*n*-hexane : ethyl acetate, 6:1). The product was obtained as a brown solid. The crude solid was recrystallized from ethyl acetate and *n*-hexane to give **4** as a deep brown crystalline solid.

Yield: 0.3752 gm (99.15%), Melting Point: 208-210°C

The structural analyses of the compound **4** by spectroscopic methods are given in below:

IR (KBr) γ_max_ (cm^-1^): 3441.01, 3344.51 (N-H), 3197.98 (C-H), 2225.00 (s, C≡N), 1697.36 (C=O, amide), 1600.90 (s, C=C, Ph).

^1^H-NMR (400 MHz, CD_3_OD): δ_H_ (ppm): 8.36 (d, 1H, *J*=8.8 Hz, H-2, Ph), 8.29 (s, 1H, CH), 8.21 (d, 1H, H-3, *J* = 8.8 Hz, Ph), 8.15 (d, 1H, H-5, *J* = 8.84 Hz, Ph), 7.65 (d, 1H, H-6, *J* = 8.8 Hz, Ph), 4.85 (s, 2H, -NH_2_).

**Synthesis of 2-(3,4-methylenedioxybenzylidene)-cyanoacetamide (5):**

2-(3,4-methylenedioxybenzylidene)-cyanoacetamide  **(5)** was synthesized from piperonal, 2-cyanoacetamide and ammonium acetate under microwave condition.

Piperonal (0.45039g, 3 mmol) and 2-cyanoacetamide (0.25224g, 3 mmol) were placed in a porcelain dish. Then 10 mg of ammonium acetate was added and mixed with them. Then the porcelain dish was taken in the microwave oven. The mixture was irradiated under 160 watt microwave irradiation for 60 sec. The progress of the reaction was monitored by TLC (*n*-hexane : ethyl acetate, 6:1). The product was obtained as a greenish yellow solid. The crude solid was recrystallized from ethyl acetate and *n*-hexane to give **5** as a greenish yellow crystalline solid.

Yield: 0.3738gm (97.3%), Melting Point: 178-180°C

The structural analyses of the compound **5** by spectroscopic methods are given in below:

IR (KBr) γ_max_ (cm^-1^): 3475.73, 3375.77 (N-H), 3132.40 (C-H), 2214.28 (s, C≡N), 1716.65 (C=O, amide), 1577.77 (s, C=C).

^1^H-NMR (400 MHz, CD_3_OD): δ_H_ (ppm): 8.08 (s, 1H, CH), 7.65 (s, 1H, H-2, Ph), 7.43 (d, 1H, H-6, *J* = 8.0 Hz, Ph), 6.97 (d, 1H, H-5, *J* = 8.0 Hz, Ph), 6.09 (s, 2H, OCH_2_O), 4.84 (s, 2H, -NH_2_).

**Table S1**

Optimized structure for 2-(4-hydroxybenzylidene)-cyanoacetamide (1) and Cartesian Z-matrix.

| **2-(4-hydroxybenzylidene)-cyanoacetamide (1)** | | | 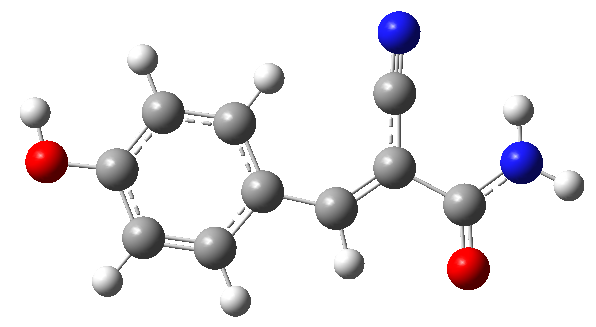 | | |
| --- | --- | --- | --- | --- | --- |
| Center  Number | Atomic  Symbol | Standard orientation: Coordinates (Angstroms) | | | |
|  |  | X | | Y | Z |
| 1 | O | 3.274167 | | -1.872634 | 0.000097 |
| 2 | O | -4.803786 | | 0.173729 | -0.000132 |
| 3 | N | 4.294754 | | 0.157275 | -0.000186 |
| 4 | N | 1.945409 | | 2.648882 | 0.000052 |
| 5 | C | 3.199302 | | -0.649886 | 0.000001 |
| 6 | C | 1.8622 | | 0.060497 | 0.000043 |
| 7 | C | -0.666138 | | -0.397177 | 0.000075 |
| 8 | C | 0.746155 | | -0.718672 | 0.000044 |
| 9 | C | -1.577411 | | -1.47827 | 0.000039 |
| 10 | C | -1.201787 | | 0.909282 | 0.000081 |
| 11 | C | -2.948005 | | -1.280606 | -0.000015 |
| 12 | C | -2.572751 | | 1.117496 | 0.000052 |
| 13 | C | -3.454274 | | 0.02586 | -0.000032 |
| 14 | C | 1.86356 | | 1.484811 | 0.000063 |
| 15 | H | 4.231635 | | 1.163176 | -0.000387 |
| 16 | H | 0.986884 | | -1.780789 | 0.000039 |
| 17 | H | -1.187875 | | -2.492331 | -0.000011 |
| 18 | H | -0.545531 | | 1.770713 | 0.000192 |
| 19 | H | -2.966219 | | 2.131459 | 0.000096 |
| 20 | H | -5.027051 | | 1.114494 | -0.000234 |
| 21 | H | 5.200411 | | -0.28444 | -0.000485 |
| 22 | H | -3.641553 | | -2.114156 | -0.000083 |

**Table S2**

Optimized structure for 2-(4-ethoxybenzylidene)-cyanoacetamide (2) and Cartesian Z-matrix.

| 2-(4-ethoxybenzylidene)-cyanoacetamide (**2**) | | | 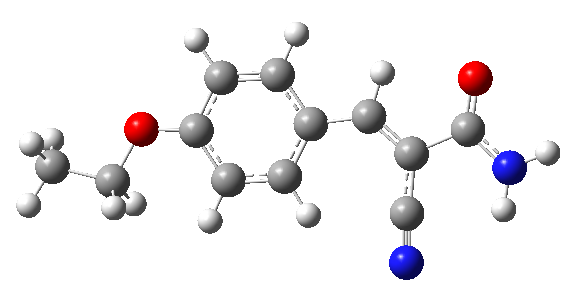 | | |
| --- | --- | --- | --- | --- | --- |
| Center  Number | Atomic  Symbol | Standard orientation: Coordinates (Angstroms) | | | |
|  |  | X | | Y | Z |
| 1 | O | -4.343457 | | 1.65234 | 0.000151 |
| 2 | O | 3.899045 | | 0.442563 | 0.000476 |
| 3 | N | -5.161041 | | -0.46728 | -0.000843 |
| 4 | N | -2.584436 | | -2.718568 | 0.000297 |
| 5 | C | -4.148955 | | 0.442652 | -0.000074 |
| 6 | C | -2.749612 | | -0.134173 | -0.000003 |
| 7 | C | -0.277597 | | 0.56783 | 0.000018 |
| 8 | C | -1.713951 | | 0.749769 | 0.000005 |
| 9 | C | 0.525556 | | 1.73326 | -0.000051 |
| 10 | C | 0.384256 | | -0.677111 | 0.000084 |
| 11 | C | 1.905844 | | 1.664723 | 0.000086 |
| 12 | C | 1.771183 | | -0.759705 | 0.000225 |
| 13 | C | 2.545163 | | 0.412958 | 0.000259 |
| 14 | C | -2.613473 | | -1.551927 | 0.000126 |
| 15 | H | -5.00023 | | -1.462221 | 0.000686 |
| 16 | H | -2.057051 | | 1.783414 | -0.000077 |
| 17 | H | 0.040996 | | 2.705608 | -0.000056 |
| 18 | H | -0.1857 | | -1.598126 | 0.00005 |
| 19 | H | 2.241098 | | -1.735307 | 0.000321 |
| 20 | H | -6.105222 | | -0.115534 | 0.000163 |
| 21 | H | 2.518065 | | 2.559926 | 0.000089 |
| 22 | C | 4.627701 | | -0.791818 | -0.000032 |
| 23 | H | 4.358751 | | -1.378189 | -0.888635 |
| 24 | H | 4.359613 | | -1.378445 | 0.888667 |
| 25 | C | 6.105431 | | -0.447612 | -0.000646 |
| 26 | H | 6.702134 | | -1.364573 | -0.001037 |
| 27 | H | 6.365556 | | 0.136172 | -0.88764 |
| 28 | H | 6.366359 | | 0.135919 | 0.886278 |

**Table S3**

Optimized structure for 2-(3-methoxybenzylidene)-cyanoacetamide (3) and cartesian Z-matrix.

| 2-(3-methoxybenzylidene)-cyanoacetamide (**3**) | | | 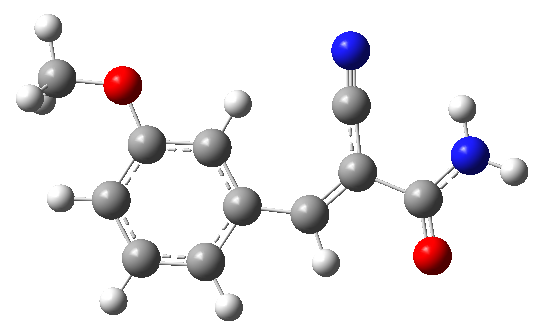 | | |
| --- | --- | --- | --- | --- | --- |
| Center  Number | Atomic  Symbol | Standard orientation: Coordinates (Angstroms) | | | |
|  |  | X | | Y | Z |
| 1 | O | -3.881345 | | 1.445989 | -0.000044 |
| 2 | N | -4.439401 | | -0.756712 | -0.000167 |
| 3 | N | -1.636842 | | -2.687934 | 0.000209 |
| 4 | C | -3.546111 | | 0.268406 | -0.000062 |
| 5 | C | -2.083381 | | -0.136904 | 0.000101 |
| 6 | C | 0.290249 | | 0.855527 | -0.000003 |
| 7 | C | -1.165972 | | 0.863843 | 0.000024 |
| 8 | C | 0.931912 | | 2.114626 | 0.000246 |
| 9 | C | 1.077926 | | -0.30632 | -0.000121 |
| 10 | C | 2.31715 | | 2.194264 | 0.000148 |
| 11 | C | 2.473818 | | -0.217714 | -0.000249 |
| 12 | C | 3.100264 | | 1.036781 | -0.000217 |
| 13 | C | -1.783877 | | -1.530874 | 0.000208 |
| 14 | H | -4.158997 | | -1.724904 | -0.000291 |
| 15 | H | -1.628927 | | 1.849208 | -0.000144 |
| 16 | H | 0.331334 | | 3.01873 | 0.000356 |
| 17 | H | 0.638602 | | -1.295656 | 0.000048 |
| 18 | H | -5.41951 | | -0.522449 | -0.000272 |
| 19 | H | 2.804227 | | 3.164553 | 0.000164 |
| 20 | H | 4.180223 | | 1.120592 | -0.000391 |
| 21 | O | 3.133912 | | -1.40925 | -0.000065 |
| 22 | C | 4.553913 | | -1.397954 | 0.000062 |
| 23 | H | 4.954937 | | -0.904543 | 0.894454 |
| 24 | H | 4.860856 | | -2.444345 | 0.000156 |
| 25 | H | 4.955083 | | -0.904652 | -0.894325 |

**Table S4**

Optimized structure for 2-(4-nitrobenzylidene)-cyanoacetamide (4) and cartesian Z-matrix

| 2-(4-nitrobenzylidene)-cyanoacetamide (**4**) | | |  | | |
| --- | --- | --- | --- | --- | --- |
| Center  Number | Atomic  Symbol | Standard orientation: Coordinates (Angstroms) | | | |
|  |  | X | | Y | Z |
| 1 | O | 3.99662 | | -1.838044 | -0.000386 |
| 2 | N | 4.983122 | | 0.210727 | 0.000107 |
| 3 | N | 2.591863 | | 2.657988 | -0.000038 |
| 4 | C | 3.907861 | | -0.618129 | -0.00014 |
| 5 | C | 2.549466 | | 0.068296 | -0.000071 |
| 6 | C | 0.030156 | | -0.436146 | 0.000222 |
| 7 | C | 1.457263 | | -0.735268 | 0.000072 |
| 8 | C | -0.849076 | | -1.540311 | 0.000143 |
| 9 | C | -0.521347 | | 0.863292 | 0.000335 |
| 10 | C | -2.226872 | | -1.367996 | 0.000129 |
| 11 | C | -1.897544 | | 1.048074 | 0.000182 |
| 12 | C | -2.730671 | | -0.069919 | 0.000031 |
| 13 | C | 2.523861 | | 1.493953 | -0.000025 |
| 14 | H | 4.900488 | | 1.215445 | 0.000374 |
| 15 | H | 1.718388 | | -1.792196 | -0.000002 |
| 16 | H | -0.43718 | | -2.544716 | -0.000001 |
| 17 | H | 0.12107 | | 1.734391 | 0.000513 |
| 18 | H | -2.335591 | | 2.037979 | 0.000311 |
| 19 | H | 5.898838 | | -0.210922 | 0.000108 |
| 20 | H | -2.909667 | | -2.207853 | 0.000069 |
| 21 | N | -4.191319 | | 0.127856 | -0.000117 |
| 22 | O | -4.900664 | | -0.877129 | 0.000149 |
| 23 | O | -4.608529 | | 1.284772 | -0.000549 |

**Table S5**

Optimized structure for 2-(3,4-methylenedioxybenzylidene)-cyanoacetamide (5) and cartesian Z-matrix

| 2-(3,4-methylenedioxybenzylidene)-cyanoacetamide (**5**) | | | 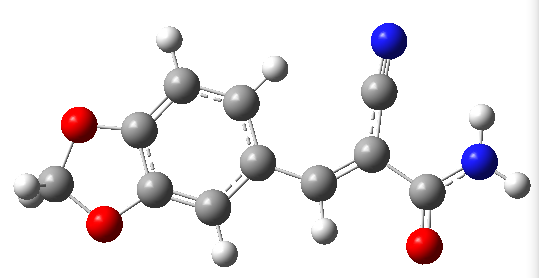 | | |
| --- | --- | --- | --- | --- | --- |
| Center  Number | Atomic  Symbol | Standard orientation: Coordinates (Angstroms) | | | |
|  |  | X | | Y | Z |
| 1 | O | -3.61256 | | -2.088844 | -0.000105 |
| 2 | N | -4.881998 | | -0.204579 | 0.000305 |
| 3 | N | -2.876565 | | 2.563146 | -0.000062 |
| 4 | C | -3.693481 | | -0.866529 | 0.000036 |
| 5 | C | -2.456408 | | 0.008039 | 0.000032 |
| 6 | C | 0.112714 | | -0.135502 | 0.00001 |
| 7 | C | -1.252315 | | -0.626029 | 0.000044 |
| 8 | C | 1.13716 | | -1.125437 | -0.000039 |
| 9 | C | 0.469111 | | 1.229671 | 0.000047 |
| 10 | C | 2.442616 | | -0.698406 | -0.000076 |
| 11 | C | 1.804958 | | 1.645101 | -0.000037 |
| 12 | C | 2.77416 | | 0.656835 | -0.000103 |
| 13 | C | -2.641166 | | 1.420273 | -0.000013 |
| 14 | H | -4.946728 | | 0.801238 | -0.000532 |
| 15 | H | -1.36227 | | -1.709408 | 0.000095 |
| 16 | H | 0.891451 | | -2.181366 | -0.000087 |
| 17 | H | -0.302389 | | 1.988537 | 0.000141 |
| 18 | H | 2.072134 | | 2.695218 | -0.00002 |
| 19 | H | -5.724395 | | -0.757495 | -0.000432 |
| 20 | O | 4.130143 | | 0.803748 | -0.000192 |
| 21 | O | 3.589168 | | -1.453604 | -0.000297 |
| 22 | C | 4.679255 | | -0.52552 | 0.000435 |
| 23 | H | 5.283555 | | -0.665825 | 0.903919 |
| 24 | H | 5.284955 | | -0.666238 | -0.902032 |

**Table S6**

Protein and ligand interacting amino acid residues of compound **5** against

three targets

| Target (PDB ID) | | interacting residues | distance | | | type of interaction | | 2D diagram of interaction | | |
| --- | --- | --- | --- | --- | --- | --- | --- | --- | --- | --- |
| 6SE1 | | HIS A:422  LYS A:421  SER A:527  GLN A:490  GLU a:492 | | 3.95  2.65  2.26  3.02 | | π-Alkyl  Conventional HB  Conventional HB  Conventional HB  Conventional HB | | | 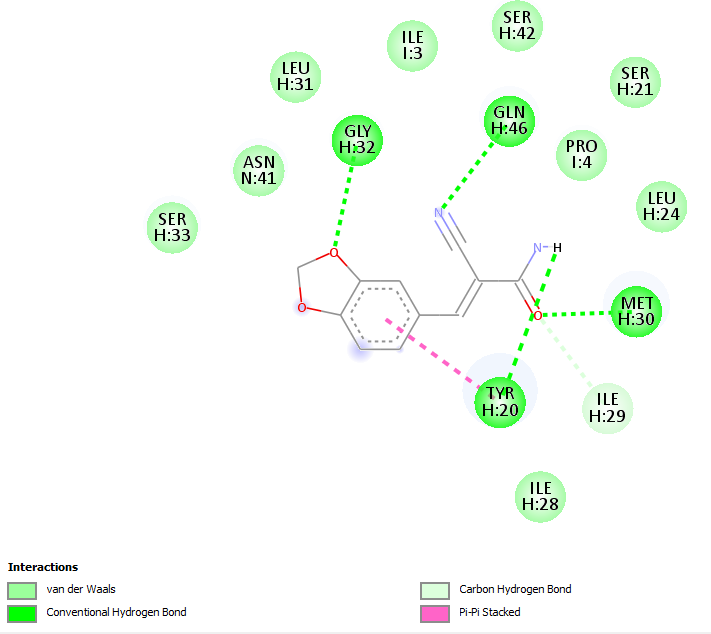 | |
| 5GVZ | | GLN A:1  GLN A:57  ARG A:60  GLU A:58  LYS A:4 | 3.22  2.52  2.23  3.01  4.67 | | | π Conventional HB  Conventional HB Conventional HB  Carbon HB  π-Alkyl | | | | 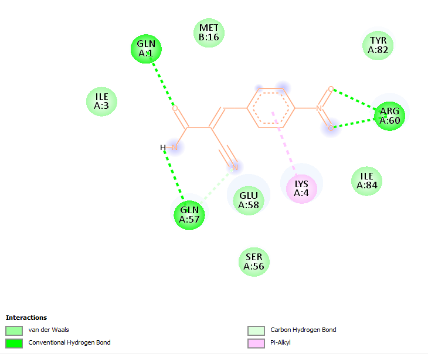 |
| 1TM2 | | TYR A:194  PRO A:68  THR A:163  VAL A:93  ASN A:195  SER A:161  SER A:160 | 4.03  4.48  2.65  2.76  2.97  2.56  2.87 | | Pi-Pi T shaped  Pi-Alkyl  Conventional HB Conventional HB Conventional Conventional HB  Carbon HB | | 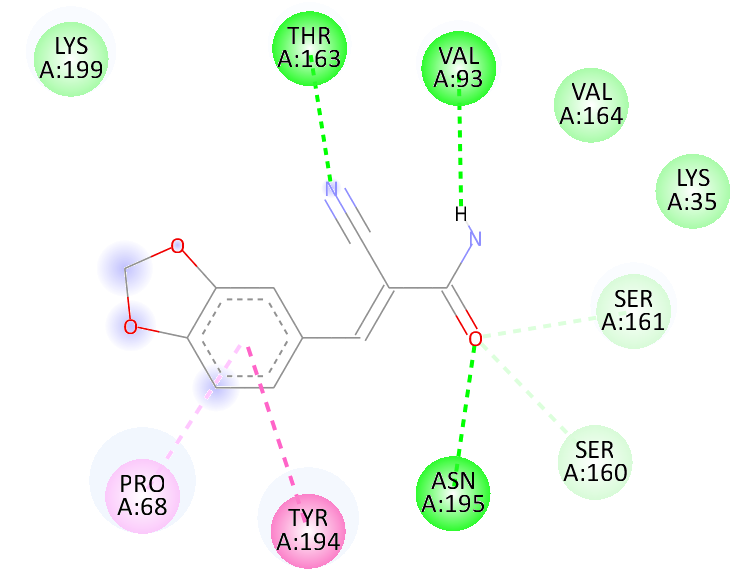 | | | |
|  | | | | | | | | | |  |

**Table S7**

Physicochemical parameters of drug-likeness properties for the synthesized cyanoacetamide derivatives (**1−5**) using SwissADME*^a^*

| **Drugs** | **1** | **2** | **3** | **4** | **5** |
| --- | --- | --- | --- | --- | --- |
| **Physico-chemical properties** |  |  |  |  |  |
| Fraction Csp3 | 0.00 | 0.17 | 0.09 | 0.00 | 0.09 |
| Rotatable bonds | 2 | 4 | 3 | 3 | 2 |
| H-bond acceptors | 3 | 3 | 3 | 4 | 4 |
| H-bond donors | 2 | 1 | 1 | 1 | 1 |
| Molar refractivity | 50.83 | 60.10 | 55.30 | 57.63 | 54.87 |
| Molecular weight | 188.18 | 216.24 | 202.21 | 217.18 | 216.19 |
| TPSA | 87.11 | 76.11 | 76.11 | 112.7 | 85.34 |
| MLOGP | 0.25 | 0.82 | 0.54 | -0.27 | -0.01 |
| LogS (ESOL) | -1.95 | -2.39 | -1.03 | -2.14 | -0.25 |
| **Drug-likeliness filters** |  |  |  |  |  |
| Lipinski violations | Yes, 0 | Yes, 0 | Yes, 0 | Yes, 0 | Yes, 0 |
| Ghose violations | Yes | Yes | Yes | Yes | Yes |
| Veber violations | Yes | Yes | Yes | Yes | Yes |
| Egan violations | Yes | Yes | Yes | Yes | Yes |
| Muegge violations | No,1 (MW<200) | Yes | Yes | Yes | Yes |
| Bioavailability score | 0.55 | 0.55 | 0.55 | 0.55 | 0.55 |
| Pains1 | 0 alert | 0 alert | 0 alert | 0 alert | 0 alert |
|  |  |  |  |  |  |

*^a^* Physicochemical Parameters of drug likeness properties data for synthesized cyanoacetamide

derivatives **(1−5)** defined in ESM_Publication material in the SI.

| 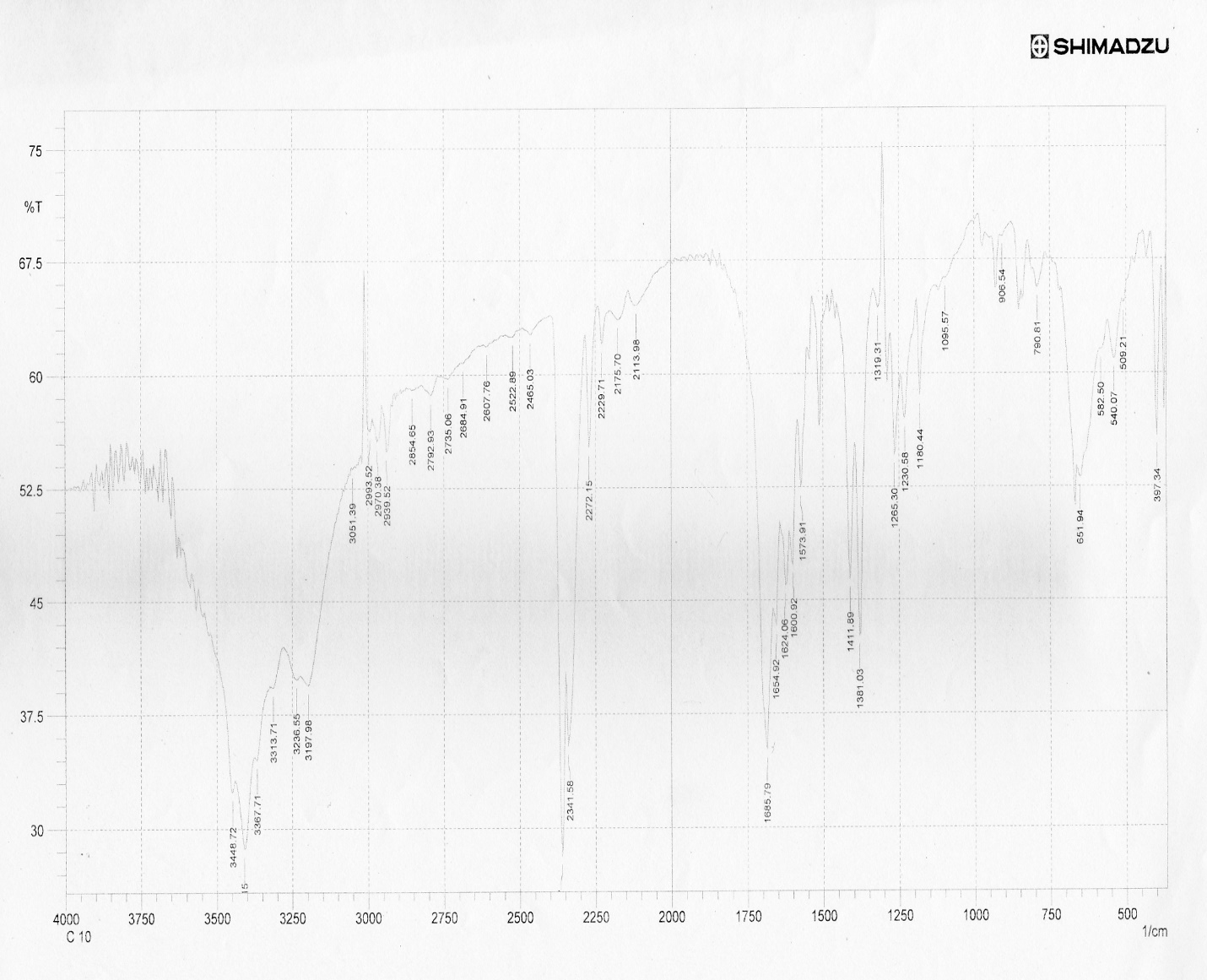 |
| --- |
|  |
| **Fig. S1.** FTIR spectrum of compound **1**. |

| **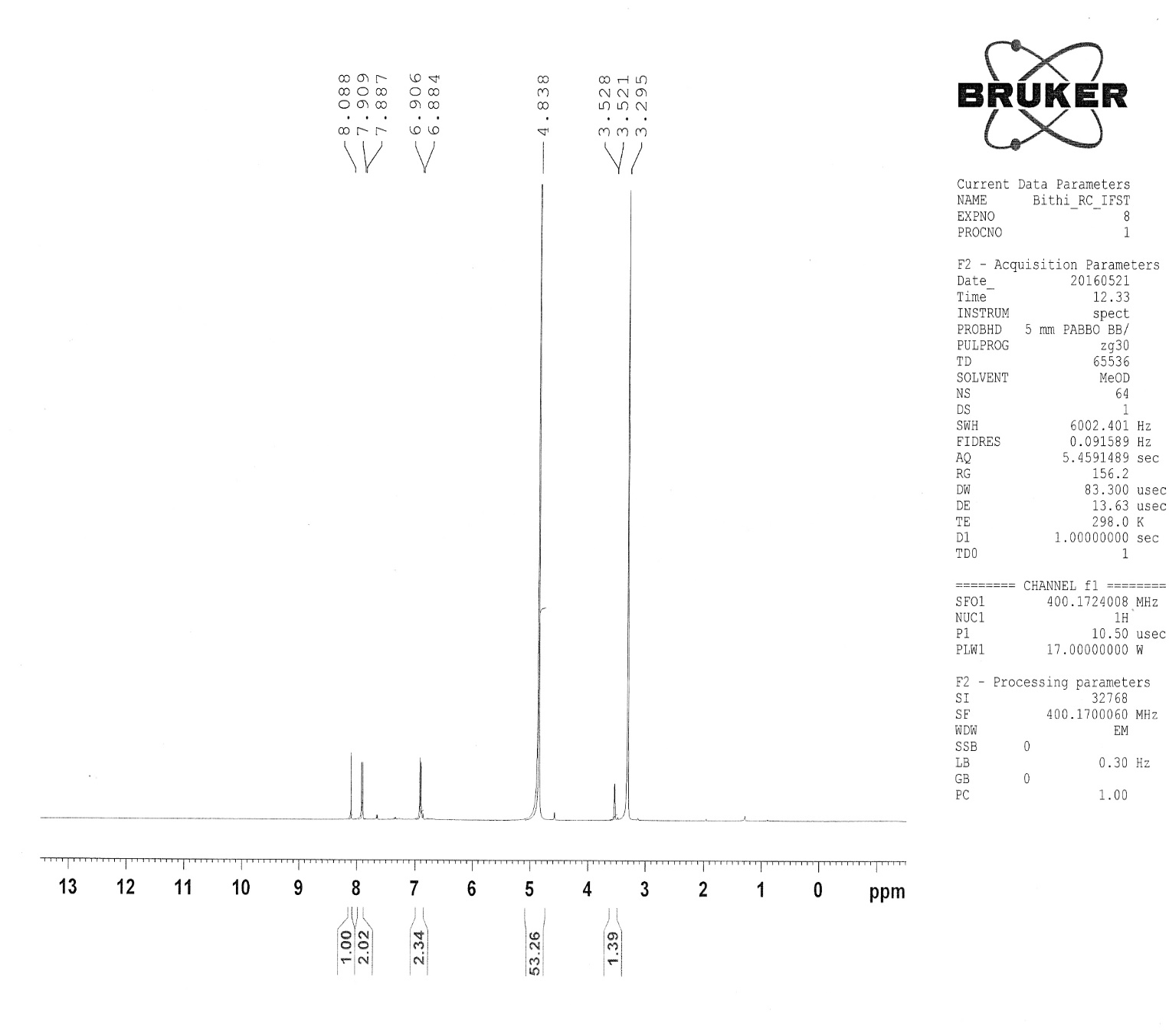** |
| --- |
|  |
| **Fig. S2. ^1^**H NMR spectrum of compound **1**. |

| 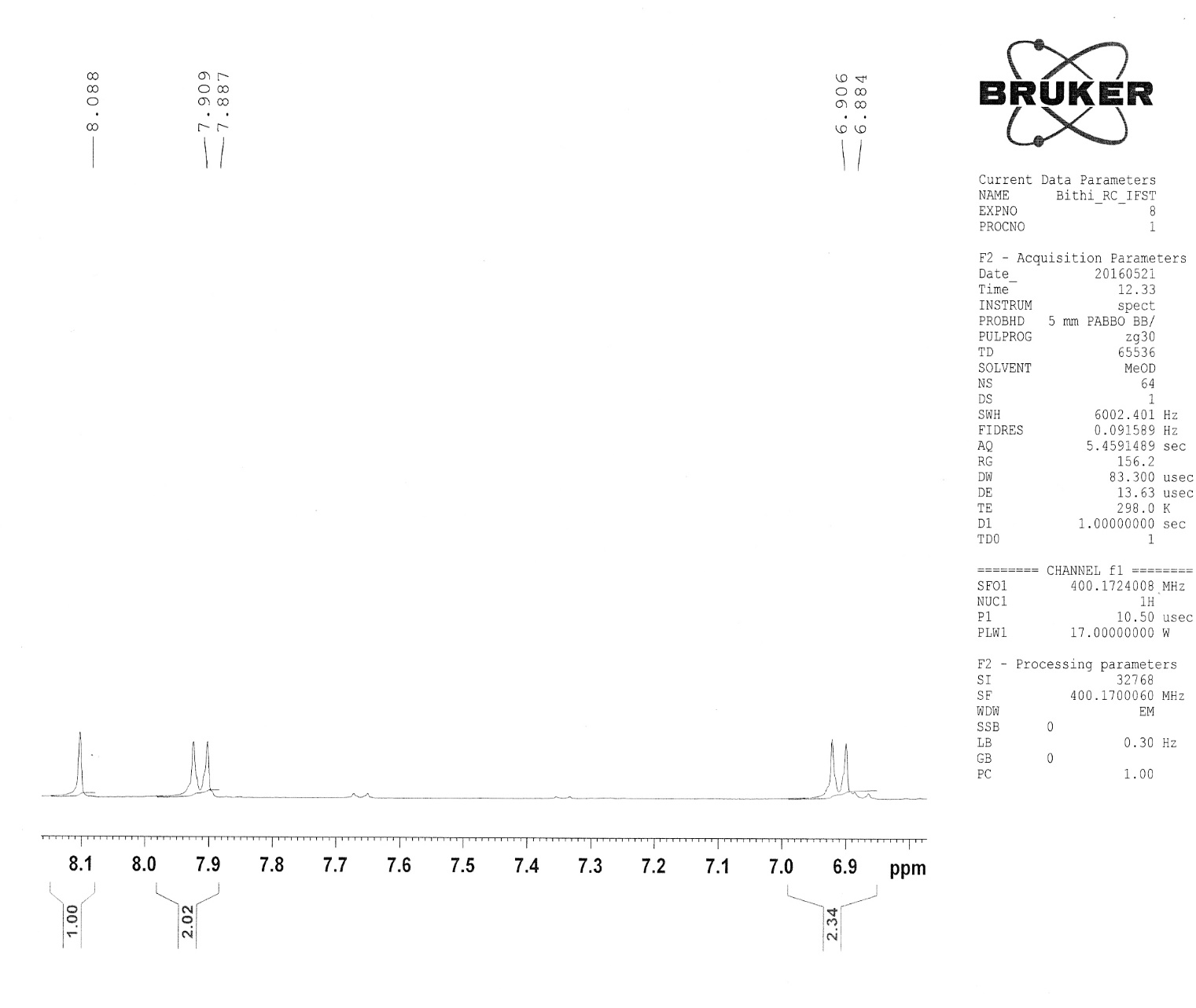 |
| --- |
|  |
| **Fig. S3. ^1^**H NMR spectrum of compound **1**. |
| 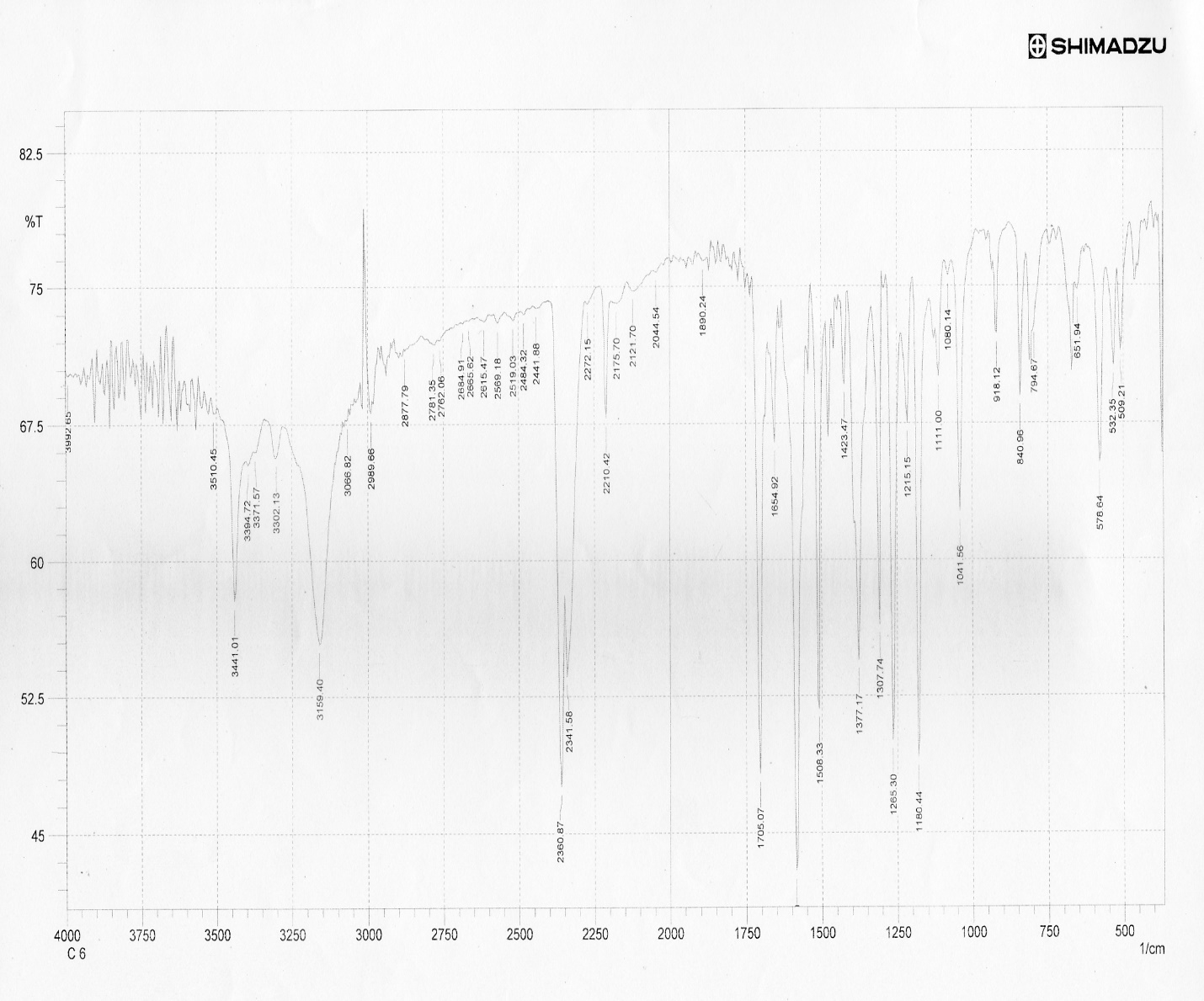 |
|  |
| **Fig. S4. FTIR** spectrum of compound **2.** |

| **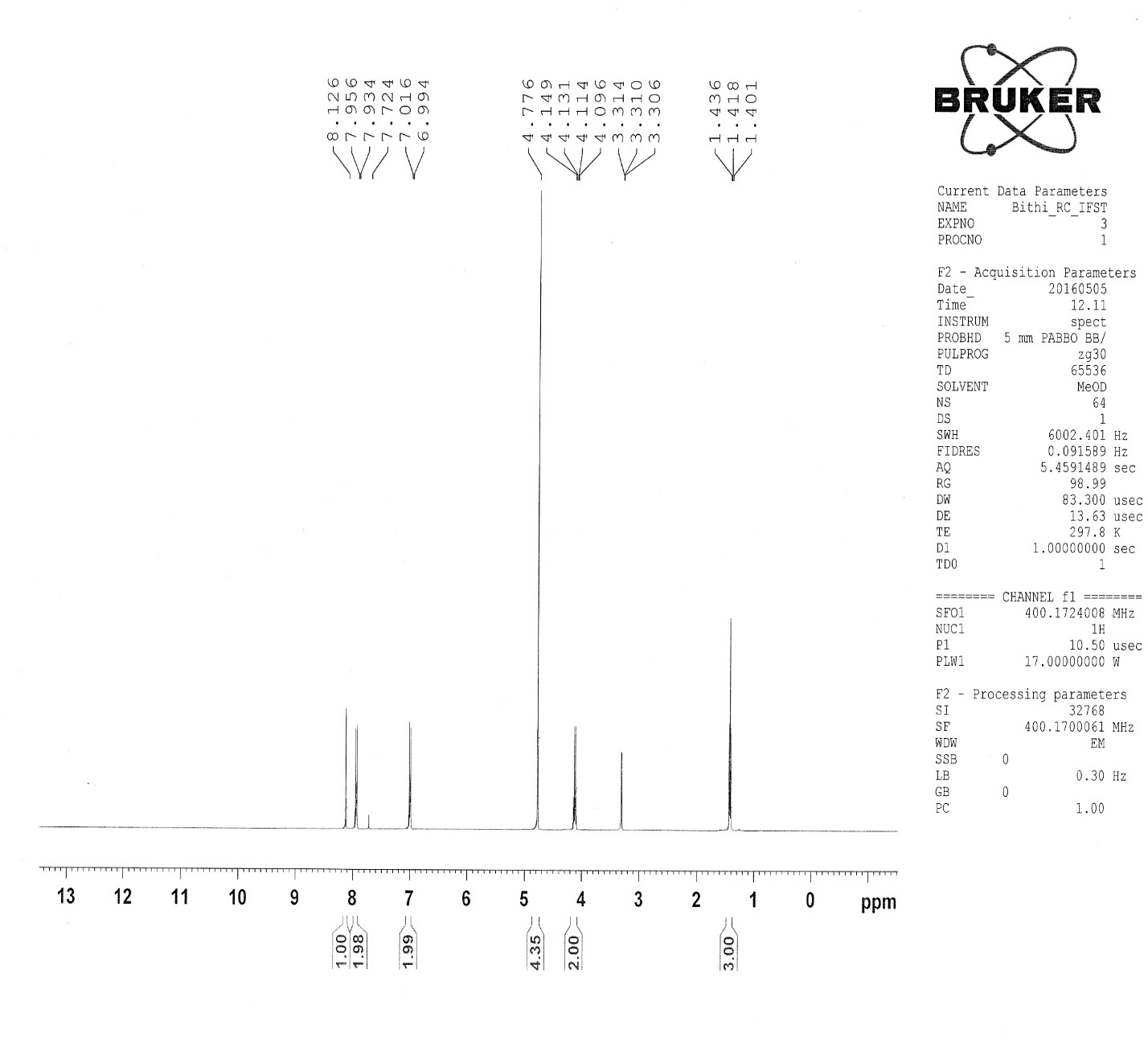** |
| --- |
|  |
| **Fig. S5. ^1^**H NMR spectrum of compound **2**. |

| 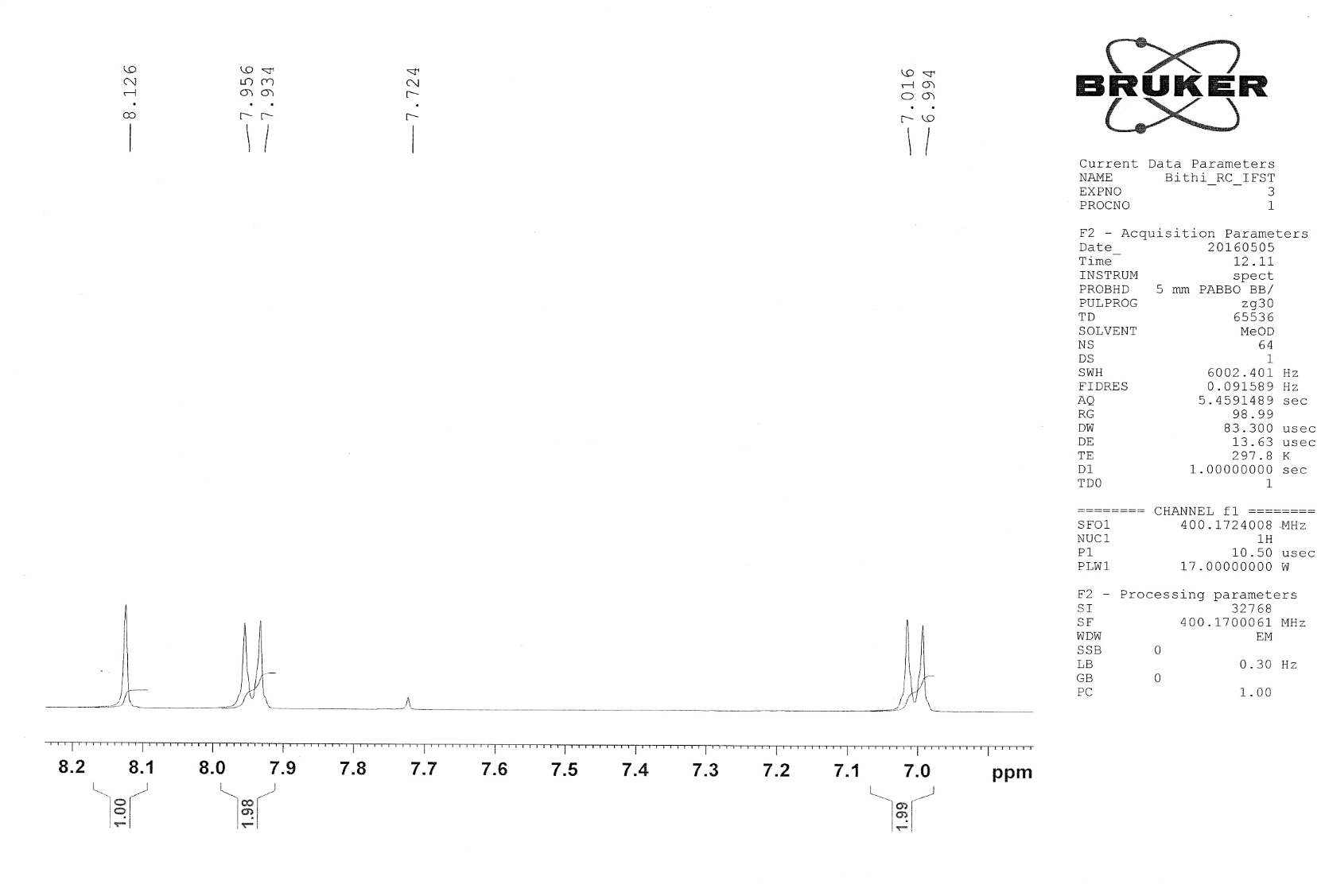 |
| --- |
|  |
| **Fig. S6. ^1^**H NMR spectrum of compound **2**. |

| 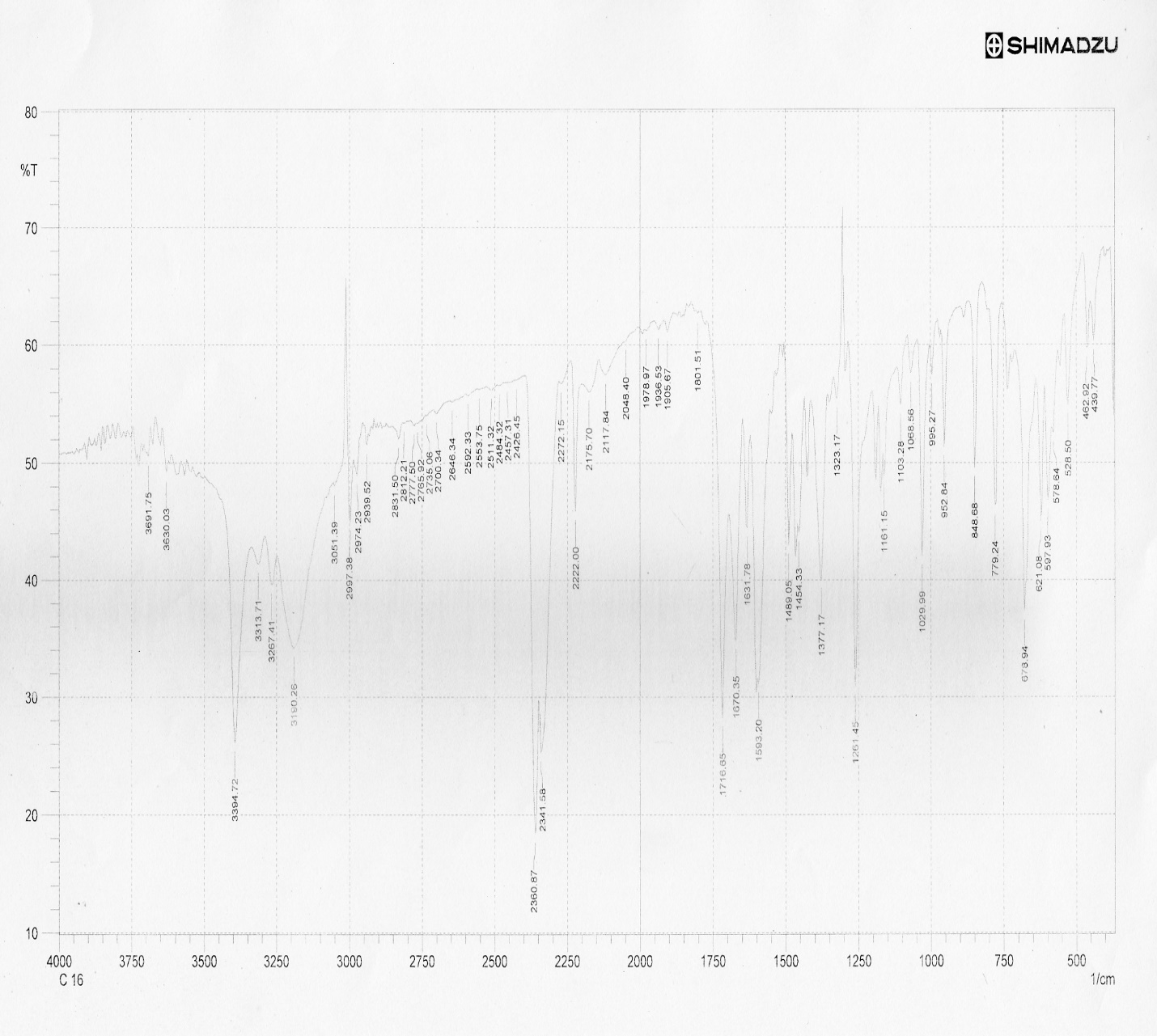 |
| --- |
|  |
| **Fig. S7. FTIR** spectrum of compound **3.** |

| 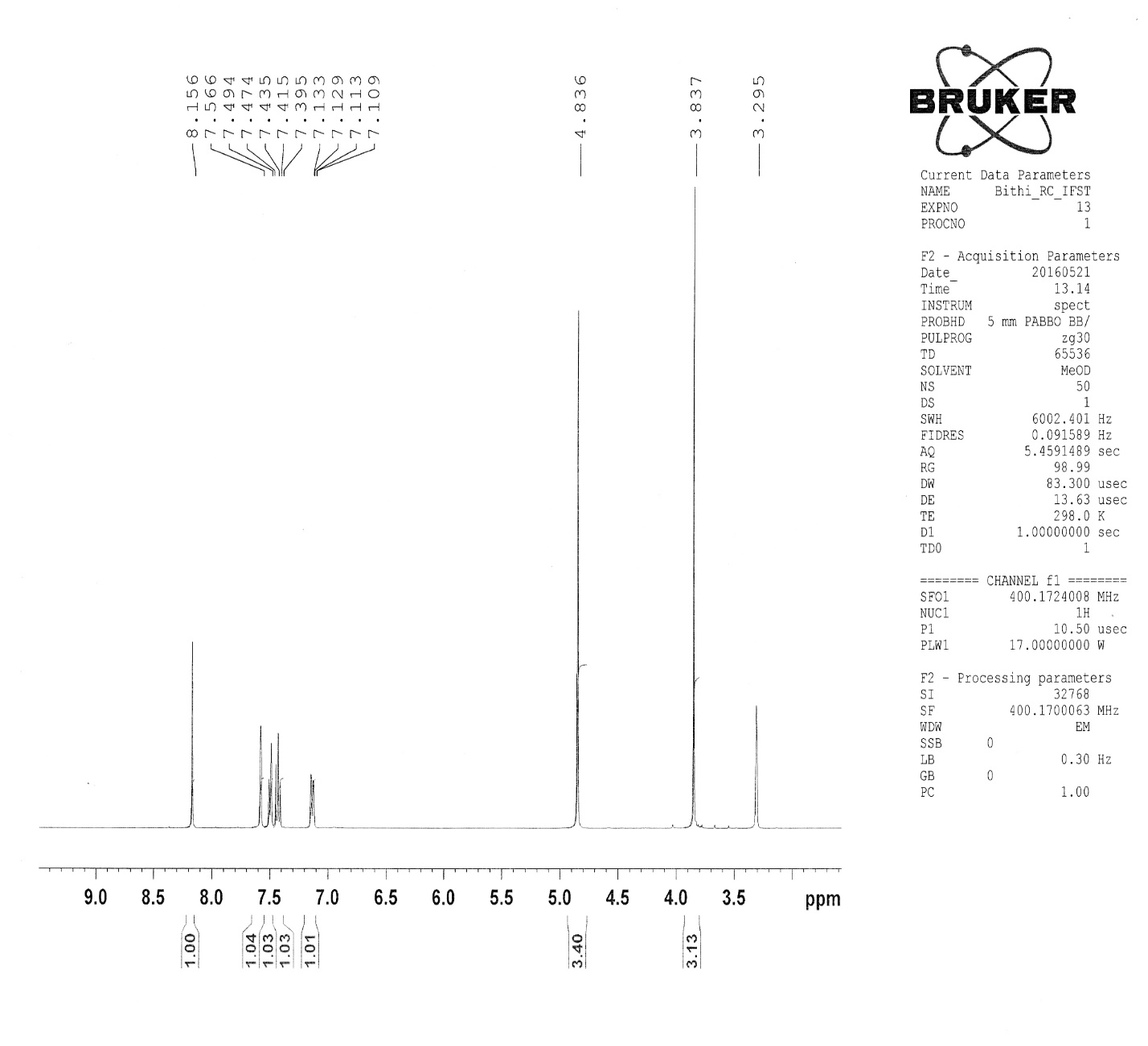 |
| --- |
|  |
| **Fig. S8. ^1^**H NMR spectrum of compound **3**. |

| **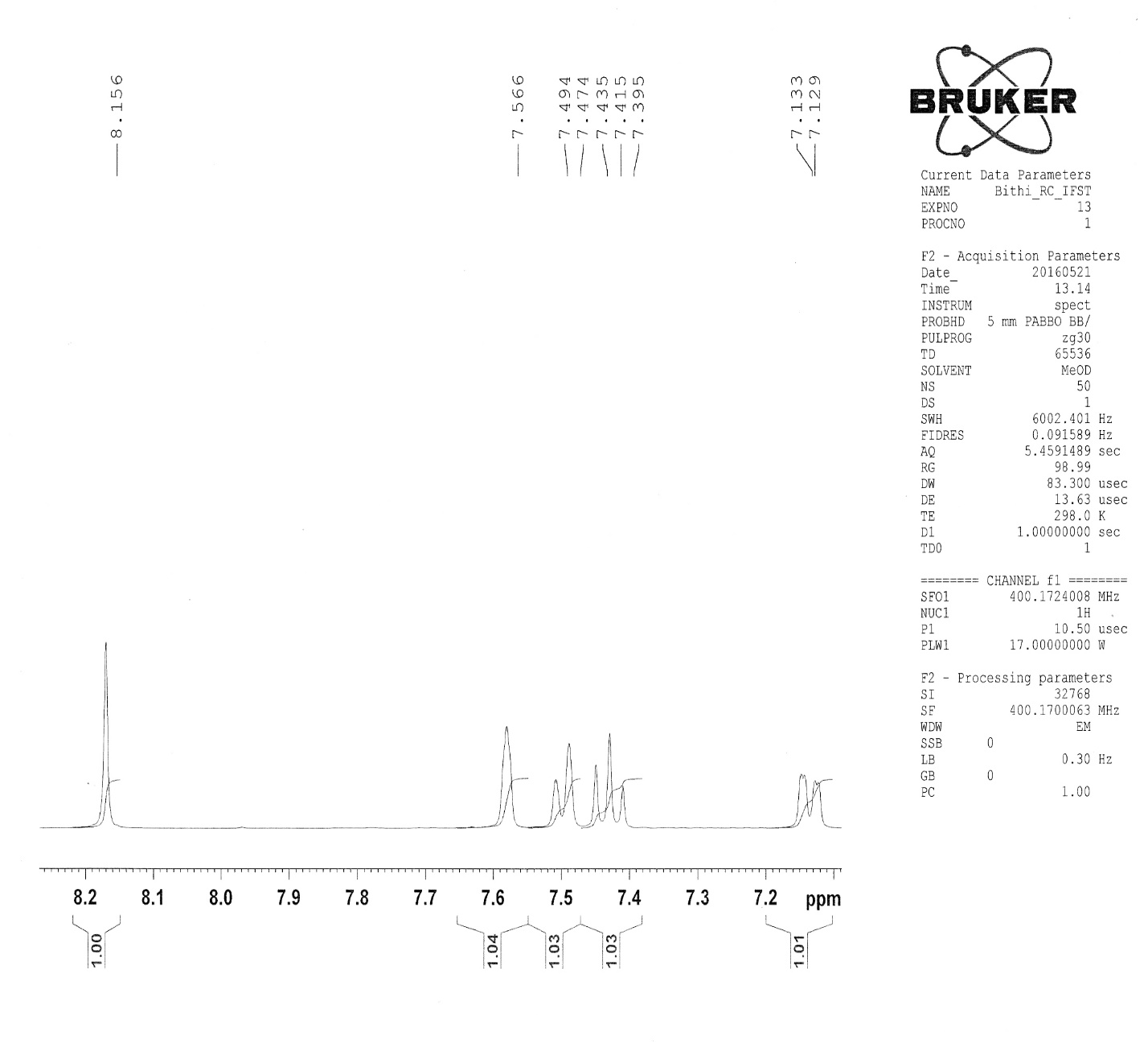** |
| --- |
|  |
| **Fig. S9. ^1^**H NMR spectrum of compound **3**. |

| 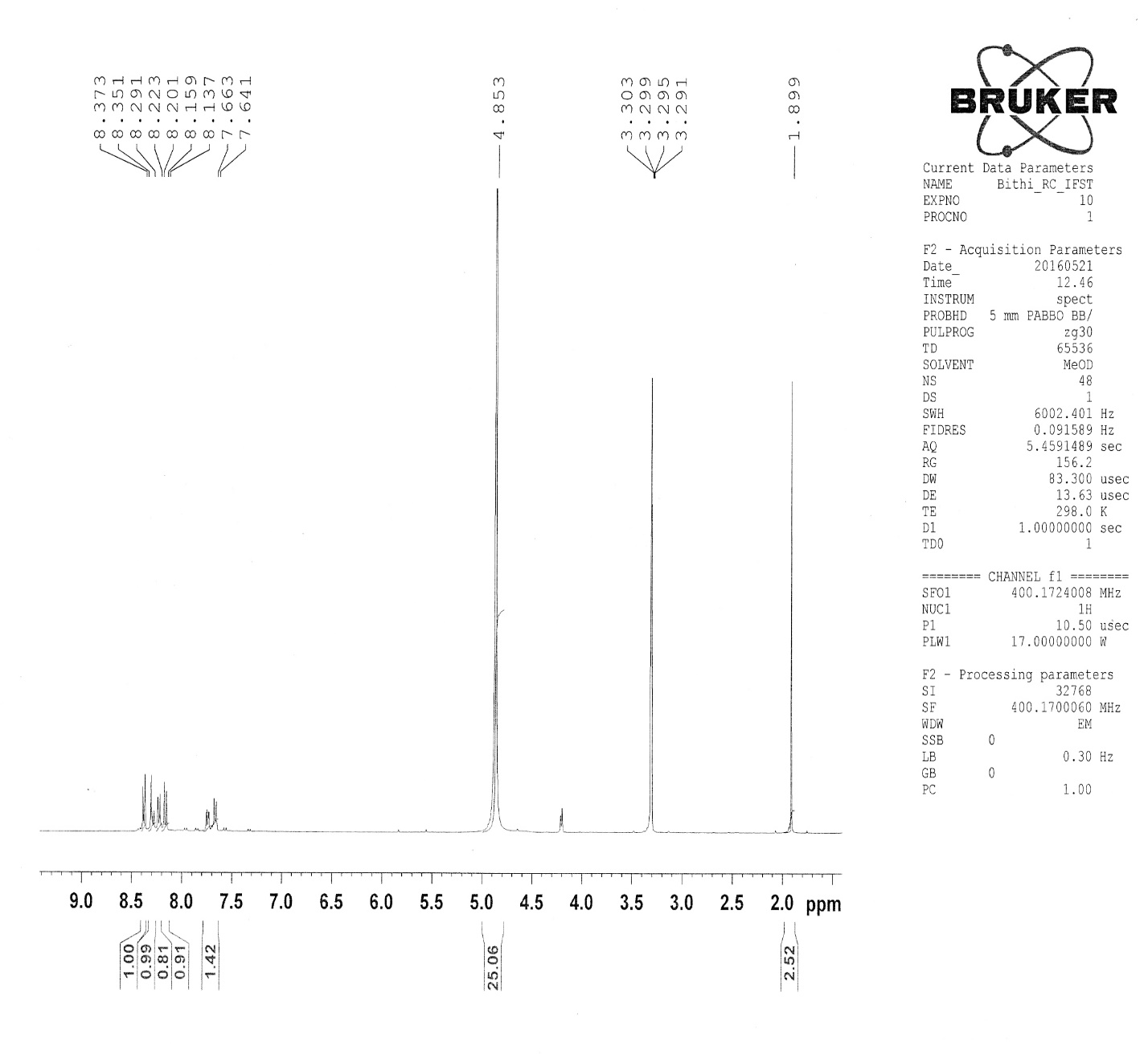 |
| --- |
|  |
| **Fig. S10. ^1^**H NMR spectrum of compound **4**. |

| **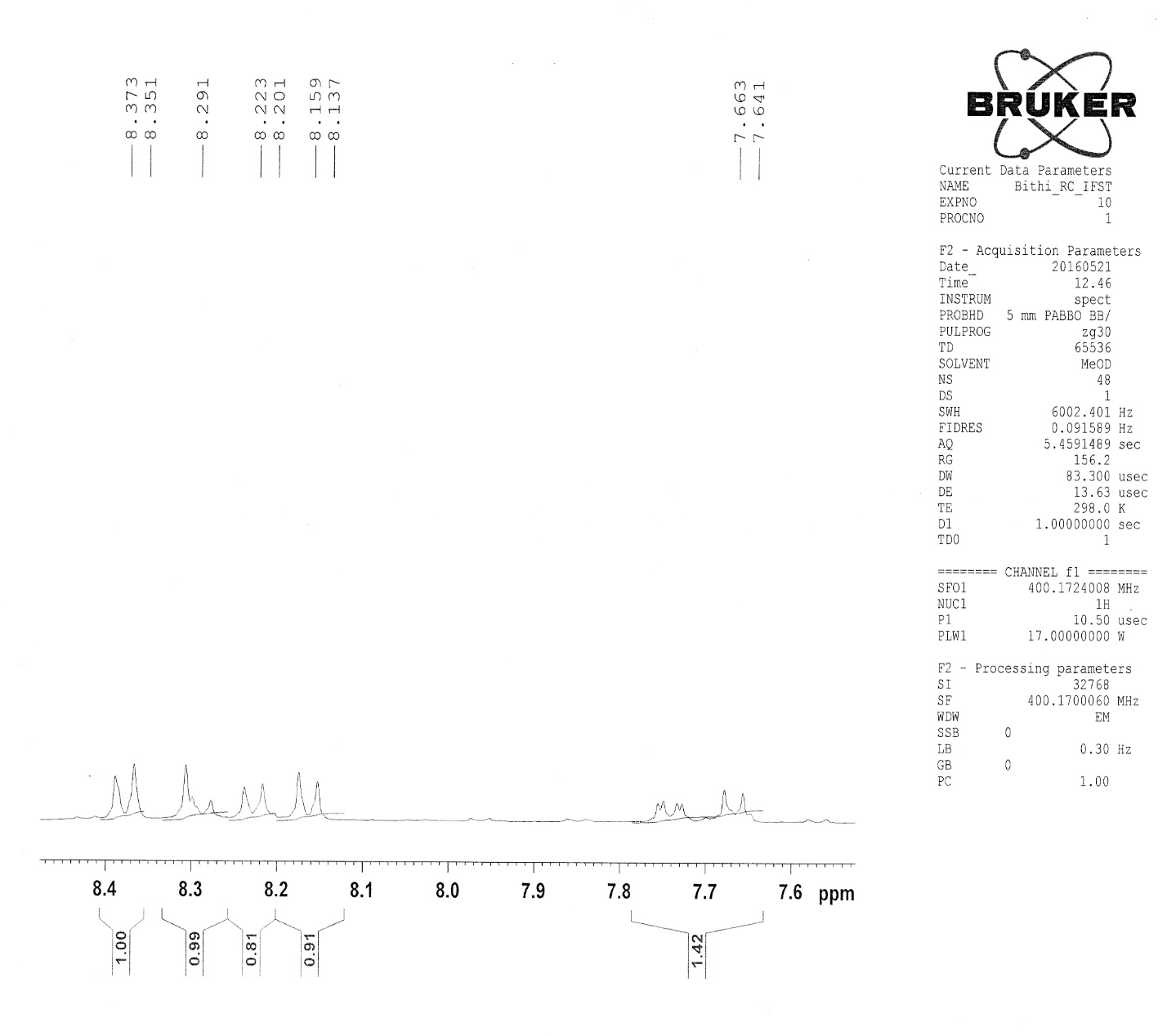** |
| --- |
|  |
| **Fig. S11. ^1^**H NMR spectrum of compound **4**. |

| 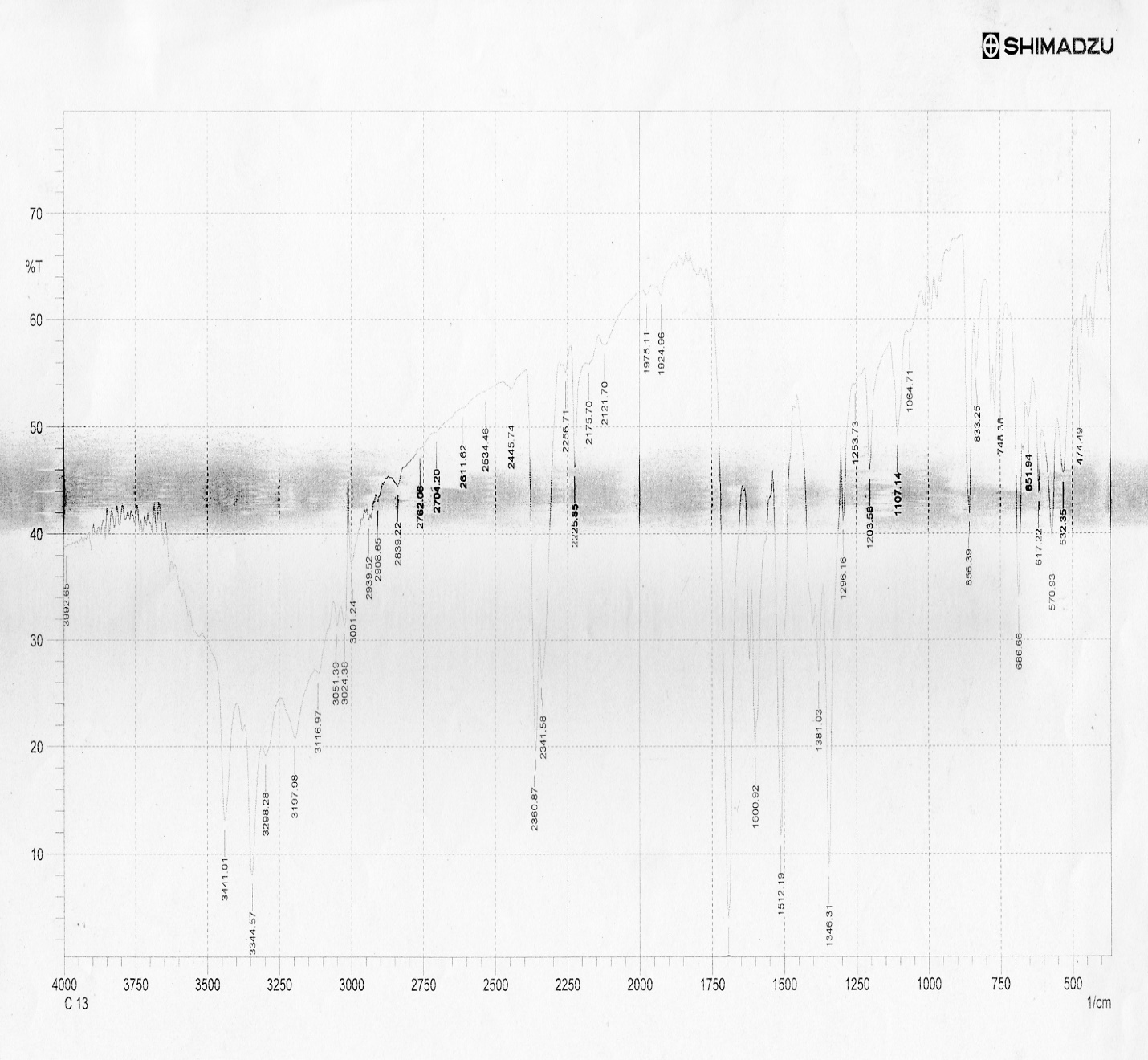 |
| --- |
|  |
| **Fig. S12. FTIR** spectrum of compound **4.** |

| **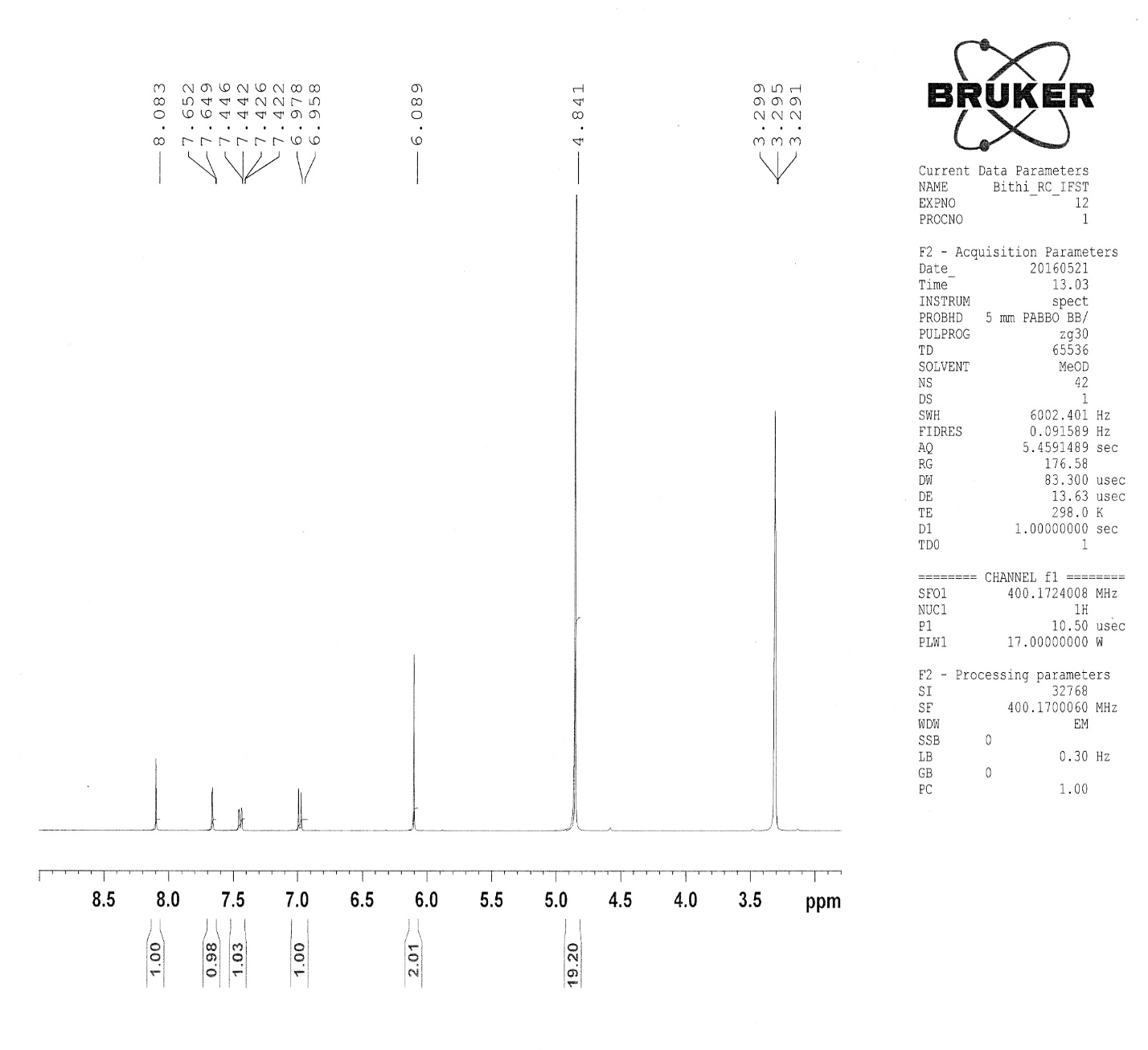** |
| --- |
|  |
| **Fig. S13. ^1^**H NMR spectrum of compound **5**. |

| **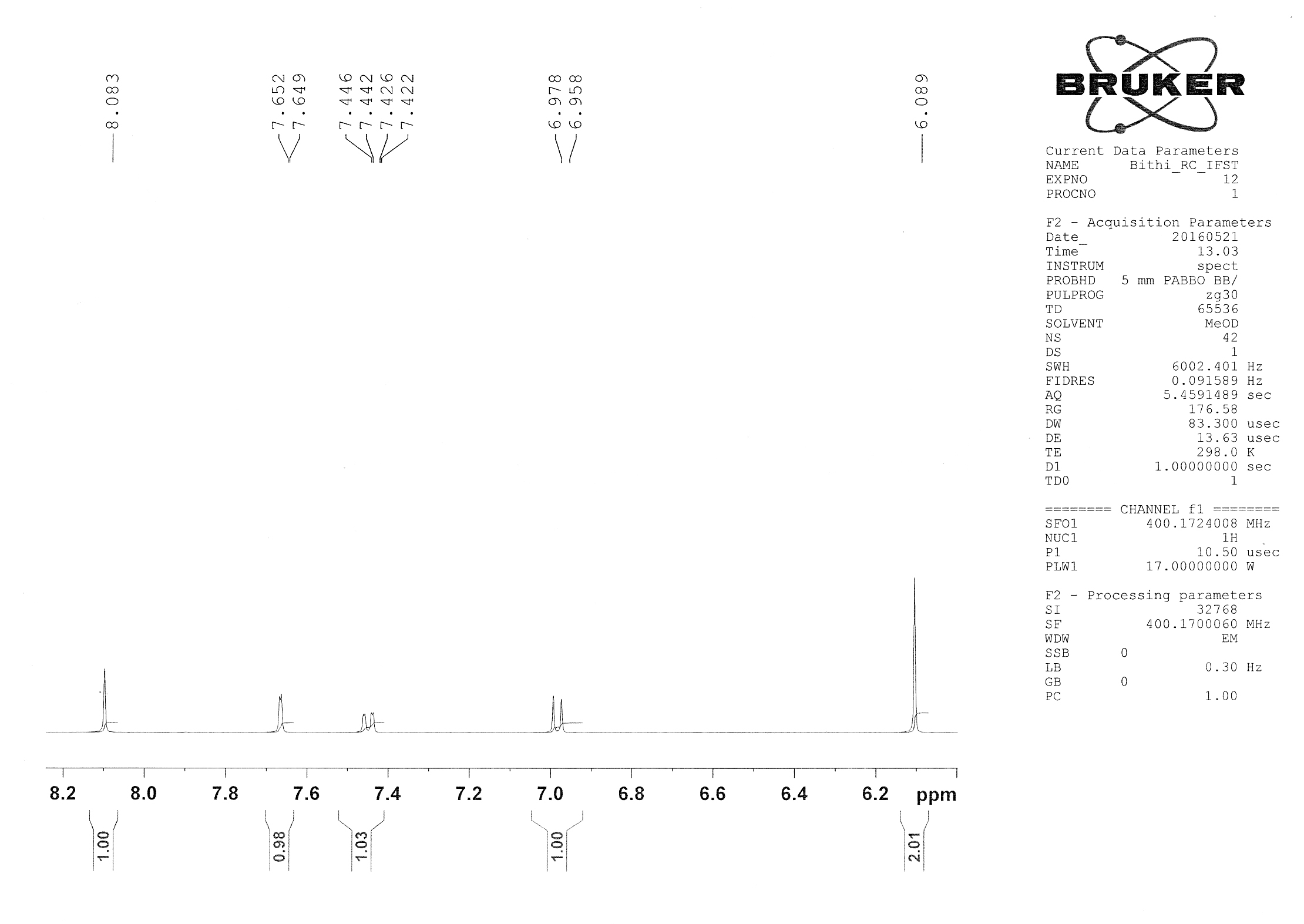** |
| --- |
|  |
| **Fig. S14. ^1^**H NMR spectrum of compound **5**. |

| 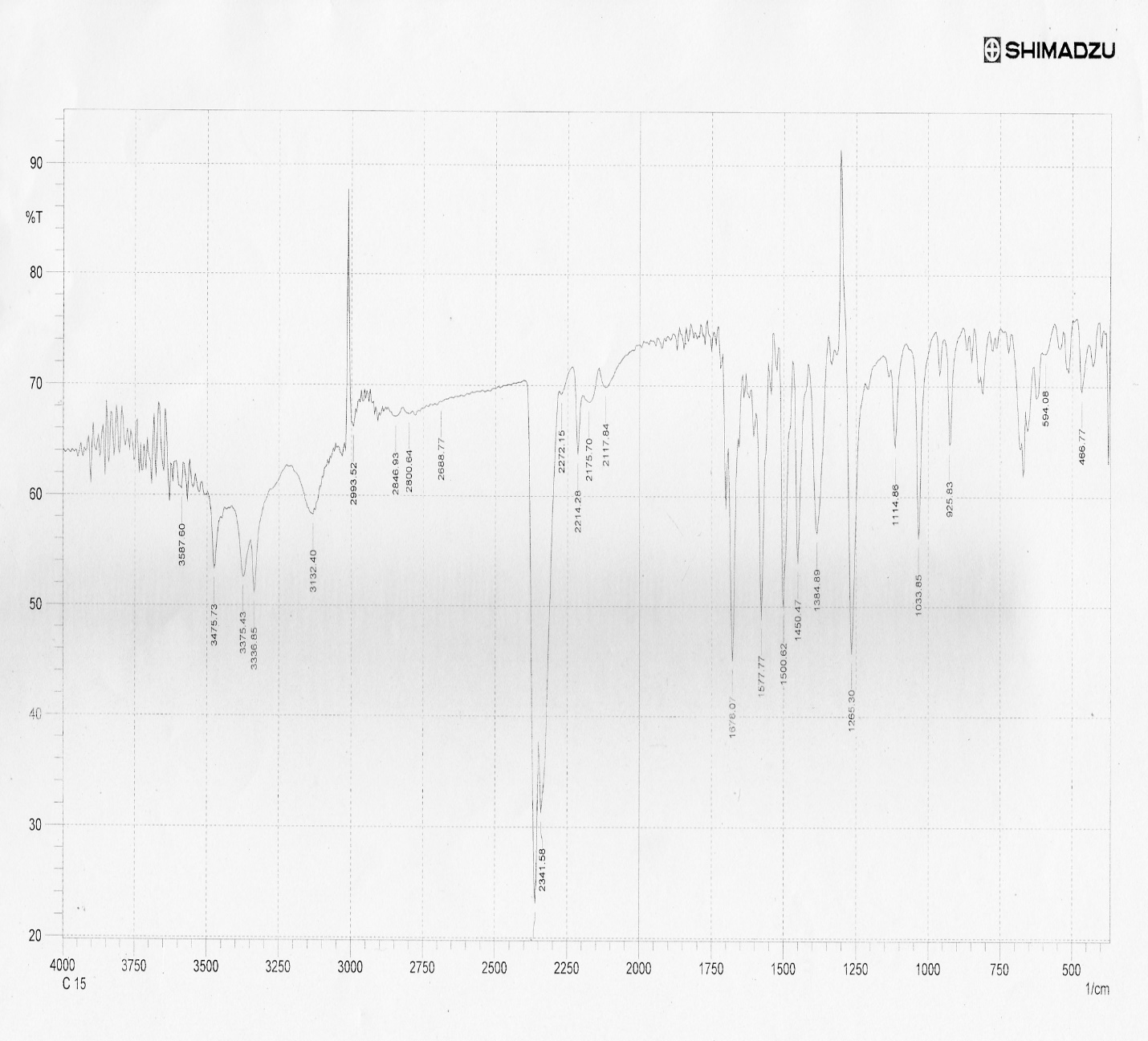 |
| --- |
|  |
| **Fig. S15. FTIR** spectrum of compound **5.** |


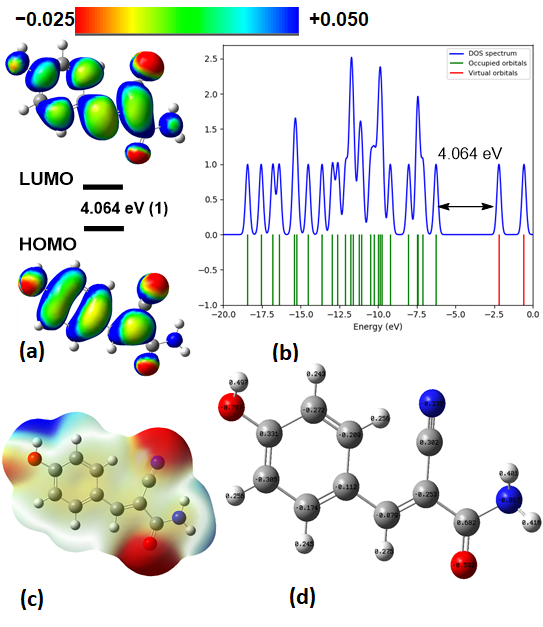


**Fig. S16.** (a) Molecular orbitals of isodensity surfaces (0.02 electrons Bohr−3 surface) (red = electron-rich, blue = electron-deficient) of HOMO and LUMO; (b) DOS plot and HOMO-LUMO energy gap; (c) Maps of electrostatic potential (0.02 electrons Bohr−3 surface) (red = electron-rich, blue = electron-deficient); (d) NBO charges structures for the compound, **1**.


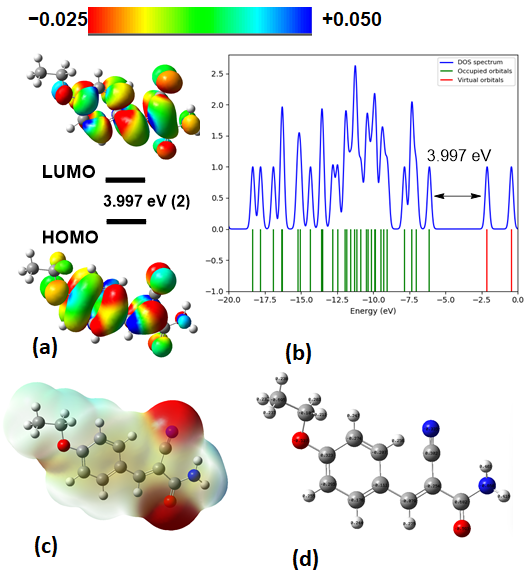


**Fig. S17.** (a) Molecular orbitals of isodensity surfaces (0.02 electrons Bohr^−3^ surface) (red = electron-rich, blue = electron-deficient) of HOMO and LUMO; (b) DOS plot and HOMO-LUMO energy gap; (c) Maps of electrostatic potential (0.02 electrons Bohr^−3^ surface) (red = electron-rich, blue = electron-deficient); (d) NBO charges structures for the compound, **2**.


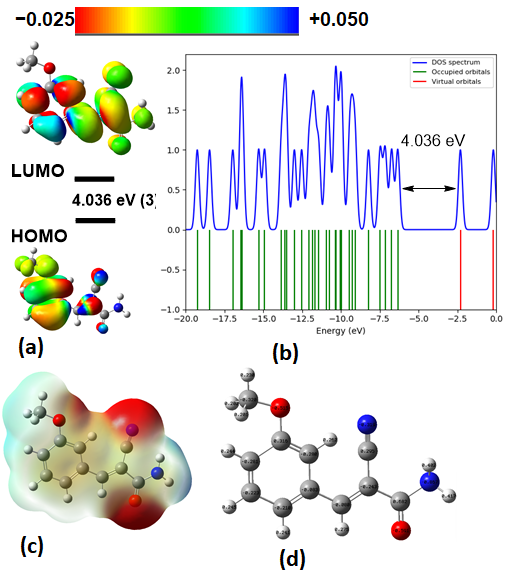


**Fig. S18.** (a) Molecular orbitals of isodensity surfaces (0.02 electrons Bohr^−3^ surface) (red = electron-rich, blue = electron-deficient) of HOMO and LUMO; (b) DOS plot and HOMO-LUMO energy gap; (c) Maps of electrostatic potential (0.02 electrons Bohr^−3^ surface) (red = electron-rich, blue = electron-deficient); (d) NBO charges structures for the compound, **3**.

**
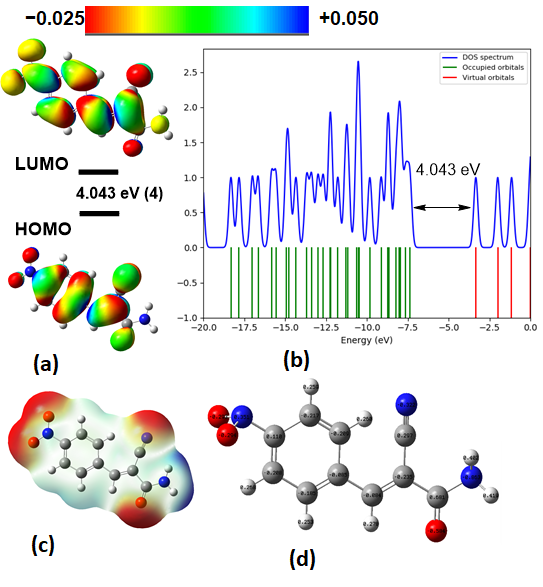
**

**Fig. S19.** Figure 1. (a) Molecular orbitals of isodensity surfaces (0.02 electrons Bohr^−3^ surface) (red = electron-rich, blue = electron-deficient) of HOMO and LUMO; (b) DOS plot and HOMO-LUMO energy gap; (c) Maps of electrostatic potential (0.02 electrons Bohr^−3^ surface) (red = electron-rich, blue = electron-deficient); (d) NBO charges structures for the compound, **4**.

**
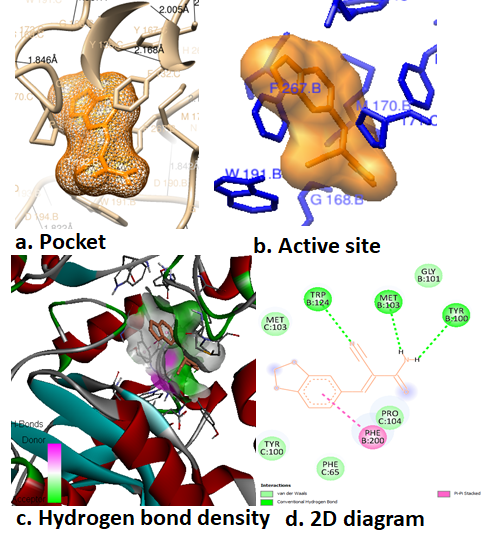
**

**Fig. S20.** Molecular docking results: (a) ligand binding in protein pocket; (b) hydrogen bonding; (c) ligand-protein interaction in 2D diagram; (d) hydrogen bonding in solid state, for compound **5** in 5N1J.


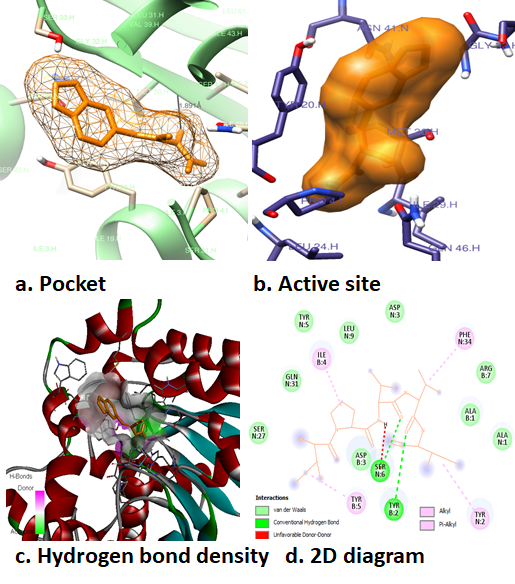


**Fig. S21.** Molecular docking results: (a) ligand binding in protein pocket; (b) hydrogen bonding; (c) ligand-protein interaction in 2D diagram; (d) hydrogen bonding in solid state, for compound **5** in 4NZZ.


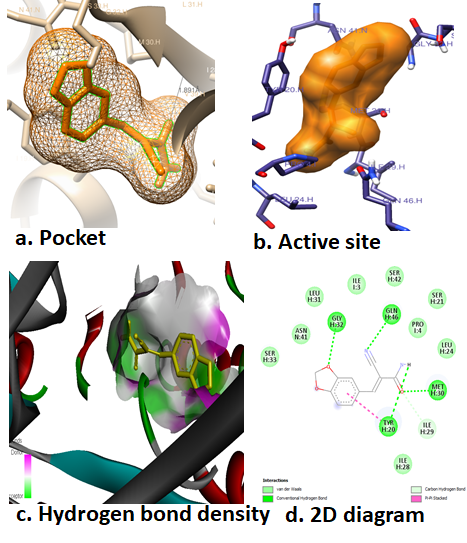


**Fig. S22.** Molecular docking results: (a) ligand binding in protein pocket; (b) hydrogen bonding; (c) ligand-protein interaction in 2D diagram; (d) hydrogen bonding in solid state, for compound **5** in 7FEQ.


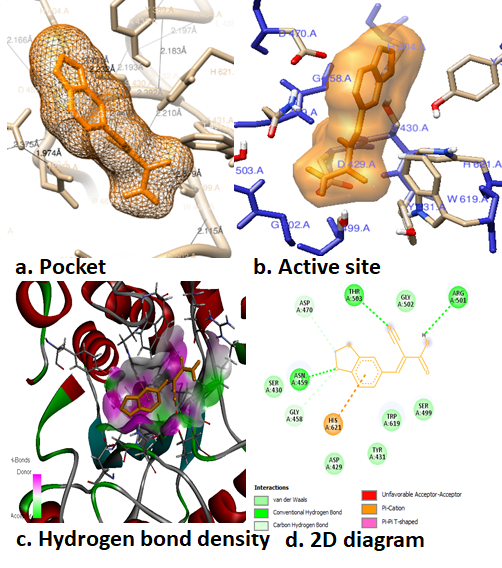


**Fig. S23.** Molecular docking results: (a) ligand binding in protein pocket; (b) hydrogen bonding; (c) ligand-protein interaction in 2D diagram; (d) hydrogen bonding in solid state, for compound **5** in 6SE1.


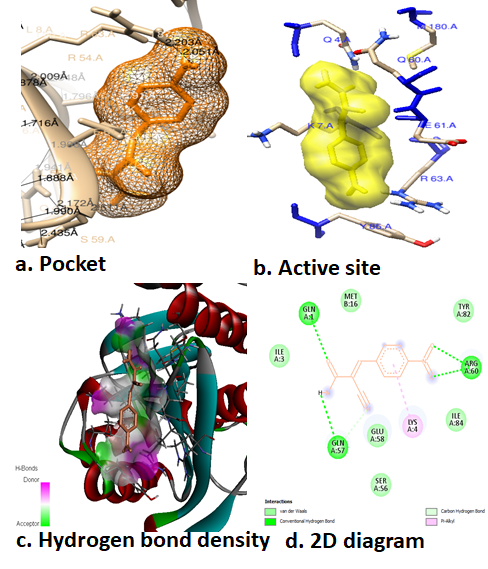


**Fig. S24.** Molecular docking results: (a) ligand binding in protein pocket; (b) hydrogen bonding; (c) ligand-protein interaction in 2D diagram; (d) hydrogen bonding in solid state, for compound **5** in 5GVZ.


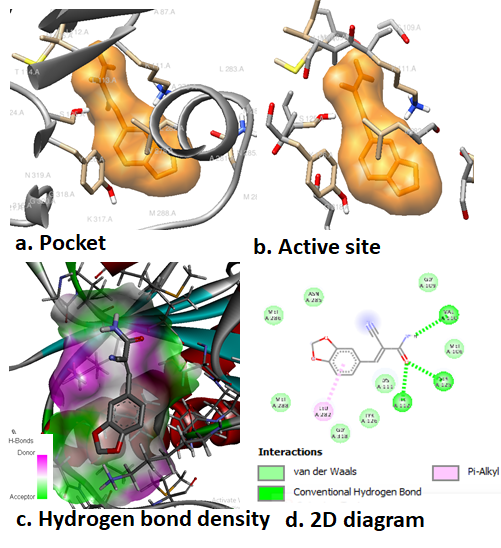


**Fig. S25.** Molecular docking results: (a) ligand binding in protein pocket; (b) hydrogen bonding; (c) ligand-protein interaction in 2D diagram; (d) hydrogen bonding in solid state, for compound **5** in 1TM2.

**
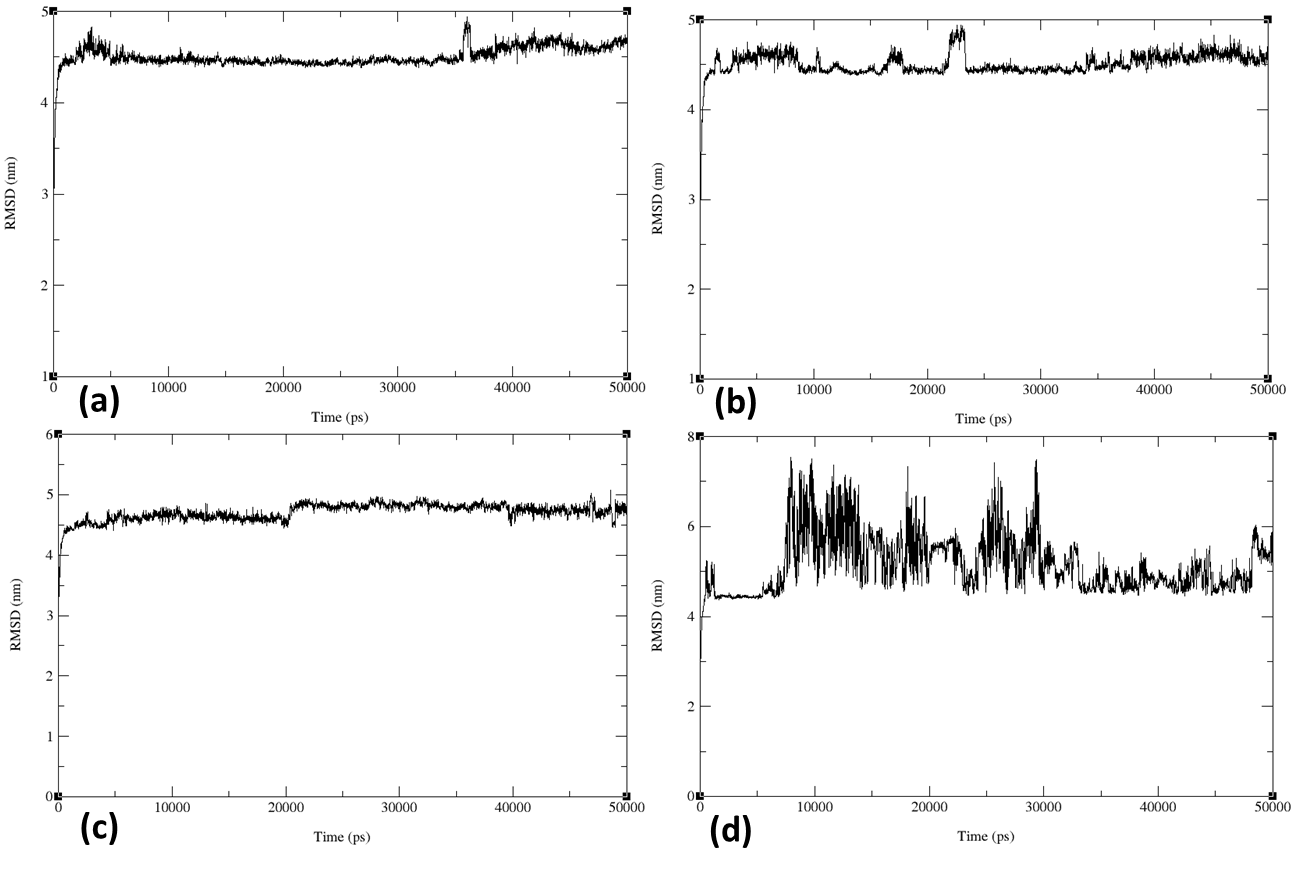
**

**Fig. S26.** RMSD evolution (a) for the ligand, **5**, at 300 K; (b) for the ligand, **5**, at 305 K; (b) for the ligand, **5**, at 310 K; and (d) for the ligand, **5**, at 320 K; during the 50 ns MD simulation.

**
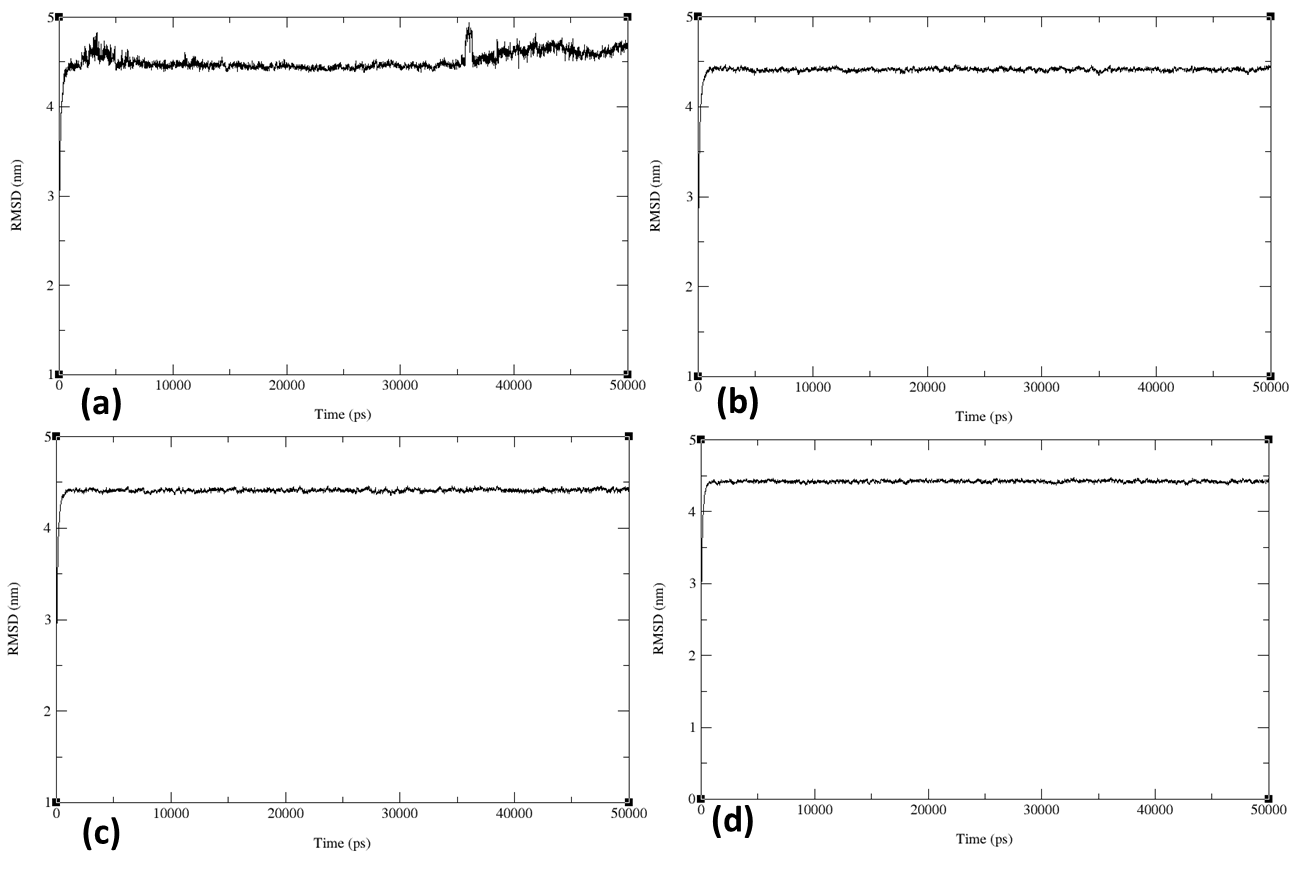
**

**Fig. S27.** RMSD evolution (a) for the ligand, **5**, at 300 K; (b) for the target protein (ID: 5MM8) at 305 K; (b) for the protein (ID: 5MM8) at 310 K; and (d) for the protein, (ID: 5MM8) at 320 K; during the 50 ns MD simulation.


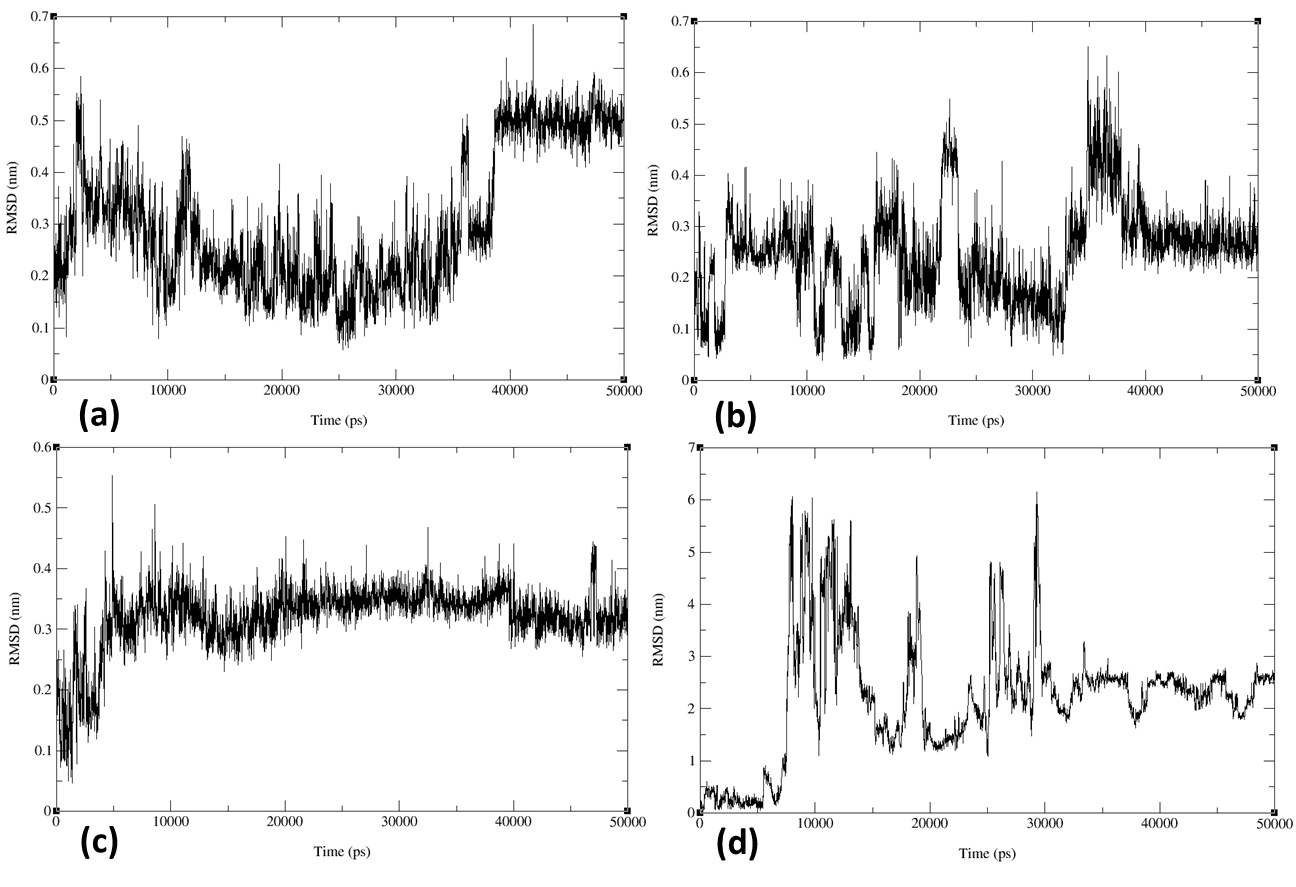


**Fig. S28.** RMSD evolution (a) for the protein-ligand complex, at 300 K; (b) for the protein-ligand complex at 305 K; (b) for the protein-ligand complex at 310 K; and (d) for the protein-ligand complex at 320 K; during the 50 ns MD simulation.


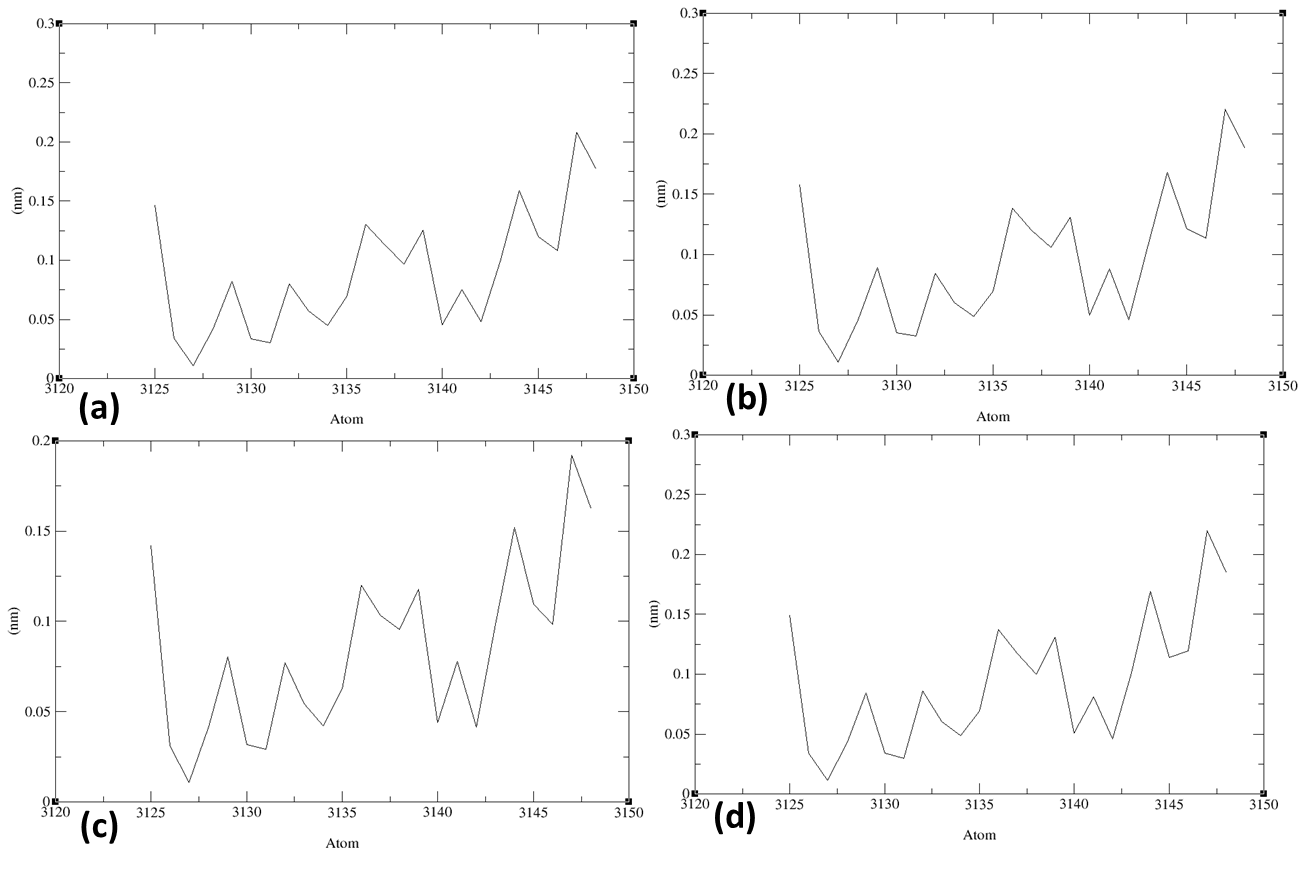


**Fig. S29.** RMSF evolution (a) for the ligand, **5**, at 300 K; (b) for the ligand, **5**, at 305 K; (b) for the ligand, **5**, at 310 K; and (d) for the ligand, **5**, at 320 K; during the 50 ns MD simulation.


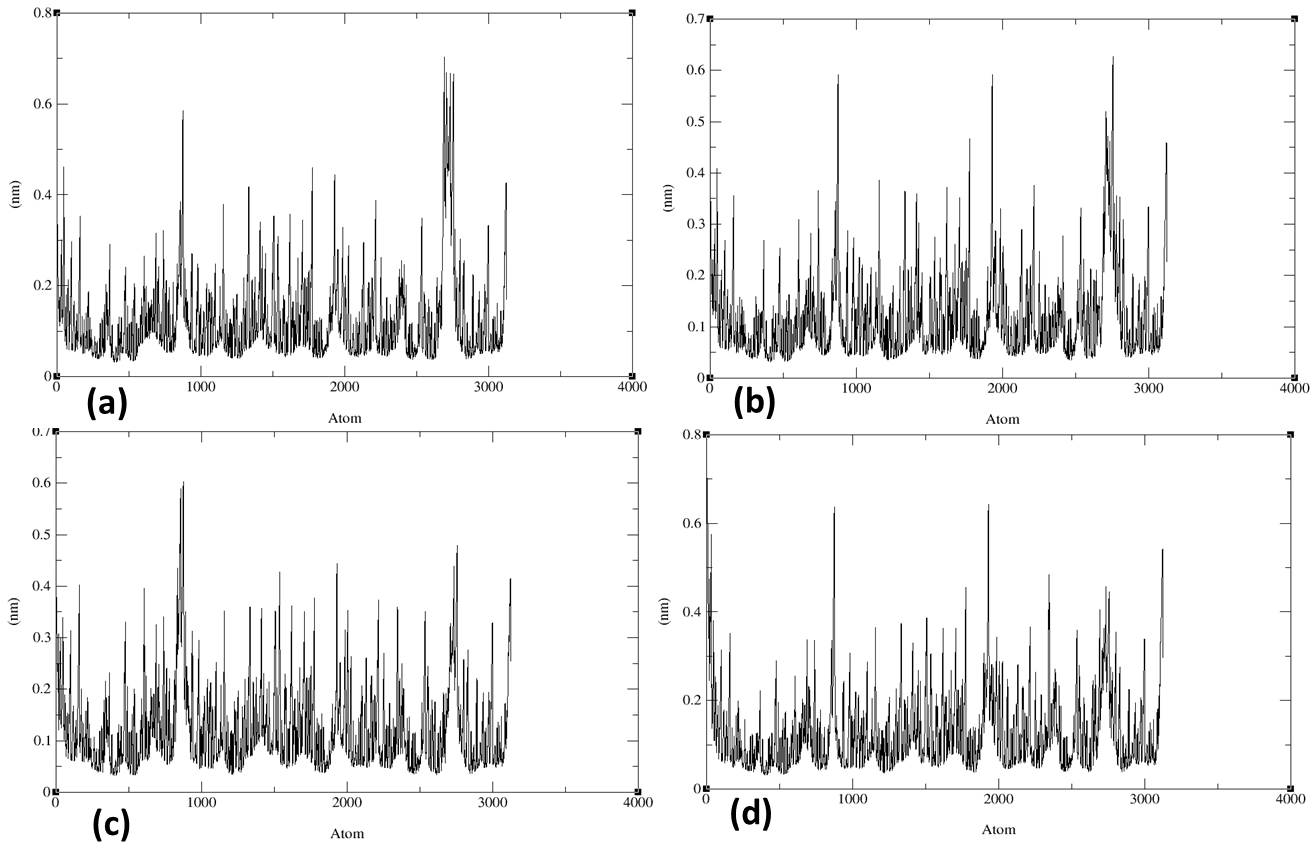


**Fig. S30.** RMSF evolution (a) for the ligand, **5**, at 300 K; (b) for the target protein (ID: 5MM8) at 305 K; (b) for the protein (ID: 5MM8) at 310 K; and (d) for the protein, (ID: 5MM8) at 320 K; during the 50 ns MD simulation.


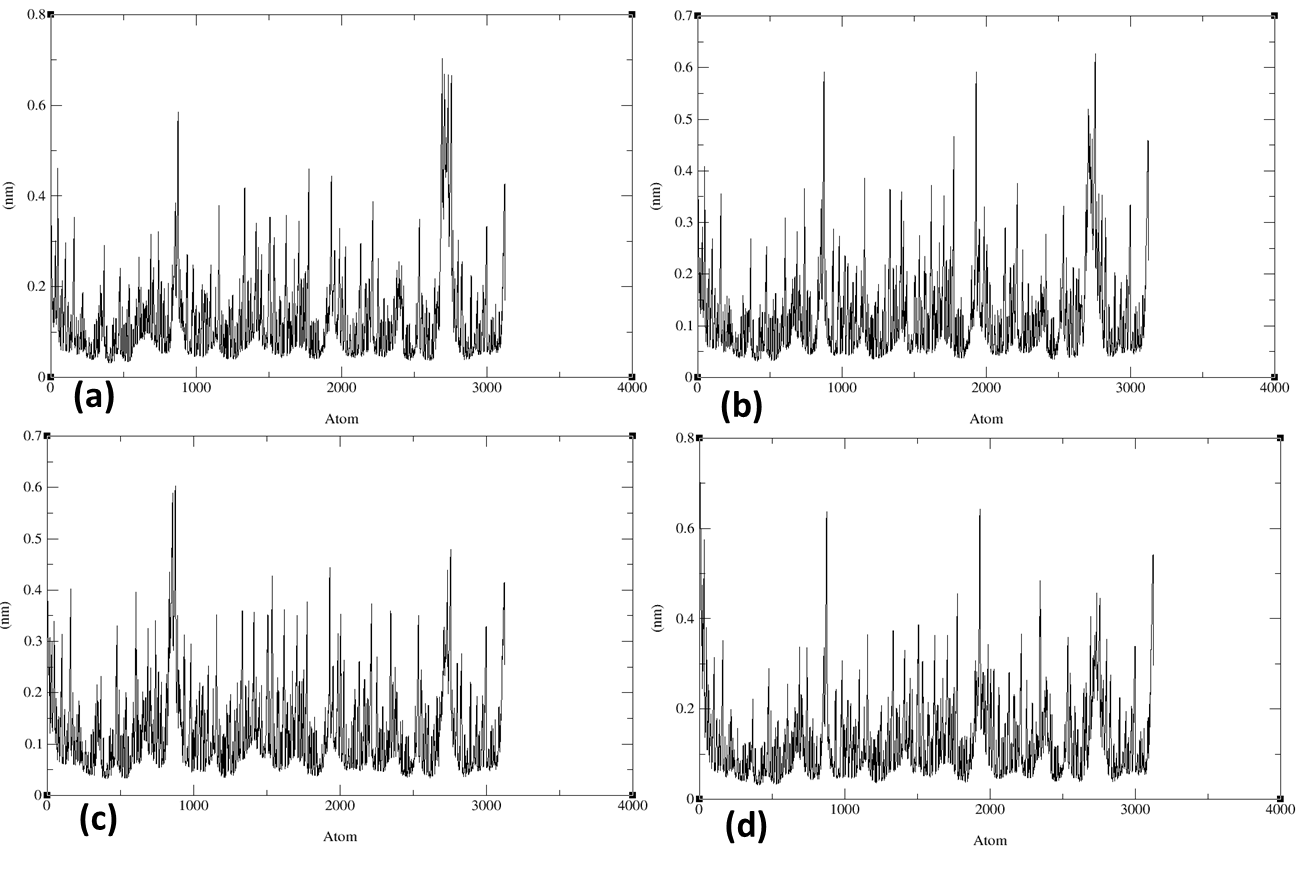


**Fig. S31.** RMSF evolution (a) for the protein-ligand complex, at 300 K; (b) for the protein-ligand complex at 305 K; (b) for the protein-ligand complex at 310 K; and (d) for the protein-ligand complex at 320 K; during the 50 ns MD simulation.


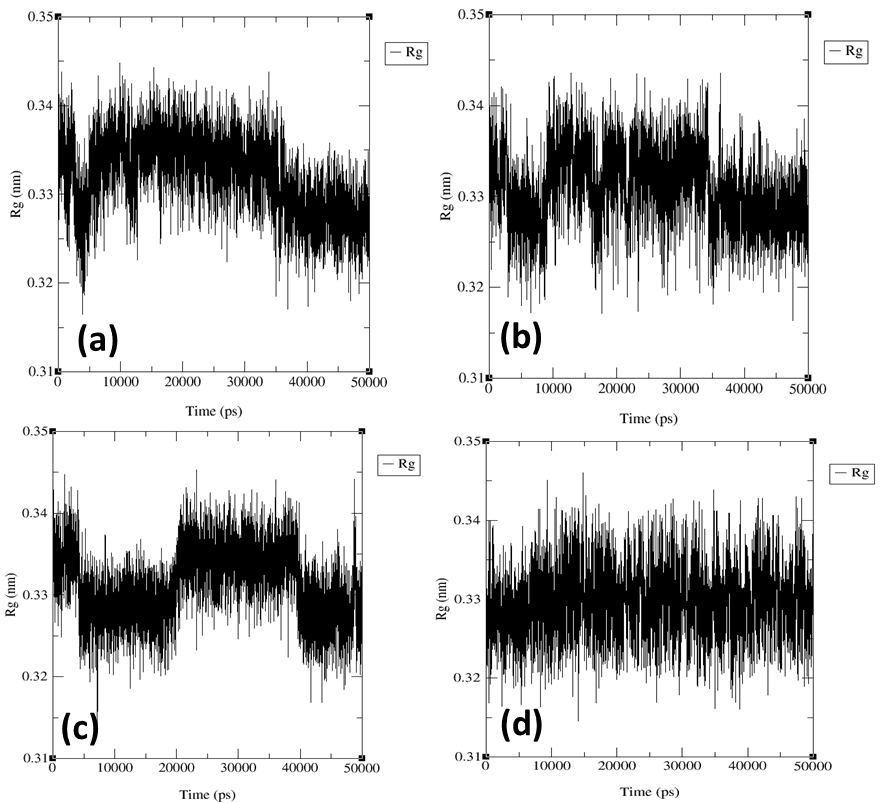


**Fig. S32.** Rg evolution (a) for the ligand, **5**, at 300 K; (b) for the ligand, **5**, at 305 K; (b) for the ligand, **5**, at 310 K; and (d) for the ligand, **5**, at 320 K; during the 50 ns MD simulation.


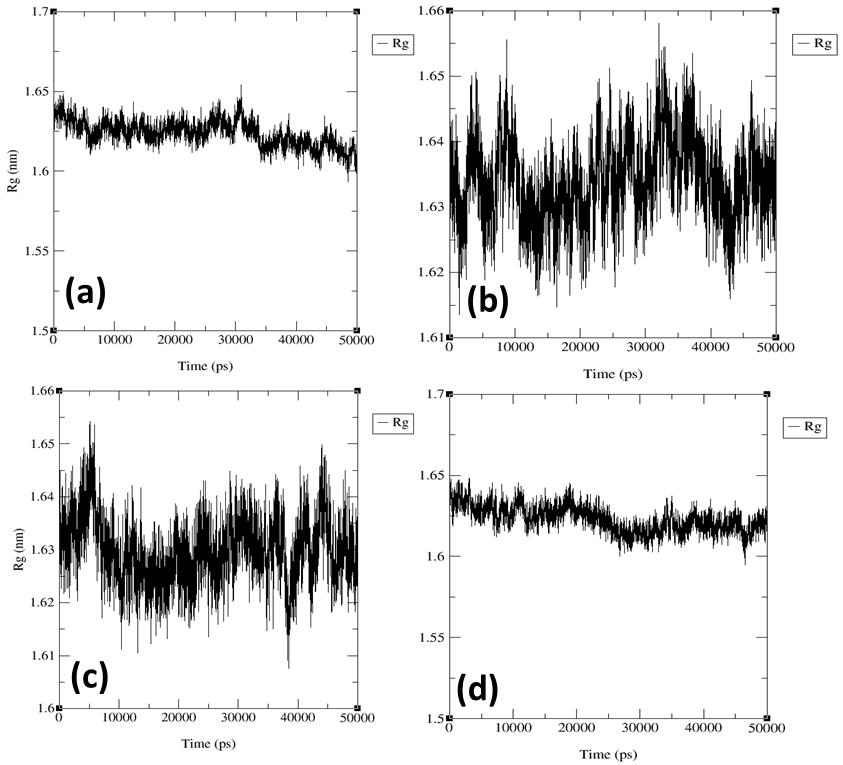


**Fig. S33.** Rg evolution (a) for the ligand, **5**, at 300 K; (b) for the target protein (ID: 5MM8) at 305 K; (b) for the protein (ID: 5MM8) at 310 K; and (d) for the protein, (ID: 5MM8) at 320 K; during the 50 ns MD simulation.


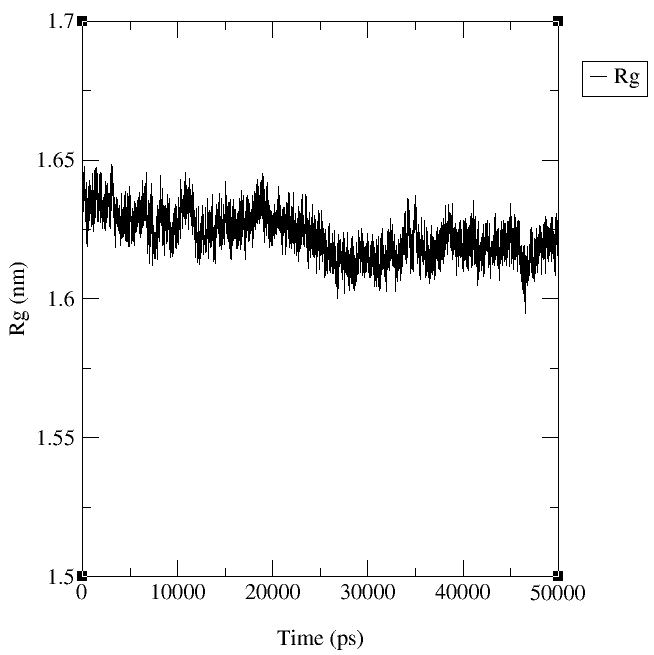


**(d)**


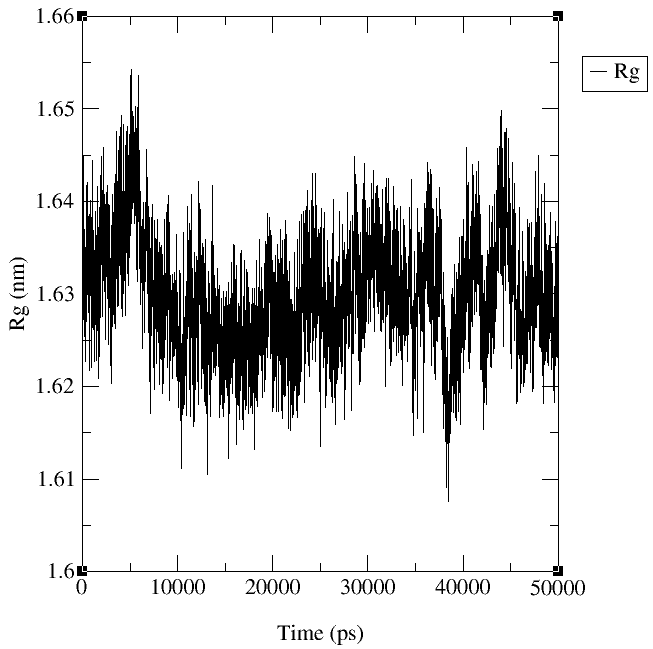


**(c)**


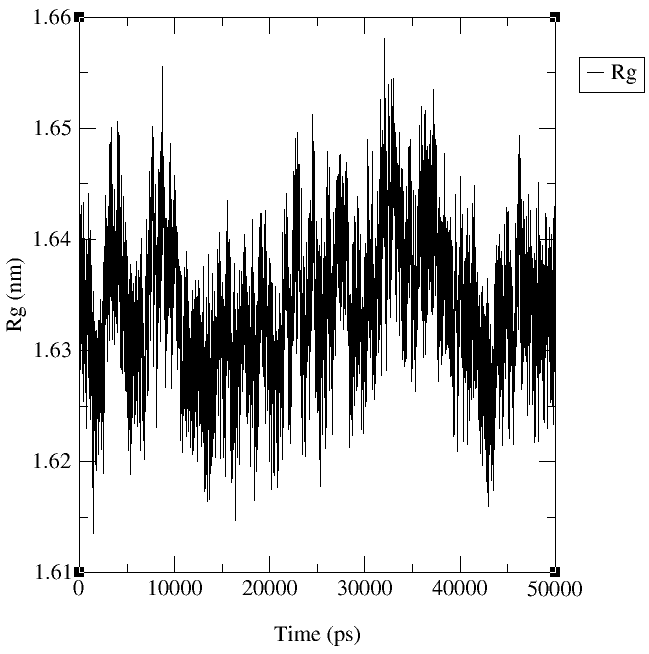


**(b)**


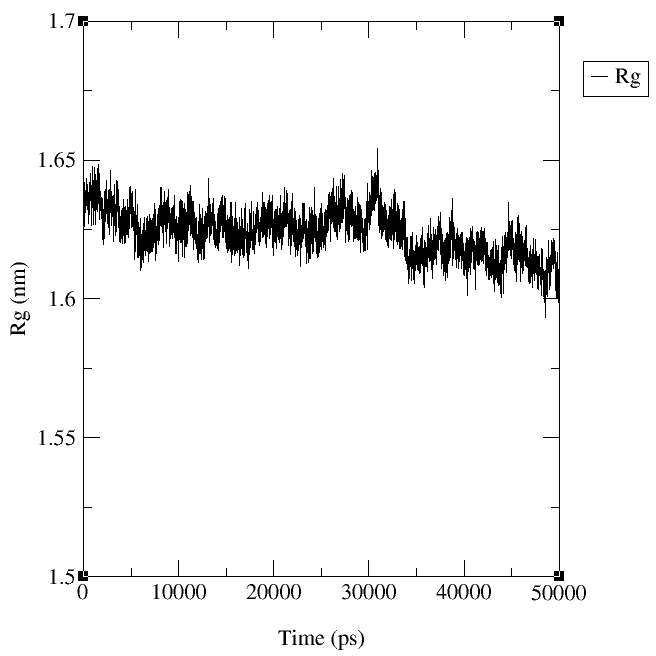


**(a)**

**Fig. S34.** Rg evolution (a) for the protein-ligand complex, at 300 K; (b) for the protein-ligand complex at 305 K; (b) for the protein-ligand complex at 310 K; and (d) for the protein-ligand complex at 320 K; during the 50 ns MD simulation.


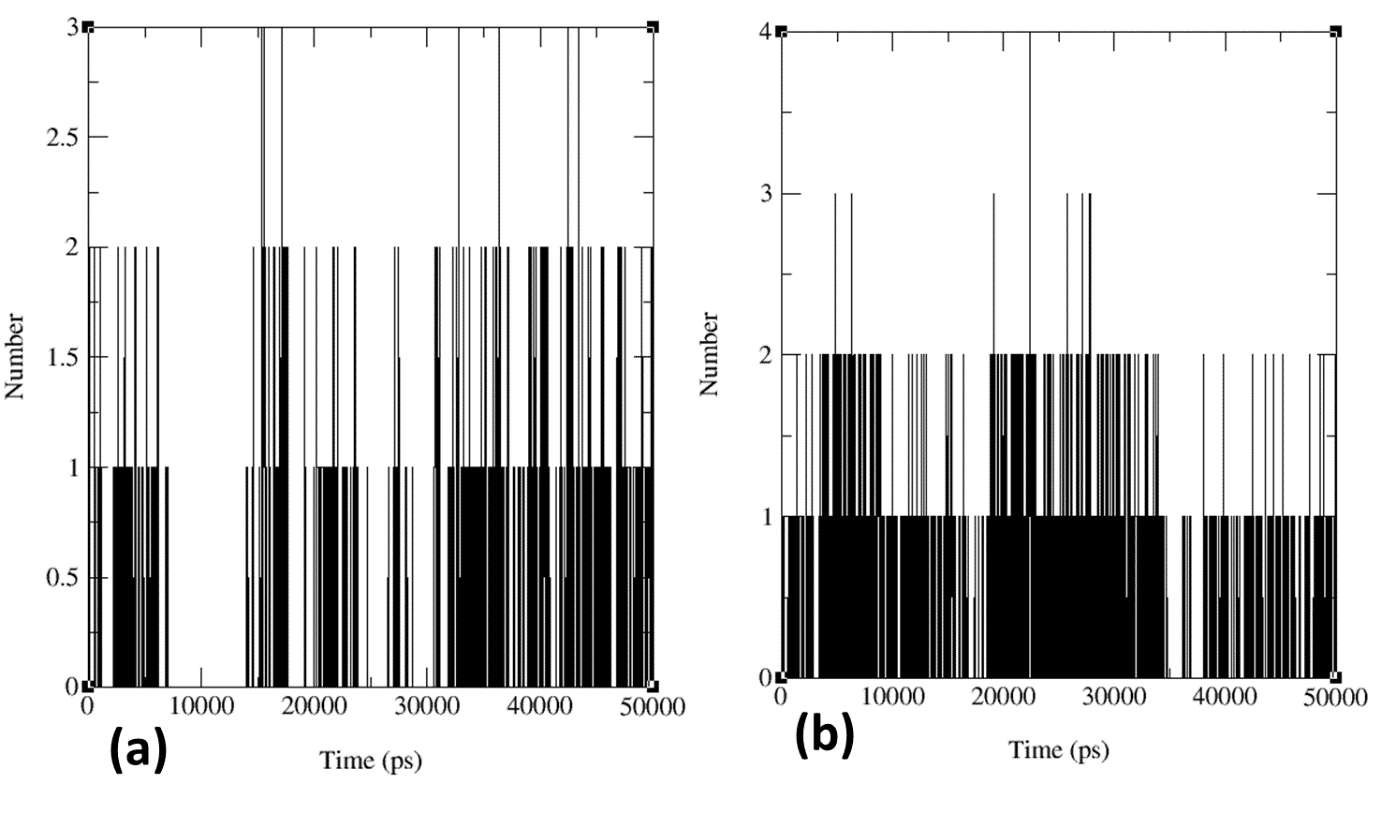


| **Fig. S35.** Intermolecular hydrogen bonding (HBs) stabilization progression for combined protein (ID:5MM8) and ligand **5** under two different temperature conditions; (e) Intermolecular hydrogen bonding stabilization at 300 K; and (f) Intermolecular hydrogen bonding stabilization at 305 K during the 50 ns MD simulation. |
| --- |
